# Novel Inhibitors of Nicotinamide-*N*-Methyltransferase for the Treatment of Metabolic Disorders

**DOI:** 10.3390/molecules26040991

**Published:** 2021-02-13

**Authors:** Aimo Kannt, Sridharan Rajagopal, Mahanandeesha S. Hallur, Indu Swamy, Rajendra Kristam, Saravanakumar Dhakshinamoorthy, Joerg Czech, Gernot Zech, Herman Schreuder, Sven Ruf

**Affiliations:** 1 Fraunhofer Institute for Translational Medicine and Pharmacology-ITMP, Theodor-Stern-Kai 7, 60596 Frankfurt am Main, Germany; aimo.kannt@itmp.fraunhofer.de; 2 Jubilant Biosys Limited, #96, Industrial Suburb, 2nd Stage Yeshwanthpur, Bangalore 560022, India; Sridharan.Rajagopal@jubilanttx.com (S.R.); Mahanandeesha.Hallur@jubilantbiosys.com (M.S.H.); Indu.Swamy@jubilantbiosys.com (I.S.); Rajendra.Kristam@jubilantbiosys.com (R.K.); Saravanakumar.Dhakshinamoorthy@jubilantbiosys.com (S.D.); 3 Sanofi-Aventis Deutschland Gmbh, Industriepark Hoechst, 65926 Frankfurt am Main, Germany; Joerg.Czech@sanofi.com (J.C.); Gernot.Zech@sanofi.com (G.Z.); Herman.Schreuder@sanofi.com (H.S.)

**Keywords:** nicotinamide-*N*-methyltransferase, small molecule inhibitor, nicotinamide, metabolic disease, X-ray

## Abstract

Nicotinamide-*N*-methyltransferase (NNMT) is a cytosolic enzyme catalyzing the transfer of a methyl group from *S*-adenosyl-methionine (SAM) to nicotinamide (Nam). It is expressed in many tissues including the liver, adipose tissue, and skeletal muscle. Its expression in several cancer cell lines has been widely discussed in the literature, and recent work established a link between NNMT expression and metabolic diseases. Here we describe our approach to identify potent small molecule inhibitors of NNMT featuring different binding modes as elucidated by X-ray crystallographic studies.

## 1. Introduction

Nicotinamide-*N*-methyltransferase (NNMT) is a cytosolic enzyme that catalyzes the transfer of a methyl group from *S*-adenosyl-methionine (SAM) to nicotinamide (Nam), yielding *S*-adenosyl-homocysteine (SAH) and 1-methylnicotinamide (MNam) [1,2]. It is expressed in most tissues including the liver, adipose tissue, and skeletal muscle [3], and also in several human cancers [4]. NNMT is the major Nam-metabolizing enzyme, and systemic knockout in mice fully prevents MNam formation [5,6]. MNam can either be excreted via urine or further metabolized to 1-methyl-2-pyridone-5-carboxamide (Me2PY) or 1-methyl-4-pyridone-5-carboxamide (Me4PY) in a reaction catalyzed by aldehyde oxidase [7].

Higher NNMT expression and activity in white adipose tissue of mice [8] and humans [9,10] has been associated with obesity, insulin resistance and type-2 diabetes (T2D). Plasma, serum or urinary MNam levels were found to correlate with obesity or T2D in individuals from different geographic regions [10,11,12]. Interventions to improve metabolic health such as exercise and bariatric surgery were shown to decrease adipose NNMT expression and plasma MNam levels in obese individuals with insulin resistance or T2D [10]. Single-nucleotide polymorphisms of NNMT have been associated with BMI [13], hyperhomocysteinemia [14], non-alcoholic steatohepatitis [15], and hyperlipidemia [16]. 

**Figure 1:**
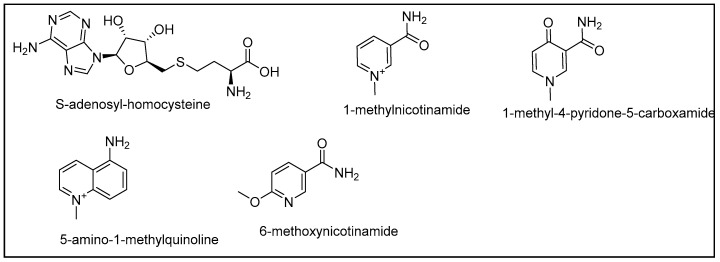
Known NNMT inhibitors.

Reduction in hepatic and adipose tissue NNMT with an antisense oligonucleotide led to lower weight gain, a decrease in relative fat mass, lower plasma insulin levels and improved glucose tolerance in mice on a high-fat diet (HFD) [6,8]. Likewise, treatment with the selective NNMT inhibitors 5-amino-1-methylquinoline [17] or 6-methoxynicotinamide [5] resulted in reduced body weight, higher insulin sensitivity, and improved glucose tolerance in mice with diet-induced obesity (Figure 1).

Additional types of NNMT inhibitors that have recently been reported include other nicotinamide-related compounds such as 6-methylaminonicotinamide [18], bisubstrate-like inhibitors based on adenosyl scaffolds [19,20,21,22,23], and alpha-chloroacetamide-based compounds covalently binding to a cysteine residue in the SAM-binding pocket of the catalytic site [24].

**Figure 2:**
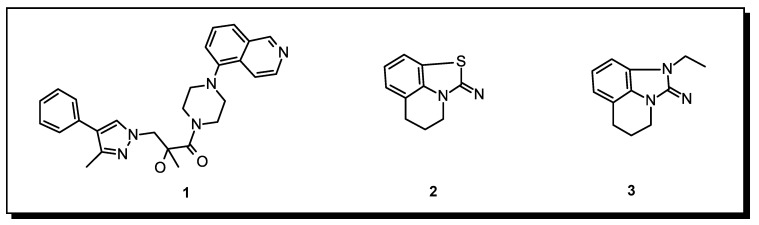
HTS hits identified from Sanofi compound collection.

Here we describe two novel types of NNMT inhibitors: (A) The 3-methyl-4-phenylpyrazole series represented by (**1**), which occupies both the Nam and SAM pockets of the catalytic center and is the first non-adenosyl bisubstrate inhibitor series with nanomolar potency. (B) A series of tricyclic compounds represented by (**2**), (**3**) with high selectivity for NNMT and strong inhibitory activity on the isolated enzyme (Figure 2).

Both series were identified in a high-throughput screening (HTS) of the Sanofi compound collection and were not published previously. The inhibition of human NNMT (hNNMT) enzymatic activity by these compounds (IC_50_ (hNNMT) = 0.26 µM (**1**); 1.6 µM (**2**) and 0.18 µM (**3**)) was confirmed after re-synthesis. Therefore, we considered these series as good starting points for the further exploration of NNMT as a therapeutic target in different indications. All compounds act as competitive inhibitors either of nicotinamide alone (**2,3**) or of nicotinamide and SAM simultaneously (**1**), as will be further elucidated by our investigations. *K_m_* values for nicotinamide and SAM were reported as 400 and 1.8 µM, respectively [2].

## 2. Results and Discussion

### 2.1. Chemistry

**Scheme 1:**
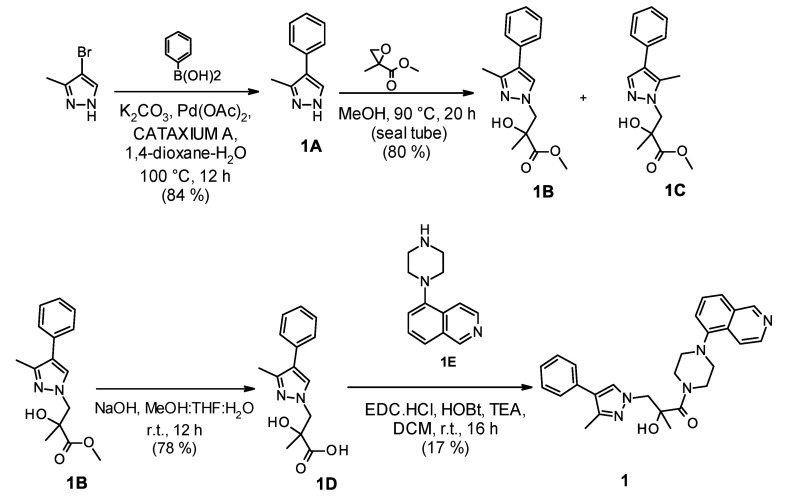
Synthesis of NNMT inhibitor (**1**).

Compound (**1**) was synthesized starting from 3-bromo-4-methyl pyrazole. Coupling of phenyl boronic acid with the pyrazole derivative in the presence of Pd catalyst yielded phenyl pyrazole (**1A**) in 84% yield. Thermal ring opening of the epoxide with phenyl-pyrazole afforded intermediates (**1B**) (minor isomer) and (**1C**) (major isomer) in 80% overall yield. Subsequent hydrolysis of the ester (**1B**) gave the acid (**1D**), and an amide coupling with the piperazine substituted isoquinoline (**1E**) resulted in the desired final product (**1**). Other derivatives of these series were synthesized following this general procedure (Scheme 1). All compounds of this series contain a chiral center and were profiled as racemic mixtures.

**Scheme 2:**
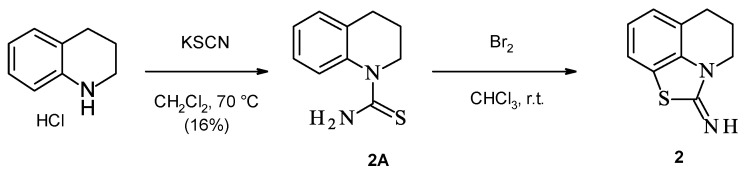
Synthesis of NNMT inhibitor (**2**).

Our hit structure (**2**) is accessible by two synthetic steps starting from tetrahydroquinoline: the thiourea (**2A**), generated from tetrahydroquinoline, undergoes a cyclization to the desired product (**2**) in the presence of bromine (Scheme 2).

**Scheme 3:**
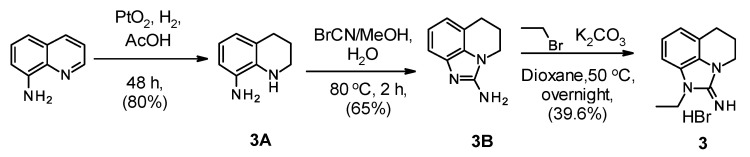
Synthesis of NNMT inhibitor (**3**).

The chemistry for the generation of lead (**3**) starts from 8-aminoquinoline, which is hydrogenated first in the pyridine part to give (**3A**). Afterwards, a cyclization to the five-membered heterocycle occurs with BrCN to give (**3B**) and a final alkylation with ethylbromide delivers the target compound (**3**) (Scheme 3).

**Scheme 4:**
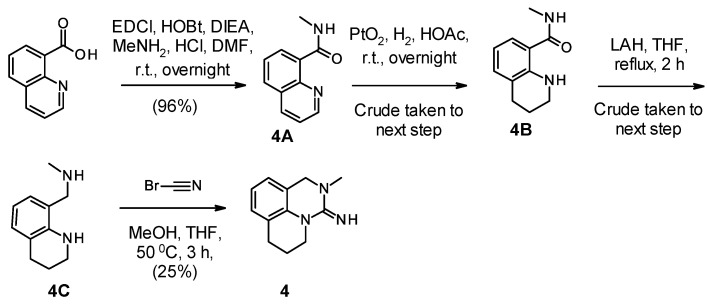
Synthesis of NNMT inhibitor (**4**).

Synthesis of compound (**4**) has been accomplished starting from quinoline-8-carboxylic acid. After formation of the corresponding N-methyl-carboxamide (**4A**), the pyridine ring of the quinoline system was reduced over PtO_2_ to give (**4B**), followed by reduction of the carboxamide with LAH to afford (**4C**). Treatment of the resulting diamine (**4C**) with BrCN results in a ring closure to the desired product (**4**) (Scheme 4).

### 2.2. Structural Biology

We crystallized hNNMT following a protocol from the Structural Genomics Consortium (SGC), which is available under DOI:10.2210/pdb2iip/pdb (see Section 3). As was described by Peng et al [25], NNMT possesses a class I AdoMet-dependent methyltransferase (MTase) core fold, comprising a central seven-stranded β-sheet, flanked on both sides by α-helices. In addition to the MTase core, there are two extra α-helices at the *N*-terminus, which, together with the β-hairpin Y203-S212, form a cap over the active site. Two more helices are inserted within W107-A134.

**Figure 3:**
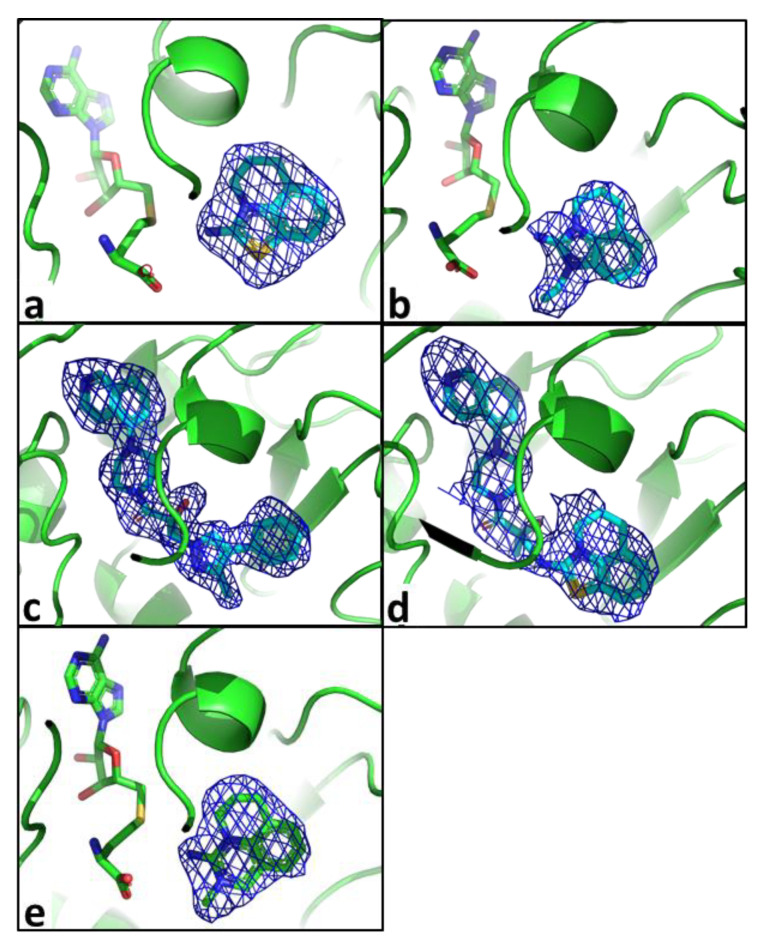
Omit maps, contoured at 2σ of **h**NNMT in complex with various inhibitors. (**a**) (**2**); 2.33 Å, (**b**) (**3**); 2.81 Å, (**c**) (**1**); 2.28 Å, (**d**) (**33**); 2.8 Å, (**e**) (**4**); 2.9 Å. Omit maps are calculated by subtracting calculated X-ray data, where the ligand has been omitted from the model, from the observed X-ray data. Anything that is present in the observed, but not in the calculated X-ray data (e.g., the omitted ligand) will show up as positive density in the resulting difference map. Since no information of the ligand has been used to calculate these maps, model bias will be minimal.

Figure 3a–e shows the observed electron densities observed with our inhibitors (**1**–**4**) and (**33**) after crystallization with hNNMT. In all cases, the electron density maps are sufficiently well defined to allow an unambiguous fitting of the corresponding inhibitor.

### 2.3. Binding Mode of the High Throughput Screening Hits

We started our co-crystallization efforts with the HTS hit (**2**). Co-crystallization experiments of hNNMT with (**2**) alone were not successful, but DSF experiments with (**2**) and the cofactor (reaction product) SAH indicated a strong additive stabilization and co-crystallization experiments with both SAH and (**2**) were successful.

**Figure 4:**
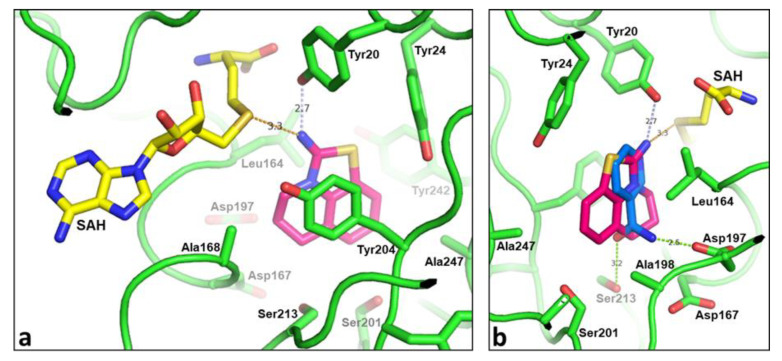
(**a**) Crystal structure showing the interactions of inhibitor (**2**) with hNNMT. Inhibitor (**2**) is shown in purple, *S*-adenosyl-homocysteine (SAH) in yellow, and hNNMT in green. (**b**) Comparison of the binding mode of nicotinamide (blue; pdb entry 3rod [4]) and inhibitor (**2**) (purple; this study), as well as a ribbon presentation of the NNMT protein from the inhibitor (**2**) complex (green).

The resulting crystal structure shows that inhibitor (**2**) is bound in a flat, rather hydrophobic cavity. Its “bottom” is formed by the side chains of Tyr204, Tyr24, and Ala247, while the “top” is formed by the side chains of Leu164, Ala168, Ala198, and Tyr242. Also present in the “top” is the carboxylate group of Asp197, which binds the amide nitrogen of the natural substrate nicotinamide. The “sides” of the cavity are more hydrophilic and contain the Oγ atoms of Ser201 and Ser213, as well as the hydroxyl group of Tyr20, which makes a hydrogen bond to the imine nitrogen of (**2**). In addition, the Cβ atom of Asp197 is present in the side of the cavity, while its carboxylate group points away from the cavity. An overview of these interactions is given in Figure 4.

As was mentioned above, the imine of (**2**) makes a hydrogen bond with the hydroxyl group of Tyr20. It also makes a van der Waals contact (d = 3.3 Å) with the sulfur of SAH. This is the position where the methyl group of the co-substrate *S*-adenosyl-l-methionine (SAM) would be transferred by the enzyme to the substrate nicotinamide.

As is shown in Figure 4b, inhibitor (**2**) binds in the binding pocket for the substrate nicotinamide [25]. The imine residue of inhibitor (**2**) is near the position of the pyridine nitrogen of nicotinamide, the nitrogen of which accepts a methyl group during normal catalytic turnover of the enzyme. The carboxamide group of nicotinamide interacts with the side chains of Asp197 and Ser213. Around this position, inhibitor (**2**) only has aliphatic and aromatic carbons. 

**Figure 5:**
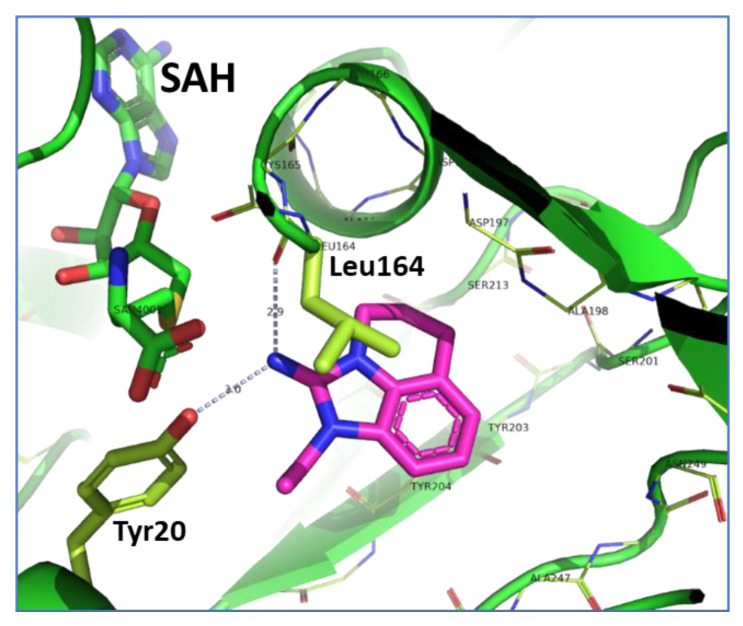
Crystal structure showing the interactions of (**3**) with hNNMT.

The crystal structure of the hNNMT complex with the HTS hit (**3**) (Figure 5) shows a very similar binding mode as for inhibitor (**2**). The NH group of inhibitor (**3**) forms a hydrogen bond with the side chain of Tyr20 and also with the main chain oxygen atom of Leu164, and there are van der Waals contacts of the inhibitor with the side chains of Ser201 and Ser213.

**Figure 6:**
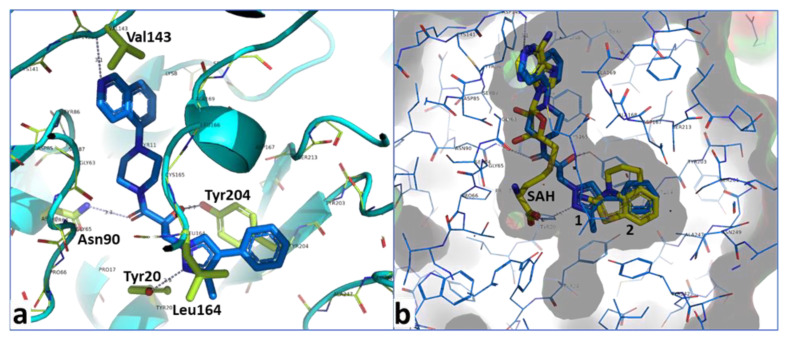
(**a**) Crystal structure showing the interactions of compound **1** (blue) with hNNMT. (**b**) Comparison with the binding mode of SAH (yellow), (**1**) (blue) and (**2**) (yellow) in the (**2**) complex. The surface of the protein is shown in grey. In the bottom-center, a large cavity is visible, below the Nam binding site.

Finally, the crystal structure of the complex of hNNMT with the HTS hit compound (**1**) shows that (**1**) occupies both the SAH and Nam binding pockets. As shown in Figure 3c, compound (**1**) fits well in the electron density maps and its binding mode is well defined. As will be discussed below, no SAH is bound to hNNMT since the SAH binding pocket is partially occupied by compound (**1**) (Figure 6b), making it impossible for NNMT to simultaneously bind SAH and the inhibitor.

The 3-methyl-4-phenyl-pyrazole group of inhibitor (**1**) is located in the nicotinamide binding pocket which is occupied by (**2**) in the hNNMT complex of inhibitor (**2**); inhibitor (**1**) occupies both the SAH- and the Nam binding pockets. As with other NNMT inhibitors, the methyl group of the 3-methyl-4-phenyl-pyrazole group points into a large cavity below the Nam binding site (see Figure 6b). With the exception of the piperazine nitrogens, all heteroatoms of the inhibitor are involved in hydrogen bonds with the hNNMT (Figure 6a).

### 2.4. Optimization of a Bisubstrate-Like Inhibitor Series Based on Compound ***1***

**Figure 7:**
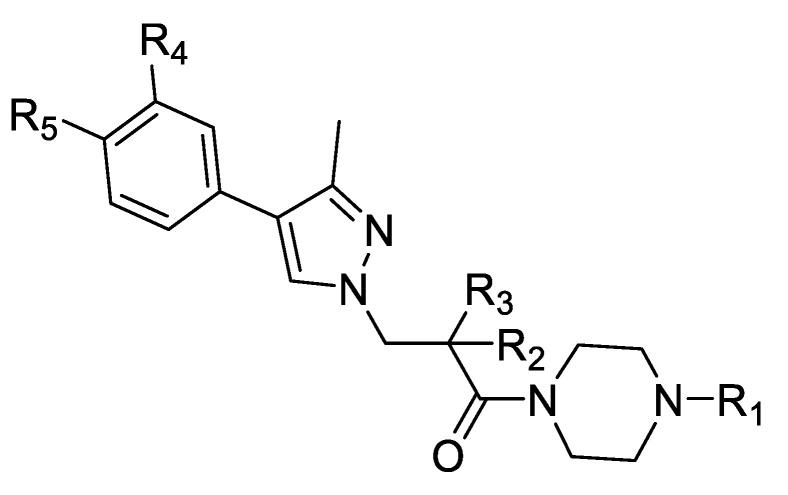
General formula of NNMT inhibitors described in Table 1 and Table 2.

To improve the potency and understand the mode of binding in compound (**1**), a focused library of compounds was synthesized, as shown in Figure 7 and Table 1. In (**6**), we replaced the hydroxyl and methyl group of the 2-hydroxy-2-methyl-propanoic carboxamide linker in (**1**) with hydrogen and found a six-fold reduction in potency on hNNMT and a 2.5-fold reduction in potency on mouse NMMT (mNNMT). In (**9**), only the R3 methyl group in the linker area was replaced with hydrogen and the result was a three-fold reduction in hNNMT potency (Table 1). Based on these observations, we decided to leave the linker region unchanged for further studies.

Substitution of the phenyl ring on the pyrazole moiety with fluoro in the meta position (**7**) delivered a two-fold increase in mNNMT enzymatic potency: however, difluoro substituted derivative (**10**) showed a mNNMT IC_50_ of >3 µM. The 6-substituted isoquinoline compound (**8**) completely lost hNNMT activity. Addition of a fluoro atom at the 6-position of the isoquinoline moiety (**11**) retains potency for both hNNMT and mNNMT. Interestingly, substitution of a methoxy as R_5_ in (**12**) resulted in a nanomolar potency for both hNNMT and mNMMT (Table 1). Even though these compounds showed reasonable potency for both hNNMT and mNNMT, their metabolic stability was insufficient for further profiling. We envisaged that one reason for the lack of metabolic stability is related to oxidation of the isoquinoline moiety. The N-oxide of isoquinoline (**13**) showed improved metabolic stability, but its potency for hNNMT and mNNMT was >3 µM.

**Figure 8:**
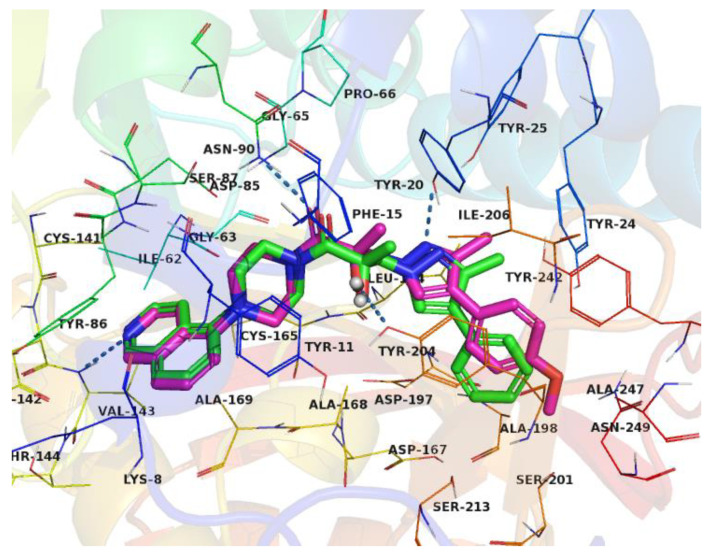
Predicted binding mode of compound **12** (magenta), along with the crystal structure of compound **1** (green), co-crystalized in hNNMT. The blue dashes are the polar interactions of compound **12** with the binding site amino acids. The figure was made using PyMOL v0.99.

**Figure 9:**
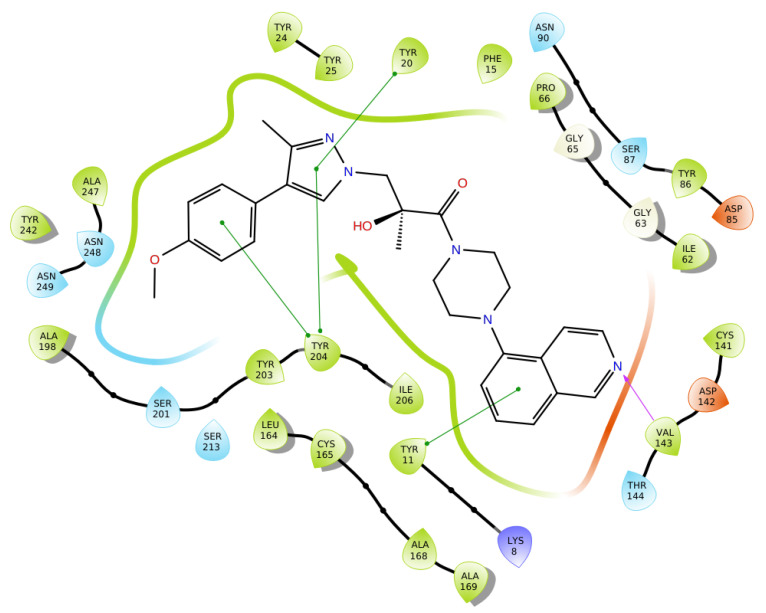
Ligand interaction diagram showing aromatic interactions of compound **12.** This figure was made using the Ligand Interaction Diagram module of Schrodinger/Maestro v2016-2.

Based on the crystal structure of hNNMT, co-crystallized with (**1**), we predicted the binding mode of (**12**) using the Glide docking module of the Schrodinger 2016-2 release [26] to guide further optimization. Derivative (**12**) binds by replacing the SAH: its isoquinoline group occupies the same position as the adenine of SAH and the piperazine residue acts as a replacement for the ribose moiety of SAH. The substituted pyrazole sub-unit of (**12**) binds into the nicotinamide pocket and is connected to the other part of the molecule by a 2-hydroxy-2-methyl-propionic carboxamide-based linker. Inhibitor (**12**) shows polar interactions with the backbone amino group of Val-143, the carbonyl after the piperazine demonstrates interactions with the side-chain of Asn-90, the hydroxyl group interacts with the hydroxyl side-chain of Tyr-204, and the pyrazole nitrogen interacts with the phenol-OH in Tyr-20. The ligand interaction diagram (Figure 8 and Figure 9) also shows aromatic interactions between the isoquinoline ring and Tyr-11, between the pyrazole ring and Tyr-20, and Tyr-204 engages both pyrazole and terminal phenyl rings. Compound (**12**) is more potent than the HTS hit (**1**), owing to a better interaction of its pyrazole group with the hydroxyl group of Tyr-20 and the additional hydrophobic contacts of its methoxy group with the surrounding residues such as Ala-247 and perhaps a water molecule bridging the methoxy and surrounding polar residues such as Ser-201 and Ser-213. Since the binding mode prediction uses the binding site of NNMT devoid of any explicit water molecules, such water molecule interactions cannot be visualized.

Based on this docking analysis, we came to the conclusion that the 4-(*para*-methoxy-phenyl)-pyrazol moiety present in (**12**) already delivered a very good fit into the nicotinamide binding pocket of NNMT and might not require additional optimization at this stage. After analysis of liver microsomes data of several close analogs in Table 1, metabolic stability clearly emerged as a very important parameter for further optimization. This observation was not completely unexpected, as alicyclic amines such as piperazines are well-established targets for metabolizing enzymes such as P450s or monoamine oxidases (MAOs). [27] In addition, metabolism on the isoquinoline ring has to be considered. Therefore, our strategy for further optimization focused on the identification of a metabolically stable replacement of the 5-piperazinyl-isoquinoline moiety binding into the SAH pocket. With such a replacement available to us, we expected a combination with the optimized pyrazole residue from (**12**) to deliver a new NNMT inhibitor suitable for in vivo investigations.

Our X-ray crystallographic data and docking studies guided us to keep the key nitrogen interactions with Val143 intact, while exploring different heteroaryl ring systems. Interestingly, quinolinone (**21**) showed reasonable potency, whereas the corresponding isoquinolinone derivative (**22**) was not active. However, the metabolic stability of compounds (**21**) and (**23**) did not improve. As can be seen from Figure 7 and Table 2, major structural changes in this region are not allowed. The activity difference between (**21**) and (**24**) also nicely confirms the general requirement of a heteroaromatic ring in this region of our NNMT inhibitor series.

In our next round of optimizations, we replaced the piperazine ring system with different heterocyclolakyl ring systems (Table 3). Replacement of piperazine with piperdine (**25**) led to a two-fold reduction in hNNMT and mNNMT potency compared to (**21**). Replacement with azetidine, pyrrolidine, or homopiperazine ring systems (**26**,**27**,**28**) resulted in a substantial loss of potency. Interestingly, (**30**) with a tertiary hydroxyl group on the piperidine ring was inactive whereas (**31**) with an isoquinoline ring showed a three-fold increase in potency. Even though (**31**) was active, the stability in liver microsomes was insufficient for further profiling. As already observed with compound (**13**), the corresponding N-oxide compound (**32**) showed improved metabolic stability for both humans and mice, but completely lost NNMT inhibition.

Taking all results presented so far into consideration, we did not identify a suitable replacement of the 5-piperazinyl-isoquinoline moiety featuring improved metabolic stability in combination with sufficient in vitro potency. Therefore, we had no clear pathway forward for further optimization of our highly active NNMT inhibitor (**12**), and we decided to focus our activities on different chemical series available to us.

### 2.5. Combination of Our Bisubstrate-Like Inhibitor Series with the HTS Leads ***2*** and ***3***

Based on the X-ray crystallographic data of our hits (**1**) and (**2**), the question arose as to whether the introduction of an appropriate 2-hydroxy-2-methyl-propionic carboxamide-based linker on (**2**) is able to transform our nicotinamide pocket binder into a true bisubstrate inhibitor.

**Scheme 5:**
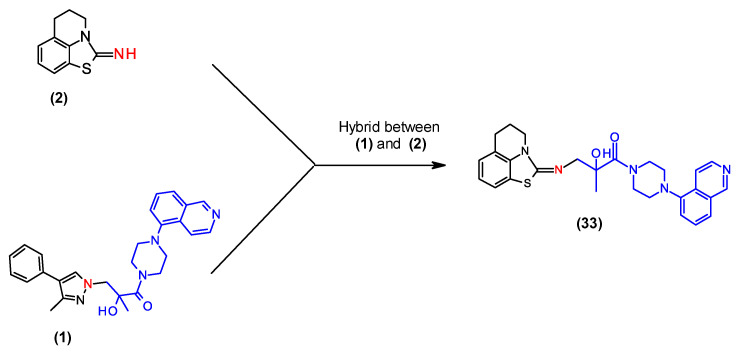
Rationale design of the bisubstrate inhibitor (**33**).

**Figure 10:**
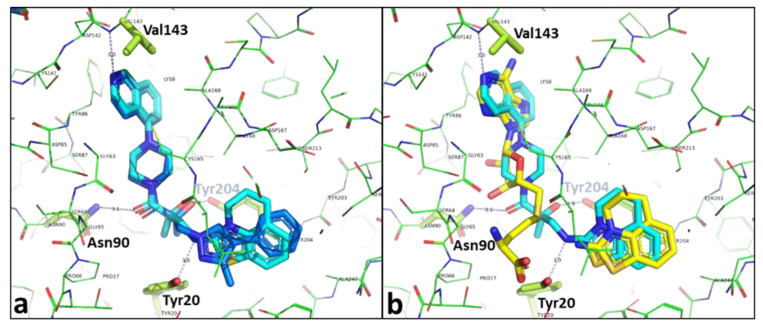
Crystal structure showing the binding mode of (**33**) (cyan) to hNNMT. (**a**) Comparison with the crystal structure of inhibitor (**1**) (blue). (**b**) Comparison with the crystal structure of the SAH and compound (**2**) complex (yellow).

In our case, we saw the synthetic feasibility of transferring the corresponding structural motif directly to the imino nitrogen of compound (**2**) delivering compound (**33**) in the process (Scheme 5). A subsequent X-ray analysis of (**33**) confirmed the binding mode as a bisubstrate inhibitor (Figure 10).

As shown in Figure 10a, the binding mode in the SAH pocket of the part that is common between (**33**) and (**1**) is identical. The position of (**2**) (Figure 10b) is slightly shifted with respect to the position of the tricyclic in (**33**), but also here the positions are very similar, showing that the fusion between the two inhibitor classes was successful.

Further profiling on human and mouse NNMT enzyme revealed that a bisubstrate binding motif does not necessarily translate into an improved inhibitory activity (Table 4). This may be related to the fact that a bisubstrate inhibitor has to compete with two substrates, SAM and Nam, which may be more difficult to achieve than when competing with a single substrate, especially when the second substrate, SAM, has a high affinity to the enzyme (*K_m_* = 1.8 µM, compared to 400 µM for Nam, [2]). We also decided against further modification of the linker as a viable optimization strategy. Based on our crystallographic data, we obtained the correct linker length allowing the important interactions with Val-143 and Tyr-204, and we also observed no obvious strains within the bound ligand. In addition, our results obtained with inhibitors (**6**) and (**9**) advised against any additional linker modifications.

### 2.6. Optimization of a Series of Tricyclic Compounds Binding to the Nam Pocket Only

In addition to the missing potency increase observed with the introduction of bisubstrate binding, we expected the 5-piperazinyl-isoquinoline residue in (**33**) to have comparable metabolic lability issues, as observed with the previous lead series around (**1**). Therefore, we decided to focus further studies on NNMT inhibitors binding into the Nam pocket of NNMT only.

**Scheme 6:**
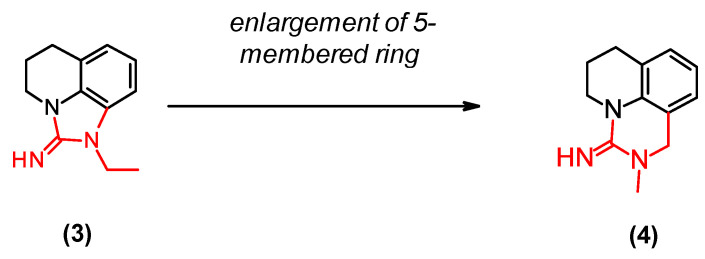
Rational design of inhibitor (**4**) based on X-ray data of inhibitor (**3**).

As a first strategy to maximize the space filling in this pocket, we went for ring enlargement of the five membered ring in (**3**) and synthesized (**4**) using a synthetic strategy outlined in Scheme 6.

**Figure 11:**
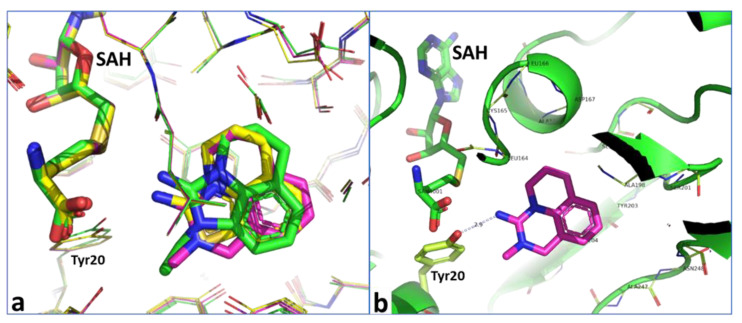
(**a**) Superposition of the crystal structures of the three Nam analogs (**2**) (yellow), (**3**) (green) and (**4**) (magenta). The binding mode of the three inhibitors is very similar. (**b**) Crystal structure showing the binding mode of (**4**) in hNNMT.

Our crystal structure showed that the binding of (**4**) is very similar to the binding of (**2**) and (**3**). The binding mode of (**4**) and an overlay of the binding modes of all three Nam binding analogs mentioned here is given in Figure 11.

Our ring enlargement strategy resulted in a clear improvement in in vitro potency, as can be seen in Table 5.

In addition, the metabolic lability of (**4**) was in an acceptable range from a drug discovery point of view and no CYP3A4 inhibition liabilities were detected. Based on these data, (**4**) became a starting point for additional Structure-Activity Realtionship (SAR) explorations and in vivo profiling studies, which will be discussed in more detail in subsequent publications.

## 3. Materials and Methods

### Crystallographic Studies 

Human NNMT with a His-tag and thrombin protease site, carrying the following mutations—K100A/E101A, E103A—was produced following a protocol from the Structural Genomics Consortium (SGC) (DOI:10.2210/pdb2iip/pdb).

However, in contrast to the published protocol, our NNMT could only be concentrated to ~0.8 mg/mL. Thermal shift measurements showed that addition of the cofactor SAH and the inhibitor A000268687A led to an increase in melting temperature of 7 °C, indicating that these ligands stabilized NNMT. We then concentrated NNMT in the presence of SAH and A000268687A and could reach a concentration of 6 mg/mL. This concentration was lower than the concentration published by the SGC (17.2 mg/mL), but it was sufficient for crystallization experiments. 

Human NNMT was crystallized using the following conditions: a protein solution with 6 mg/mL NNMT, 50 mM Tris/HCl, pH 8.0, 1 mM DTT, 86 µM *S*-adenosyl-l-homocysteine (SAH), 0.95 mM A000268687A and 5% *v/v* glycerol was equilibrated at room temperature in a hanging drop setup against 2.2 M ammonium sulfate with 0.1 M HEPES/Na, pH 7.6. Small crystals appeared after 1–2 weeks. Cocrystals of the other inhibitors were obtained using similar procedures.

The crystals were cryoprotected with 25% ethylene-glycol and flash frozen in liquid nitrogen. Data were collected at beamline PX-III of the Swiss Light source (SLS) in Villigen, Switzerland. Data were processed with the autoproc script by Clemens Vonrhein [28] from the global phasing consortium. The structure was solved by molecular replacement with Phaser [29] using a single monomer from pdb entry 2iip as a starting model. Refinement was done using Buster. [30] The final statistics are given in Table S1 of the supplement.

The space group is P1 and the cell-dimensions are around 46, 62, and 108 Å with α, β and γ around 92°, 98°, and 112°. There are four independent molecules in the asymmetric unit. This crystal form is the same as the published crystal form (pdb entry 2iip) of 46.0, 62.0, and 107.1 with α, β and γ 82.4°, 81.9°, and 68.3°, where the γ of entry 2iip corresponds to 180° − our γ. 

Our structures are identical within experimental error to the 2iip structure where the root-mean-square (r.m.s.) deviations of 0.2–0.3 Å between the independent molecules within our crystal are the same as the r.m.s. deviations with the four independent molecules of the 2iip structure. As shown in Figure 3, the inhibitors are generally well defined in the electron density maps and they could be fitted unambiguously. 

The structures have been deposited at the PDB under accession numbers 7BKG, 7BLE, 7NBJ, 7NBM, and 7NBQ.

## 4. Conclusions

We have identified the first non-adenosyl based small molecule bisubstrate inhibitor (**1**) of NNMT and additional NNMT modulators (**2**) and (**3**) utilizing the nicotinamide binding pocket alone. The binding mode of these inhibitors has been elucidated by X-ray crystallography and we used this information for the design of the novel inhibitors (**33**) and (**4**).

Our rational design towards the bisubstrate inhibitor (**33**) starting from the fragment-sized nicotinamide pocket binder (**2**) revealed no beneficial impact on in vitro inhibitory activity by addressing both binding sites of the NNMT enzyme simultaneously. In addition, our bisubstrate inhibitors posed a significant challenge for optimization towards acceptable metabolic stabilities while maintaining sufficient in vitro inhibitory potency.

As a consequence, we focused on the optimization of a chemical series binding only into the nicotinamide pocket of NNMT. By using detailed knowledge on the binding mode generated by several X-ray studies, we were able to identify (**4**), a small molecule NNMT inhibitor with a two-digit nano-molar potency on our target. Further studies on the SAR around (**4**) and characterization of the corresponding chemical series are in progress and will be published as a separate article.

## Figures and Tables

**Table 1:** Overview of NNMT inhibitors (**1**)–(**13**) and human/rat/mouse liver microsome (HLM/RLM/MLM) data.

Compound Number	R1	R2	R3	R4	R5	hNNMT IC_50_ [µM]	mNNMT IC_50_ [µM]	HLM/RLM/MLM % Remaining at 1 h
**1**	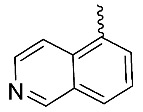	OH	Me	H	H	0.26	0.84	NA
**6**	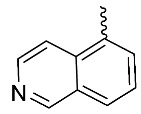	H	H	H	H	1.67	2.2	NA
**7**	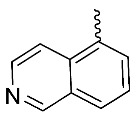	OH	Me	F	H	0.30	0.44	30/<5/<5
**8**	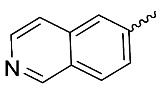	OH	Me	F	H	>10	NA	<5/<5/<5
**9**	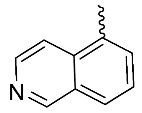	OH	H	F	H	0.88	2.43	NA
**10**	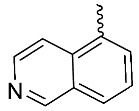	OH	Me	F	F	0.29	>3	7/NA/<5
**11**	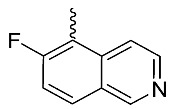	OH	Me	F	H	0.2	1.87	<5/NA/<5
**12**	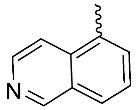	OH	Me	H	OMe	0.008	0.0008	NA
**13**	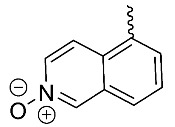	OH	Me	F	H	>3	NA	73/22/22

**Table 2:** Overview of NNMT inhibitors (**14**)–(**24**) and human/rat/mouse liver microsome (HLM/RLM/MLM) data.

Compound Number	R1	R2	R3	R4	R5	hNNMT IC_50_ [µM]	mNNMT IC_50_ [µM]	HLM/RLM/MLM % Remaining at 1 h
**14**	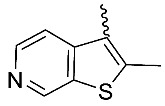	OH	Me	F	H	0.49	3.48	<5/NA/<5
**15**	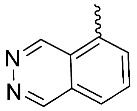	OH	Me	F	H	0.79	5.1	<5/<5/<5
**16**	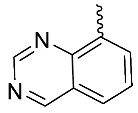	OH	Me	F	H	1.1	1.63	<5/<5/<5
**17**	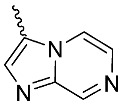	OH	Me	F	H	>3	NA	27/<5/8
**18**	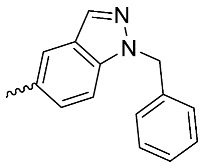	OH	Me	F	H	>30	NA	35/<5/30
**19**	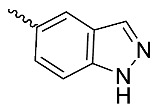	OH	Me	F	H	>10	NA	NA
**20**	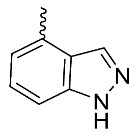	OH	Me	F	H	>3	NA	18/NA/<5
**21**	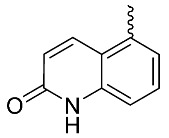	OH	Me	F	H	0.25	1.64	20/<5/<5
**22**	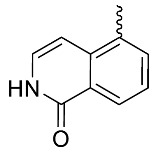	OH	Me	F	H	>30	NA	16/31/<5
**23**	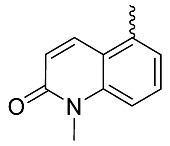	OH	Me	F	H	0.15	1.0	<5/NA/<5
**24**	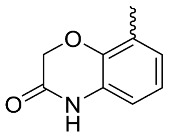	OH	Me	F	H	>10	NA	<5/NA/<5

**Table 3:** Overview of NNMT inhibitors (**25**)–(**32**) and human/rat/mouse liver microsome (HLM/RLM/MLM) data.

Compound Number	Structure	hNNMT IC_50_ [uM]	mNNMT IC_50_ [uM]	HLM/MLM
**25**	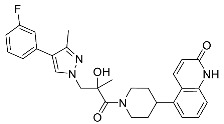	0.75	>3	17/<5
**26**	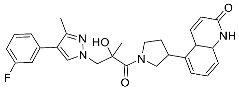	>10	NA	12/<5
**27**	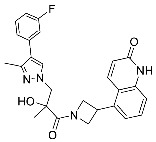	>30	NA	NA
**28**	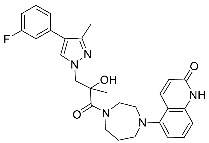	>3	>30	NA
**29**	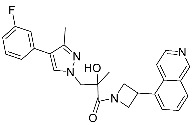	>30	NA	<5/<5
**30**	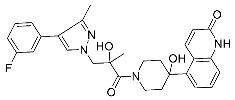	>10	NA	<5/<5
**31**	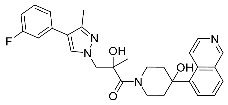	0.11	0.56	23/<5
**32**	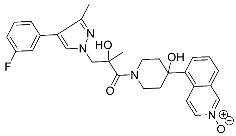	>30	NA	72/32

**Table 4:** hNNMT and mNNMT enzymatic activity of inhibitors (**2**) and (**33**).

Compound	hNNMT IC_50_ [µM]	mNNMT IC_50_ [µM]
(**2**)	1.3	2.3
(**33**)	2.5	3.0

**Table 5:** hNNMT and mNNMT enzymatic activity of inhibitors (**3**) and (**4**).

Compound	hNNMT IC_50_ [µM]	mNNMT IC_50_ [µM]
(**3**)	0.2	0.5
(**4**)	0.07	0.1

## Data Availability

Data is contained within the article or supplementary material.

## Supplementary Materials

The following are available online at, Table S1: Crystallographic Data and Refinement Statistics; Experimental Procedures S1: Synthesis of compounds (**1**) to (**33**); Experimental Procedures S2: Fluorescence-based NNMT assay; Experimental Procedures S3: Metabolic stability in human and mouse liver microsomes; Figure S1: High-resolution MS and NMR spectra of (**1**); Figure S2: High-resolution MS and NMR spectra of (**2**); Figure S3: High-resolution MS and NMR spectra of (**3**); Figure S4: High-resolution MS and NMR spectra of (**4**); Figure S5: High-resolution MS and NMR spectra of (**33**).

## Sample Availability

Samples of the compounds are not available from the authors.
