# Supplementary material for: Novel Inhibitors of Nicotinamide-N-Methyltransferase for the Treatment of Metabolic Disorders
Source: Molecules. 2021 Feb 13;26(4):991. doi: 10.3390/molecules26040991 (PMC7918612; doi:10.3390/molecules26040991)
Supplement: Supplementary file 1 [file molecules-26-00991-s001.pdf]

Supplementary Information to

Novel inhibitors of nicotinamide N-methyltransferase for the treatment of metabolic disorders

Aimo Kannt [1], Sridharan Rajagopal [2], Mahanandeesha S Hallur [2], Indu Swamy [2], Rajendra Kristam [2], Saravanakumar Dhakshinamoorthy [2], Joerg Czech [3], Gernot Zech [3], Herman Schreuder [3] and Sven Ruf [3,*]

[1] Fraunhofer Institute for Translational Medicine and Pharmacology ITMP, Theodor-Stern-Kai 7, 60596 Frankfurt am Main, Germany
[2] Jubilant Biosys Limited, #96, Industrial Suburb, 2nd Stage Yeshwanthpur, Bangalore-560022
[3] Sanofi-Aventis Deutschland Gmbh, Industriepark Hoechst, 65926 Frankfurt am Main, Germany

Content

Table S1. Crystallographic Data and Refinement Statistics

Experimental Procedures S1. Synthesis of compounds **(1)** to **(33)**

Experimental Procedures S2: Fluorescence-based NNMT enzymatic assay

Experimental Procedures S3: Metabolic stability in human and mouse liver microsomes

Figure S1: High-resolution MS and NMR spectra of **(1)**

Figure S2: High-resolution MS and NMR spectra of **(2)**

Figure S3: High-resolution MS and NMR spectra of **(3)**

Figure S4: High-resolution MS and NMR spectra of **(4)**

Figure S5: High-resolution MS and NMR spectra of **(33)**

Table S1: Crystallographic Data Collection and Refinement Statistics

inhibitor	**(2)**	**(3)**	**(1)**	**(33)**	**(4)**
pdb code	7BKG	7BLE	7NJB	7NMB	7NBQ
Data collection					
space group	P1	P1	P1	P1	P1
cell dimensions					
a,b,c (Å)	45.94 62.30 107.61	45.71 62.20 107.69	46.43 62.20 108.39	45.69 62.72 137.46	46.02 62.31 107.69
α,β,γ (°)	91.64 98.25 111.64	82.54 81.91 68.42	82.06 82.37 68.28	94.93 95.98 111.40	91.80 97.68 111.69
resolution (Å)	106.08 -2.33 (2.45-2.33)*	57.64 -2.81 (2.96-2.81)*	57.51 -2.28 (2.40-2.28)*	67.74 -3.33 (3.52-3.33)*	57.68 -2.55 (3.12-2.55)*
<I>/sig<I>	8.0 (2.4)	5.3 (3.2)	5.9 (2.4)	4.2 (2.8)	5.9 (2.9)
observed reflections	81698 (11385)	45690 (6309)	89205 (13052)	32912 (4780)	63137 (29178)
Rmeas (%)	0.084 (0.402)	0.115 (0.397)	0.100 (0.467)	0.417 (0.510)	0.154 (0.379)
completeness (%)	97.5 (97.1)	97.2 (95.9)	97.4 (96.2)	97.1 (96.7)	97.6 (97.2)
redundancy	1.8 (1.7)	1.8 (1.7)	1.8 (1.8)	1.7 (1.7)	1.8 (1.8)
Refinement					
protein atoms:	8225	8022	8130	8104	8100
inhibitor atoms:	52	60	136	140	60
water atoms:	613	274	656	265	487
other atoms:	104	104	6	0	104
resolution (Å)	57.69-2.326 (2.34-2.33)*	57.64-2.809 (2.83-2.81)*	57.51-2.275 (2.29-2.27)*	41.97-2.691 (2.72-2.69)*	106.54-2.479 (2.50-2.48)*
Rfactor (%)	17.8 (20.0)	19.1 (20.9)	17.1 (20.1)	21.4 (28.3)	19.5 (21.9)
Rwork (%)	17.5 (19.5)	18.6 (20.0)	16.9 (19.7)	20.9 (28.2)	19.1 (21.4)
Rfree (%)	23.6 (32.0)	27.0 (38.8)	22.0 (27.6)	31.4 (32.5)	27.1 (29.1)
average Bfactors (Å²):					
protein:	32.82	31.14	30.43	48.52	34.15
inhibitor:	27.95	16.52	21.14	38.80	21.76
water:	40.41	23.86	42.00	36.96	36.49
other:	24.37	19.73	69.14		24.14
rmsd bond lengths (Å)	0.008	0.008	0.008	0.008	0.008
rmsd bond angles (°)	1.00	1.04	1.00	1.06	1.02

* the highest resolution bin is given in brackets

Experimental Procedures S1

Synthesis of compounds (1)-(33)

Synthesis of 2-hydroxy-1-(4-(isoquinolin-5-yl)piperazin-1-yl)-2-methyl-3-(3-methyl-4-phenyl-1H-pyrazol-1-yl)propan-1-one (1)

3-Methyl-4-phenyl-1H-pyrazole (1A)

To a solution of 4-bromo-3-methyl-1H-pyrazole (5.0 g, 31 mmol) in 1,4-dioxane (60.0 mL) and H_2O (20.0 mL) were added (3-phenyl)boronic acid (4.7 g, 38.8 mmol), potassium carbonate (12.85 g, 93.0 mmol), CatacXium A (1.04g, 1.55 mmol) and palladium acetate (0.11g, 0.31 mmol) under N_2 atmosphere. Then the reaction mixture was heated at 100 °C for 12 h. After completion of the starting material, the reaction mixture was cooled to room temperature, diluted with ethyl acetate, filtered through celite and the organic layer was separated. The organic layer was washed with water and brine solution, dried over sodium sulphate, filtered and concentrated under reduced pressure to get the crude compound. The obtained crude was purified by flash chromatography and the product was eluted with 2 % methanol in dichloromethane. Pure fractions were combined and evaporated to afford to afford the desired product **1A** as white solid (4.1 g, 84 %). MS (ESI^+) *m/z* 159 $(M+H)^+$.

Methyl 2-hydroxy-2-methyl-3-(3-methyl-4-phenyl-1H-pyrazol-1-yl)propanoate (1B and 1C)

To a solution of 4-(phenyl)-3-methyl-1H-pyrazole (**1A**, 0.7 g, 4.42 mmol) in methanol (10.0 mL) was added methyl 2-methyloxirane-2-carboxylate (1.02 g, 8.84 mmol) and the mixture was stirred at 90 °C for 20 h in a sealed tube. After completion of the starting material the reaction mixture was cooled to room temperature and concentrated under reduced pressure to get the crude residue containing both isomers. Further the two isomers were separated by flash chromatography with 15 % ethylacetate in hexane as eluent to afford methyl 2-hydroxy-2-methyl-3-(3-methyl-4-phenyl-1H-pyrazol-1-yl)propanoate (**1B**) as off-white solid (0.22 g, 18 %) and methyl 2-hydroxy-2-methyl-3-(5-methyl-4-phenyl-1H-pyrazol-1-yl)propanoate (**1C**, 0.74 g, 61 %). MS (ESI^+) *m/z* 275.1 $(M+H)^+$.

2-Hydroxy-2-methyl-3-(3-methyl-4-phenyl-1H-pyrazol-1-yl)propanoic acid (1D)

To a solution of methyl 2-hydroxy-2-methyl-3-(3-methyl-4-phenyl-1H-pyrazol-1-yl)propanoate (**1B**, 0.74 g, 2.69 mmol) in methanol (5 mL), tetrahydrofuran (5 mL) and water (5mL) was added sodium hydroxide (0.539 g, 13.4 mmol) and the solution was stirred at room temperature for 1 h. After completion of starting material the reaction mixture was concentrated under reduced pressure, diluted with water and acidified with 1N HCl aqueous solution to pH~3. The product was extracted with dichloromethane, washed with brine solution and dried over sodium sulphate, filtered and concentrated under reduced pressure to get desired product as off-yellow solid (**1D**, 0.55 g, 78 %). MS (ESI^+) *m/z* 261.1 $(M+H)^+$.

2-Hydroxy-1-(4-(isoquinolin-5-yl)piperazin-1-yl)-2-methyl-3-(3-methyl-4-phenyl-1H-pyrazol-1-yl)propan-1-one (1)

To a solution 2-hydroxy-2-methyl-3-(3-methyl-4-phenyl-1H-pyrazol-1-yl)propanoic acid (**1D**, 0.1 g, 0.384 mmol) in dichloromethane (10.0 mL) were added triethylamine (0.16 mL, 1.15 mmol), 5-(piperazin-1-yl)isoquinoline hydrochloride (0.115 g, 0.461 mmol), EDC.HCl (0.184 g, 0.192 mmol) and HOBt (0.029 g, 0.192 mmol), then the reaction mixture was stirred at room temperature for 16 h. After completion of starting material the reaction mixture was quenched with ice water (10.0 mL) and extracted with DCM. The combined organic layer was washed with saturated $NaHCO_3$ solution and brine solution, dried over anhydrous sodium sulphate, filtered and concentrated under reduced pressure to give crude residue which was purified by flash chromatography using 5 % methanol in dichloromethane as eluent to afford the desired product compound **1** as white solid (0.035 g, 17 %).^{1}H

NMR (500 MHz, DMSO-d_6): δ 9.65 (br s, 1 H), 8.61 (br s, 1H), 8.31 (br d, J = 5.8 Hz, 1H), 8.08 (d, *J* = 8.2 Hz, 1H), 7.91 (s, 1H), 7.80 (t, *J* = 7.8, 7.8 Hz, 1H), 7.62 (d, *J* = 7.6 Hz, 1H), 7.48 – 7.41 (m, 4H), 7.23 (m, 1H), 6.01 (br s, 1H), 4.38 (d, *J* = 15.1 Hz, 1H), 4.34 (d, *J* = 15.1 Hz, 1H), 4.24 (m, 2H), 3.82 (m, 2H), 3.07 (br, 4H), 2.31 (s, 3H), 1.35 (s, 3H); ^{13}C NMR (126 MHz, DMSO-d_6): δ 171.7, 149.8, 148.3, 147.3, 143.8, 136.2, 133.5, 132.4, 130.4. 129.7, 128.7, 125.8, 123.9, 122.3, 119.6, 119.1, 75.6, 59.2, 58.5, 52.5, 24.0, 13.3; MS (ESI$^+$) *m/z* 456.2 (M+H)$^+$; HPLC Purity @ 260 nm, 99.2 %. HRMS (ESI$^+$): *m/z* calcd for $C_{27}H_{29}N_5O_2$ (M+H)$^+$: 456.2394; Found: 456.2388.

Synthesis of 3-thia-1-azatricyclo[6.3.1.04,12]dodeca-4(12),5,7-trien-2-imine (2)

3,4-Dihydro-2H-quinoline-1-carbothioamide (2A)

1,2,3,4-tetrahydroquinoline A (40 g, 300.8 mmol) is neutralized with HCl solution and added to an aqueous solution of potassium thiocyanate (146 g, 1.50 mmol). The mixture was stirred at 70 °C overnight. The mixture was extracted with CH_2Cl_2 (200 mL*3). The organic layer was washed with brine, dried over Na_2SO_4, filtered and concentrated. The crude product was purified by column chromatography to afford compound **2A** (7.5 g, 16%) as yellow solid.

3-Thia-1-azatricyclo[6.3.1.04,12]dodeca-4(12),5,7-trien-2-imine (2)

Compound **2A** (12 g, 62.5 mmol) was dissolved in 100 mL $CHCl_3$ and Br_2 (12 g, 75 mmol) in 25 mL $CHCl_3$ was added in portions at room temperature. After completion of the bromination (6h), the solvent is removed by evaporation and the residue is recrystallized twice with ethyl acetate to afford the product **2** as HBr salt. (7,7g, 65%). ^{1}H NMR (600 MHz, DMSO-d_6): δ 9.98 (br s, 2H), 7.80 (dd, *J* = 7.4, 1.3 Hz, 1H), 7.37 – 7.33 (m, 2H), 4.14 (t, *J* = 6.0 Hz) 2H), 2.91 (t, *J* = 6.0 Hz, 2H), 2.15 (dq, *J* = 6.1, 5.9 Hz, 2H). ^{13}C NMR (151 MHz, DMSO-d_6) δ 166.7, 135.0, 126.2, 125.5, 124.7, 121.7, 120.9, 44.7, 23.4, 20.2. HRMS (ESI$^+$): calcd for $C_{10}H_{10}N_2S$ (M+H)$^+$: 191.0637; Found: 191.0641.

Synthesis of 3-ethyl-1,3-diazatricyclo[6.3.1.04,12]dodeca-4(12),5,7-trien-2-imine (3)

1,2,3,4-Tetrahydroquinolin-8-amine (3A)

A mixture of 8-aminoquinoline (40.0 g, 278 mmol) and PtO_2 (1.26 g, 2.6 mmol) in glacial acetic acid (300 mL) was stirred under a hydrogen pressure of 4 MPa for 48 h at room temperature. The solvent was removed in vacuo at 40 °C to give the crude product as red oil, and then DCM (400 mL) and a saturated aqueous solution of $NaHCO_3$ (600 mL) were added to the crude residue. The obtained solution was extracted with DCM (3 * 300 mL). The combined organic layers were washed with H_2O (3 * 300 mL) and dried over Na_2SO_4. The solvent was evaporated under reduced pressure at 40 °C. The residue was purified by flash chromatography on silicagel using 11 % ethyl acetate in petrol ether as eluent to afford 1,2,3,4-tetrahydroquinolin-8-amine (**3A**, 33 g, 80 %).

1,3-Diazatricyclo[6.3.1.04,12]dodeca-2,4(12),5,7-tetraen-2-amine (3B)

To a solution of compound **3A** (33 g, 223 mmol) in MeOH/H_2O (1/1, 330 mL) was added cyanogen bromide (47 g, 446 mmol). The reaction mixture was stirred at 50 °C for 3 h, then MeOH was removed in vacuo. The aqueous residue was basified to pH = 8 by 1N aq. NaOH and extracted with DCM/MeOH (10:1). The organic layer was washed with brine and dried over Na_2SO_4. The solvent was evaporated under reduced pressure at 40 °C. The residue was purified by flash chromatography on silicagel using 9 % MeOH in DCM as eluent to afford compound **3B** as brown solid (25 g, 65 %).

3-Ethyl-1,3-diazatricyclo[6.3.1.04,12]dodeca-4(12),5,7-trien-2-imine hydrobromide (3)

To a solution of compound **3B** (25 g, 144 mmol) in dry dioxane (500 mL) was added bromoethane (17.3 g, 158mmol) and K_2CO_3 (39.8g, 316mmol) The reaction mixture was stirred overnight vigorously at 80 °C. After completion of the reaction, the mixture was filtered to separate the solid K_2CO_3, and the organic solvent was evaporated. The residue was then crystallized from ethanol to give the desired

product as HBr salt (**3,** 11.5 g, 40 %). ^{1}H NMR (600 MHz, DMSO-d_6): δ 9.01 (s, 2H), 7.38 (d, *J* = 8.0 Hz, 1H), 7.21 (t, *J* = 7.8 Hz, 1H), 7.10 (d, *J* = 7.4 Hz, 1H), 4.24 (q, *J* = 7.2 Hz, 2H), 4.07 (t, *J* = 5.8 Hz, 2H), 2.88 (t, *J* = 6.0 Hz, 2H), 2.14 (dq, *J* = 6.1, 5.8 Hz, 2H), 1.28 (t, *J* = 7.2 Hz, 3H); ^{13}C NMR (151 MHz, DMSO-d_6): δ 148.1, 127.8, 126.7, 123.1, 122.2, 121.2, 107.7, 41.1, 37.8, 22.4, 21.1, 13.2; HRMS (ESI$^+$): calcd for $C_{12}H_{15}N_3$ $(M+H)^+$: 202.1339; Found: 202.1340.

Synthesis of 2-methyl-1,2,6,7-tetrahydro-5H-pyrido[3,2,1-ij]quinazolin-3-ylideneamine (4)

N-Methylquinoline-8-carboxamide (4A)

The mixture of quinoline-8-carboxylic acid (2 g,12 mmol), EDCI (2.2 g, 12 mmol), HOBt (1.6 g, 12 mmol), DIEA (1.5 g, 12 mmol) and methylamine hydrochloride (0.77 g, 12 mmol) was stirred at RT in DCM (50 mL) overnight. The reaction was washed with water (50 mL x 3), dried, concentrated to afford desired product (**4A**, 2 g, 96 % yield). ^{1}H NMR (400 MHz, $CDCl_3$) : δ 11.21 (s, 1H), 8.98 – 8.91 (m, 1H), 8.87 (d, *J* = 7.4 Hz, 1H), 8.30 (d, *J* = 8.3 Hz, 1H), 7.97 (d, *J* = 8.1 Hz, 1H), 7.68 (t, *J* = 7.7 Hz, 1H), 7.51 (dd, *J* = 8.2, 4.2 Hz, 1H), 2.81 (s, 3H).

N-Methyl-1,2,3,4-tetrahydroquinoline-8-carboxamide (4B)

To the mixture of **4A** (2 g, 10.75 mmol) in HOAc (20 mL) was added PtO_2 (469 mg, 2.1 mmol) at room temperature and the suspension was stirred under a hydrogen atmosphere of 0.1 MPa at room temperature overnight. LCMS showed that the desired product was formed. The mixture was filtered and the filtrate was concentrated under reduced pressure to give the crude product **4B** (2.1 g) as clear oil. The material was used in next step without further purification.

N-Methyl-1-(1,2,3,4-tetrahydroquinolin-8-yl)methanamine (4C)

To **4B** (0.5 g, 2.6 mmol) in THF (50 mL) at RT was added LAH (0.3 g, 7.9 mmol) and the reaction mixture was stirred at reflux for 3 h. The mixture was quenched with NH3.H2O (20 mL), filtered and concentrated under reduced pressure. The residue obtained (**4C**, 0.5 g, crude) was used in the next step without further purification.

2-Methyl-1,2,6,7-tetrahydro-5H-pyrido[3,2,1-ij]quinazolin-3-ylideneamine hydrobromide (4)

A mixture of **4C** (0.665 g, 3.78 mmol) and BrCN (400 mg, 3.78 mmol) in MeOH (20 mL)/water (2 mL) was stirred at 50 ^{0}C for 3 h. The reaction mixture was concentrated and the residue thus obtained was washed with ether (50 mL) to afford a white solid as HBr salt of the product (**4**, 0.18 g, 25% yield). ^{1}H NMR (500 MHz, DMSO-d_6): δ 8.06 (s, 2 H), 7.14 (d, *J* = 7.0 Hz, 1H), 7.11 - 7.06 (m, 2H), 4.48 (s, 2H), 3.73 (t, *J* = 5.9 Hz, 2H), 3.14 (s, 3H), 2.74 (t, *J* = 6.0 Hz, 2H), 1.98 (dq, *J* =6.0, 5.9 Hz, 2H); ^{13}C NMR (126 MHz, DMSO-d_6) δ 152.7, 131.5, 128.4, 126.0, 123.8, 123.6, 120.0, 49.0, 45.1, 37.8, 25.5, 20.8; HRMS (ESI$^+$): calcd for $C_{12}H_{15}N_3$ $(M+H)^+$: 202.1339; Found: 202.1339.

Synthesis of 1-(4-(isoquinolin-5-yl)piperazin-1-yl)-3-(3-methyl-4-phenyl-1H-pyrazol-1-yl)propan-1-one (6)

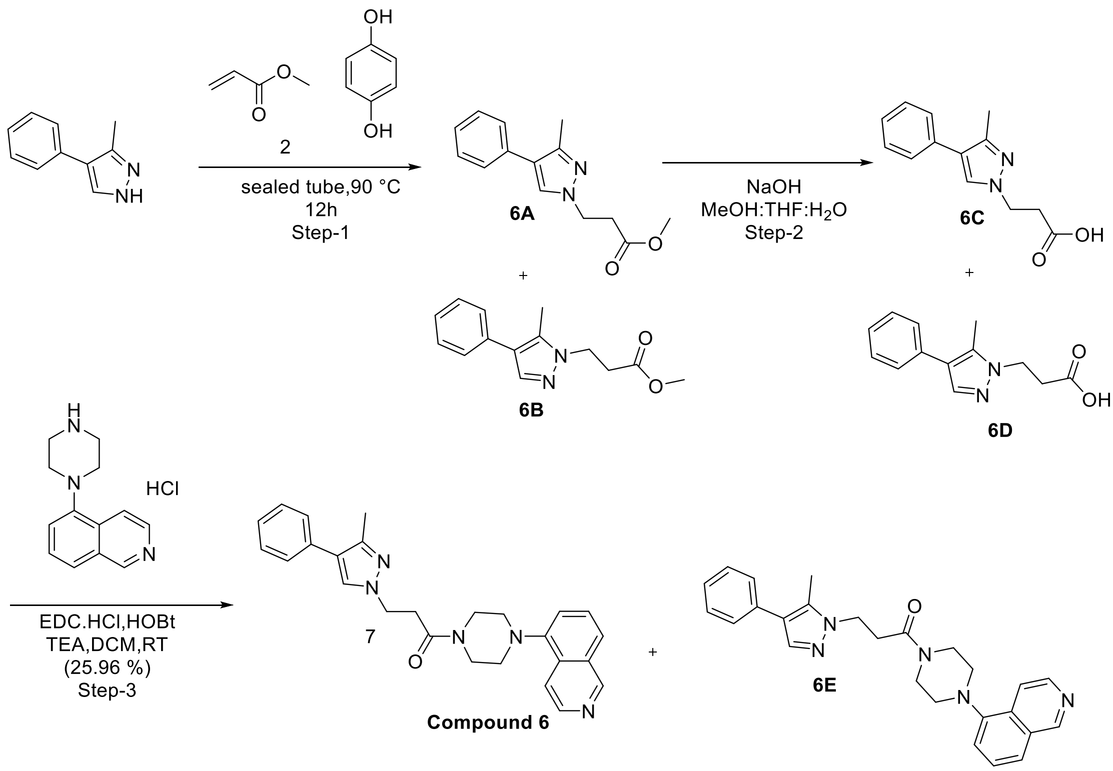

Methyl 3-(3-methyl-4-phenyl-1H-pyrazol-1-yl)propanoate and methyl 3-(5-methyl-4-phenyl-1H-pyrazol-1-yl)propanoate (6A and 6B)

A mixture of 3-methyl-4-phenyl-1H-pyrazole (0.5 g, 3.1605 mmol), methyl acrylate (0.679 g, 7.9013 mmol) and hydroquinone (0.034 g, 0.3160 mmol) was heated to 90 °C for 16.0 h in a sealed tube. After completion of starting material the reaction mixture was quenched with water (60 mL) and the product was extracted with dichloromethane (3 x 50 mL). The combined organic layer was dried over anhydrous sodium sulphate, filtered and concentrated under reduced pressure. The crude residue was purified by flash chromatography using 25 % ethyl acetate in hexane to afford a mixture of methyl 3-(3-methyl-4-phenyl-1H-pyrazol-1-yl)propanoate and methyl 3-(5-methyl-4-phenyl-1H-pyrazol-1-yl)propanoate (**6A** and **6B**) as a brown sticky material (0.81 g, crude). MS (ESI$^+$) *m/z* 245.1 $(M+H)^+$.

3-(3-Methyl-4-phenyl-1H-pyrazol-1-yl)propanoic acid and 3-(5-methyl-4-phenyl-1H-pyrazol-1-yl)propanoic acid (6C and 6D)

To a mixture of methyl 3-(3-methyl-4-phenyl-1H-pyrazol-1-yl)propanoate and methyl 3-(5-methyl-4-phenyl-1H-pyrazol-1-yl)propanoate (**6A** and **6B**, 0.81 g, 3.31 mmol) in 1:1:1 of MeOH:THF:H_2O (10:10:10 mL) was added sodium hydroxide (0.273 g, 6.845 mmol) and the mixture was stirred at room temperature for 1 h. After completion of starting material the reaction mixture was concentrated under reduced pressure. The crude residue was dissolved in water (10 mL), washed with ether and the aqueous layer was acidified with 4N HCl. The product was extracted with DCM, dried over anhydrous sodium sulphate, filtered and concentrated under reduced pressure to afford a mixture of **6C** and **6D** as a brown sticky solid (0.67 g, 89 %). MS (ESI$^+$) *m/z* 231.1 $(M+H)^+$.

1-(4-(Isoquinolin-5-yl)piperazin-1-yl)-3-(3-methyl-4-phenyl -1H-pyrazol-1-yl)propan-1-one (6)

To a stirred solution of 3-(3-methyl-4-phenyl-1H-pyrazol-1-l)propanoic acid and 3-(5-methyl-4-phenyl-1H-pyrazol-1-yl)propanoic acid (**6C** and **6D**, 0.2 g, 1.0780 mmol) in dichloromethane (25 mL) was added triethylamine (0.28 mL, 2.605 mmol), 1-ethyl-3-(3-dimethylaminopropyl)carbodiimide (0.414 g, 2.1712 mmol) and hydroxybenzotriazole (0.066 g, 0.4342 mmol) under nitrogen atmosphere. The reaction mixture was stirred at room temperature for 16.0 h. After completion of starting material the reaction mixture was quenched with water (60 mL). The phases were separated and the product was further extracted to dichloromethane. The combined organic layer was dried over anhydrous sodium sulphate, filtered and concentrated under reduced pressure. The crude residue was purified by flash chromatography using 4 % methanol in dichloromethane as an eluent followed by preparative HPLC purification (Column: CHIRALPAK IA(250 mm X 4.6mm X 5μm), Mobile phase: n-Hexane : 0.1 % DEA in Ethanol (50:50), Flow rate: 1.0 mL/min). The appropriate fractions were collected and concentrated under reduced pressure to afford 1-(4-(isoquinolin-5-yl)piperazin-1-yl)-3-(3-methyl-4-phenyl-1H-pyrazol-1-yl)propan-1-one as an off-white solid (**6**, 0.1 g, 26 %).

^{1}HNMR (400 MHz, DMSO-d_6): δ 9.26 (s, 1H), 8.50 (d, *J* = 4.0 Hz, 1H), 7.92 (s, 2H), 7.78 (d, *J* = 8.4 Hz, 1H), 7.55 (t, *J* = 7.6 Hz, 1H), 7.41 – 7.36 (m, 4H), 7.27 (d, *J* = 6.8 Hz, 1H), 7.20 – 7.19 (m, 1H), 4.31 (t, *J* = 6.4 Hz, 2H), 3.73 – 3.70 (m, 4H), 2.95-296 (m, 6H), 2.28 (s, 3H). MS (ESI^{+}) *m/z* 426.2 (M+H)$^{+}$. HPLC purity: 99.1 %.

The following compounds (**7**,**8**) were synthesized using the procedure described for compound **1**:

3-(4-(3-Fluorophenyl)-3-methyl-1H-pyrazol-1-yl)-2-hydroxy-1-(4-(iso-quinolin-5-yl)piperazin-1-yl)-2-methylpropan-1-one (7)

Yield 26 %. ^{1}HNMR (400 MHz, DMSO-d_6): δ 9.26 (s, 1H), 8.49 (d, *J* = 5.6 Hz, 1H), 7.94 (m, 2H), 7.78 (d, *J* = 8.4 Hz, 1H), 7.55 (t, *J* = 7.6 Hz, 1H), 7.41 – 7.39 (m, 1H), 7.31 (d, *J* = 7.6 Hz, 1H), 7.27 – 7.25 (m, 2H), 7.03 – 7.01 (m, 1H), 5.94 (s, 1H), 4.32 (s, 2H), 4.16 (br s, 2H), 3.80 (br s, 2H), 3.02 (s, 4H), 2.31 (s, 3H), 1.33 (s, 3H). MS (ESI^{+}) *m/z* 474.2 (M+H)$^{+}$. HPLC purity: 98.5 %.

3-(4-(3-Fluorophenyl)-3-methyl-1H-pyrazol-1-yl)-2-hydroxy-1-(4-(isoquinolin-6-yl)piperazin-1-yl)-2-methylpropan-1-one (8)

Yield 19 %. ^{1}HNMR (400 MHz, DMSO-d_6): δ 8.63 (d, *J* = 3.2, 1H), 8.10 (d, *J* = 8.0 Hz, 1H), 7.90 (s, 1H), 7.83 (d, *J* = 12.0 Hz, 1H), 7.62 – 7.59 (m, 1H), 7.41 – 7.37 (m, 2H), 7.24 – 7.17 (m, 3H), 7.01 (t, *J* = 8.4 Hz, 1H), 5.94 (s, 1H), 4.33 (s, 1H), 4.05 (br s, 2H), 3.69 (br s, 2H), 3.36 (s, 4H), 2.30 (s, 3H), 1.32 (s, 3H). MS (ESI^{+}) *m/z* 474.2 (M+H)$^{+}$. HPLC purity: 99.2 %.

Synthesis of 3-(4-(3-fluorophenyl)-3-methyl-1H-pyrazol-1-yl)-2-hydroxy-1-(4-(isoquinolin-5-yl)piperazin-1-yl)propan-1-one 2,2,2-trifluoroacetate (9)

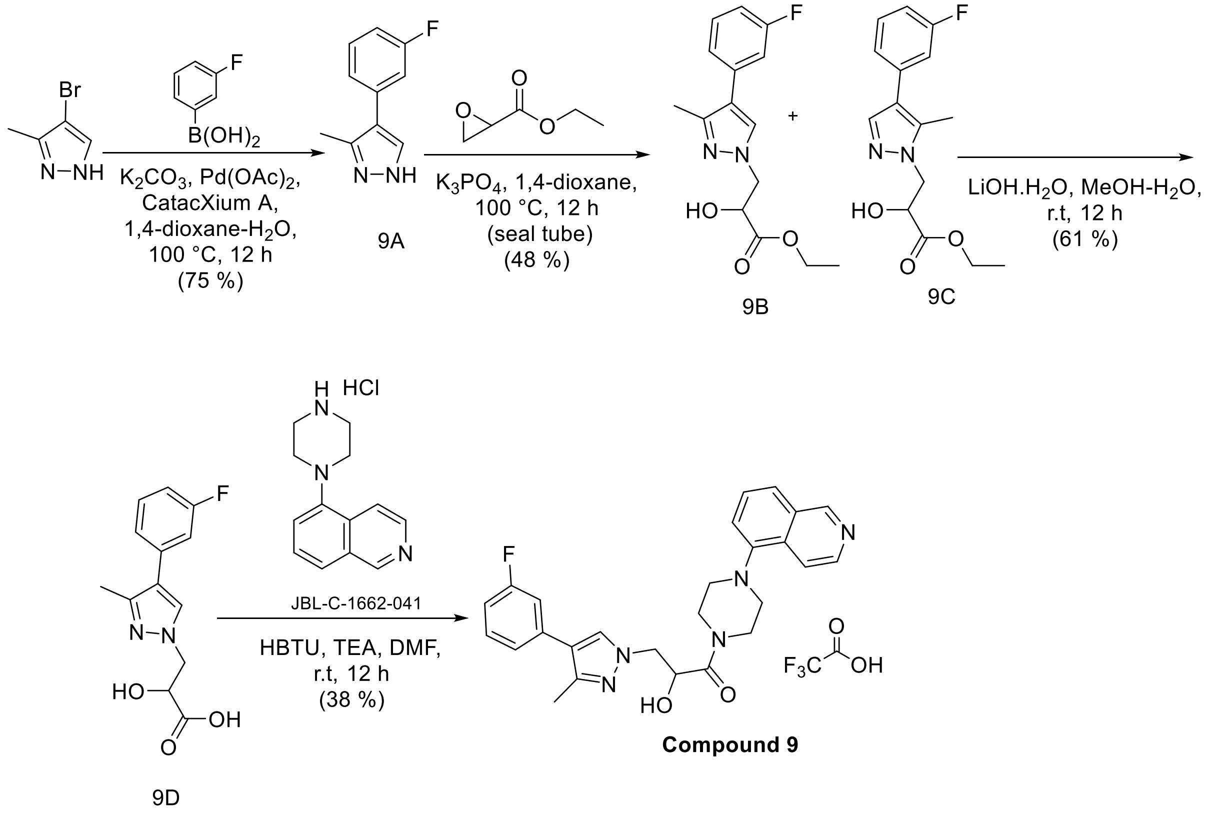

Compound 9B seprated through flash chromatography and confirmed by NOE and procced to next step

4-(3-Fluorophenyl)-3-methyl-1H-pyrazole (9A)

To a solution of 4-bromo-3-methyl-1H-pyrazole (5.0 g, 31 mmol) in 1,4-dioxane (60.0 mL) and H_2O (20.0 mL) were added (3-F-phenyl)boronic acid (4.7 g, 38.8 mmol), potassium carbonate (12.85 g, 93.0 mmol), CatacXium A (1.04 g, 1.55 mmol) and palladium acetate (0.11g, 0.31 mmol) under N_2 atmosphere. The reaction mixture was heated at 100 °C for 12 h. After completion of the starting material, the reaction mixture was cooled to room temperature, diluted with ethyl acetate, filtered through celite and the organic layer was separated. The organic layer was washed with water and brine solution, dried over sodium sulphate, filtered and concentrated under reduced pressure to get the crude product which was purified by flash chromatography by eluting with 2 % methanol in dichloromethane. The pure fractions were combined and evaporated to afford the desired product as white solid (**9A**, 4.1 g, 75 %). ^1H NMR (400 MHz, DMSO-d_6): δ 12.69 - 12.62 (m, 1H), 7.99 - 7.71 (m, 1H), 7.41 -7.36 (m, 1H), 7.28 - 7.22 (m, 2H), 7.03 - 6.98 (m, 1H), 2.37 (s, 3H). MS (ESI$^+$) *m/z* 173 $(M+H)^+$.

Ethyl 3-(4-(3-fluorophenyl)-3-methyl-1H-pyrazol-1-yl)-2-hydroxy-propanoate and ethyl 3-(4-(3-fluorophenyl)-5-methyl-1H-pyrazol-1-yl)-2-hydroxy-propanoate (9B and 9C)

To a solution of 4-(3-fluorophenyl)-3-methyl-1H-pyrazole (**9A**, 1 g, 5.60 mmol) in 1,4-dioxane (20.0 mL) was added K_3PO_4 solution (4.75 g, 22.7 mmol) followed by adding ethyl oxirane-2-carboxylate (2.2

g, 22.7 mmol) and the reaction mixture was stirred at 100 °C for 16 h. After completion of starting material the reaction mixture was quenched with water and extracted with ethyl acetate. The organic layer was washed with water and brine, dried over sodium sulphate, filtered and concentrated under reduced pressure to afford the desired crude compound as two isomers. Further the two isomers were separated by flash chromatography using 30 % ethyl acetate/hexane as an eluent to afford the desired product mixture of ethyl 3-(4-(3-fluorophenyl)-3-methyl-1H-pyrazol-1-yl)-2-hydroxypropanoate (**9B**) and ethyl 3-(4-(3-fluorophenyl)-5-methyl-1H-pyrazol-1-yl)-2-hydroxypropanoate (**9C**) as off-white solids after separation by column chromatography. **9B:** ^{1}H NMR (400 MHz, DMSO-d_6): δ 7.93 (s, 1H), 7.44 - 7.36 (m, 1H), 7.28 - 7.18 (m, 2H), 7.04 - 7.00 (m, 1H), 5.82 (d, *J* = 6.0 Hz, 1H), 4.42 - 4.38 (m, 1H), 4.30 – 4.25 (dd, *J* = 4.4 Hz, 1H), 4.19 – 4.03 (m, 3H), 2.28 (s, 3H), 1.21 - 1.14 (m, 3H). MS (ESI$^+$) *m/z* 293.1 (M+H)$^+$.

9C: ^{1}H NMR (400 MHz, DMSO-d_6): δ 7.66 (s, 1H), 7.44 - 7.38 (m, 1H), 7.22 - 7.17 (m, 2H), 7.06 - 7.01 (m, 1H), 5.80 (d, *J* = 6.4 Hz, 1H), 4.44 - 4.39 (m, 1H), 4.34 – 4.22 (m, 2H), 4.12 – 4.07 (m, 2H), 2.37 (s, 3H), 1.21 - 1.14 (t, *J* = 7.2 Hz, 3H). MS (ESI$^+$) *m/z* 293.1 (M+H)$^+$.

3-(4-(3-Fluorophenyl)-3-methyl-1H-pyrazol-1-yl)-2-hydroxypropanoic acid (9D)

To a solution of ethyl 3-(4-(3-fluorophenyl)-3-methyl-1H-pyrazol-1-yl)-2-hydroxypropanoate and ethyl 3-(4-(3-fluorophenyl)-5-methyl-1H-pyrazol-1-yl)-2-hydroxypropanoate (**9B**, 0.27 g, 0.92 mmol) in methanol (10.0 ml) and water (10 mL) was added LiOH.H_2O (0.077 g, 0.8 mmol). Then the reaction mixture was stirred at room temperature for 12 h. After completion of starting material the reaction mixture was concentrated under reduced pressure to obtain the crude product. The crude product was acidified with 1N HCl solution to pH~3. The solid obtained was filtered and dried to afford a mixture of 3-(4-(3-fluorophenyl)-3-methyl-1H-pyrazol-1-yl)-2-hydroxypropanoic acid and 3-(4-(3-fluorophenyl)-5-methyl-1H-pyrazol-1-yl)-2-hydroxypropanoic acid as off-white solid (**9D,** 0.15 g, 61 %). ^{1}H NMR (400 MHz, DMSO-d_6): δ 13.60 - 12.00 (br s, 1H), 7.66 (s, 1H), 7.44 - 7.38 (m, 1H), 7.24 -7.17 (m, 2H), 7.06 - 7.02 (m, 1H), 6.00 - 5.00 (br s, 1H), 4.35 - 4.31 (m, 2H), 4.25 - 4.19 (m, 1H), 2.38 (s, 3H). MS (ESI$^+$) *m/z* 265.1 (M+H)$^+$.

3-(4-(3-Fluorophenyl)-3-methyl-1H-pyrazol-1-yl)-2-hydroxy-1-(4-(isoquinolin-5-yl)piperazin-1-yl)propan-1-one 2,2,2-trifluoroacetate (9)

To a solution 3-(4-(3-fluorophenyl)-3-methyl-1H-pyrazol-1-yl)-2-hydroxypropanoic acid and 3-(4-(3-fluorophenyl)-5-methyl-1H-pyrazol-1-yl)-2-hydroxypropanoic acid **(9D** and **9E**, 0.2 g, 0.75 mmol) in DMF (10.0 mL) were added triethylamine (0.52 mL, 3.75 mmol), 5-(piperazin-1-yl)isoquinoline hydrochloride (0.28 g, 1.13 mmol) and HBTU (0.42 g, 1.13 mmol) at 0 °C and the mixture was stirred at room temperature for 12 h. After completion of starting material the reaction mixture was quenched with ice water (10.0 mL) and extracted with DCM. The organic layer was washed with saturated $NaHCO_3$ solution, brine, dried over sodium sulphate, filtered and concentrated under reduced pressure to obtain the crude product. The crude product was purified by preparative HPLC (Column: Chemsil C18 (250mm*4.6mm*5μm), gradient of 0.1% TFA in water and acetonitril, flow rate: 1.0 mL/min) to afford 3-(4-(3-fluorophenyl)-3-methyl-1H-pyrazol-1-yl)-2-hydroxy-1-(4-(isoquinolin-5-yl)piperazin-1-yl)propan-1-one 2,2,2-trifluoroacetate as yellow solid (**9**, 0.13 g, 38 %). ^{1}H NMR (400 MHz, DMSO-d_6): δ 9.58 (s, 1H), 8.58 (d, *J* = 5.6 Hz, 1H), 8.26 (d, *J* = 5.6 Hz, 1H), 8.04 - 7.96 (m, 2H), 7.75 (t, *J* = 7.6 Hz, 1H), 7.52 (d, *J* = 7.2 Hz, 1H), 7.43 - 7.38 (m, 1H), 7.27 - 7.20 (m, 2H), 7.03 (t, *J* = 8.4 Hz, 1H), 5.5 (br s, 1H), 4.75 - 4.85 (m, 1H), 4.33 - 4.30 (m, 1H), 4.19 - 4.13 (m, 1H), 3.90 - 3.70 (m, 4H), 3.21 (s, 3H), 3.10 - 2.90 (m, 4H) MS (ESI$^+$) *m/z* 460.3 (M+H)$^+$. HPLC Purity @ 220 nm, 99.9 %.

The following compounds (**10–17**) were synthesized using the procedure described for compound **1**:

3-(4-(3,4-Difluorophenyl)-3-methyl-1H-pyrazol-1-yl)-2-hydroxy-1-(4-(isoquinolin-5-yl)piperazin-1-yl)-2-methylpropan-1-one 2,2,2-trifluoroacetate (10)

^{1}HNMR (400 MHz, DMSO-d_6): δ 9.56 (s, 1H), 8.57 (d, *J* = 6.0 Hz, 1H), 8.24 (d, *J* = 6.4 Hz, 1H), 7.97 (d, *J* = 8.0 Hz, 1H), 7.9 (s, 1H), 7.77 – 7.54 (m, 1H), 7.52 – 7.46 (m, 1H), 7.44 -7.40 (m, 2H), 7.24 – 7.22 (m, 1H), 5.94 (s, 1H), 4.35 (s, 2H), 4.16 (br s, 2H), 3.79 (br s, 2H), 3.14 (br s, 4H), 2.29 (s, 3H), 1.33 (s, 3H). MS (ESI$^+$) *m/z* 492.2 (M+H)$^+$. HPLC purity: 99.6 %.

1-(4-(6-Fluoroisoquinolin-5-yl)piperazin-1-yl)-3-(4-(3-fluorophenyl)-3-methyl-1H-pyrazol-1-yl)-2-hydroxy-2-methylpropan-1-one (11)

^{1}HNMR (400 MHz, DMSO-d_6): δ 9.28 (s, 1H), 8.53 (d, *J* = 5.6 Hz, 1H), 8.10 (d, *J* = 6.0 Hz, 1H), 8.01-7.98 (m, 1H), 7.92 (s, 1H), 7.53 (q, *J* = 9.2 Hz, 3.2 Hz, 1H), 7.40 (q, *J* = 7.6 Hz, 7.2 Hz, 1H), 7.27 – 7.20 (m, 2H), 7.03 (t, *J* = 6.8 Hz, 1H), 5.95 (s, 1H), 4.32 (s, 2H), 3.27 (br s, 4H), 3.13 (br s, 4H), 2.32 (s, 3H), 1.32 (s, 3H); MS (ESI$^+$) *m/z* 492.1 (M+H)$^+$. HPLC purity: 98.2 %.

2-Hydroxy-1-(4-(isoquinolin-5-yl)piperazin-1-yl)-3-(4-(3-methoxyphenyl) -3-methyl-1H-pyrazol-1-yl)-2-methylpropan-1-one (12)

^{1}HNMR (400 MHz, DMSO-d_6): δ 9.26 (s, 1H), 8.50 – 8.49 (m, 1 H), 7.95 – 7.92 (m, 2H), 7.79 – 7.77 (m, 1H), 7.55 (t, *J* = 7.6 Hz, 1H), 7.41 – 7.39 (m, 1H), 7.32 – 7.30 (m, 1H), 7.27 – 7.25 (m, 2H), 7.03 – 7.01 (m, 1H), 5.94 (s, 1H), 4.32 (s, 2H), 4.16 (br s, 2 H), 3.91 (s, 3H), 3.80 (br s, 2 H), 3.02 (s, 4H), 2.31 (s, 3H), 1.33 (s, 3H). MS (ESI$^+$) *m/z* 486.2 (M+H)$^+$. HPLC purity: 98.5 %.

5-(4-(3-(4-(3-Fluorophenyl)-3-methyl-1H-pyrazol-1-yl)-2-hydroxy-2-me-thylpropanoyl)piperazin-1-yl)isoquinoline 2-oxide (13)

^{1}H NMR (400 MHz, DMSO-d_6): δ 8.89 (s, 1H), 8.08 (d, *J* = 6.0 Hz, 1H), 7.99 (d, *J* = 7.6 Hz, 1H), 7.91 (s, 1H), 7.54 (d, *J* = 4.4 Hz, 2H), 7.42 - 7.37 (m, 1H); 7.26 - 7.16 (m, 3H), 7.04 -7.00 (m, 1H), 5.93 (s, 1H), 4.31 (s, 2H), 4.14 (br s, 2H), 3.77 (br s, 2H), 2.99 (s, 4H), 2.31 (s, 3H), 1.32 (s, 3H). MS (ESI$^+$) *m/z* 534.2 (M+H)$^+$. HPLC Purity @ 260 nm, 96.5 %.

3-(4-(3-Fluorophenyl)-3-methyl-1H-pyrazol-1-yl)-2-hydroxy-2-methyl-1-(4-(2-methylthieno[2,3-c]pyridin-3-yl)piperazin-1-yl)propan-1-one (14)

^{1}HNMR (400 MHz, DMSO-d_6): δ 9.30 (s, 1H), 8.48 (br s, 1H), 7.93 (s, 1H), 7.41 – 7.39 (m, 1H), 7.27 – 7.21 (m, 3H), 7.03 (br s, 1H), 5.93 (s, 1H), 4.32 (s, 2H), 4.12 (br s, 4H), 3.18 (br s, 4H), 2.64 (s, 3H), 2.32 (s, 3H), 1.33 (s, 3H). MS (ESI$^+$) *m/z* 494.2 (M+H)$^+$. HPLC purity: 98.3 %.

3-(4-(3-Fluorophenyl)-3-methyl-1H-pyrazol-1-yl)-2-hydroxy-2-methyl-1-(4-(phthalazin-5-yl)piperazin-1-yl)propan-1-one (15)

Yield 19 %. ^{1}HNMR (400 MHz, DMSO-d_6): δ 9.74 (s, 1H), 9.61 (s, 1H), 7.90 (d, *J* = 8.0 Hz, 2H), 7.76 (d, *J* = 8.0 Hz, 1H), 7.51 (d, *J* = 8.0 Hz, 1H), 7.43 – 7.37 (m, 1H), 7.27 – 7.20 (m, 2H), 7.05 – 7.01 (m, 1H), 4.32 (s, 2H), 4.22 (br s, 2H), 3.80 (br s, 2H), 3.11 (s, 4H), 2.31 (s, 3H), 1.31 (s, 3H), 1.22 (s, 1H). MS (ESI$^+$) *m/z* 475.2 (M+H)$^+$. HPLC purity: 93.1 %.

3-(4-(3-Fluorophenyl)-3-methyl-1H-pyrazol-1-yl)-2-hydroxy-2-methyl-1-(4-(quinazolin-8-yl)piperazin-1-yl)propan-1-one (16)

Yield 33 %. ^{1}HNMR (400 MHz, DMSO-d_6): δ 9.51 (s, 1H), 9.27 (s, 1H), 7.91 (s, 1H), 7.67 – 7.65 (m, 1H), 7.63 – 7.61 (m, 1H), 7.59 – 7.42 (m, 1H), 7.40 – 7.38 (m, 1H), 7.36 – 7.35 (m, 1H), 7.26 – 7.22 (m, 2H),

7.04 – 7.06 (m, 1H), 5.92 (s, 1H), 4.35 (s, 1H), 4.15 (br s, 2H), 3.75 (br s, 2H), 3.36 (s, 4H), 2.30 (s, 3H), 1.32 (s, 3H). MS (ESI$^+$) *m/z* 475.2 (M+H)$^+$. HPLC purity: 99.7 %.

3-(4-(3-Fluorophenyl)-3-methyl-1H-pyrazol-1-yl)-2-hydroxy-1-(4-(imidazo[1,2-a]pyrazin-3-yl)piperazin-1-yl)-2-methylpropan-1-one (17)

^{1}HNMR (400 MHz, DMSO-d_6): δ 8.93 (s, 1H), 8.28 (d, *J* = 3.2 Hz, 1H), 7.91 (s, 1H), 7.84 (d, *J* = 4.4 Hz, 1H), 7.50 (s, 1H), 7.42 – 7.37 (m, 1H), 7.26 – 7.2 (m, 2H), 7.02 (t, *J* = 7.6 Hz, 1H), 5.90 (s, 1H), 4.30 (s, 2H), 4.12 (br s, 2H), 3.72 (br s, 2H), 3.02 (br s, 4H), 2.30 (s, 3H), 1.31 (s, 3H). MS (ESI$^+$) *m/z* 464.2 (M+H)$^+$. HPLC purity: 93.1 %.

Synthesis of 1-(4-(1-benzyl-1H-indazol-5-yl)piperazin-1-yl)-3-(4-(3-fluorophenyl)-3-methyl-1H-pyrazol-1-yl)-2-hydroxy-2-methylpropan-1-one (18)

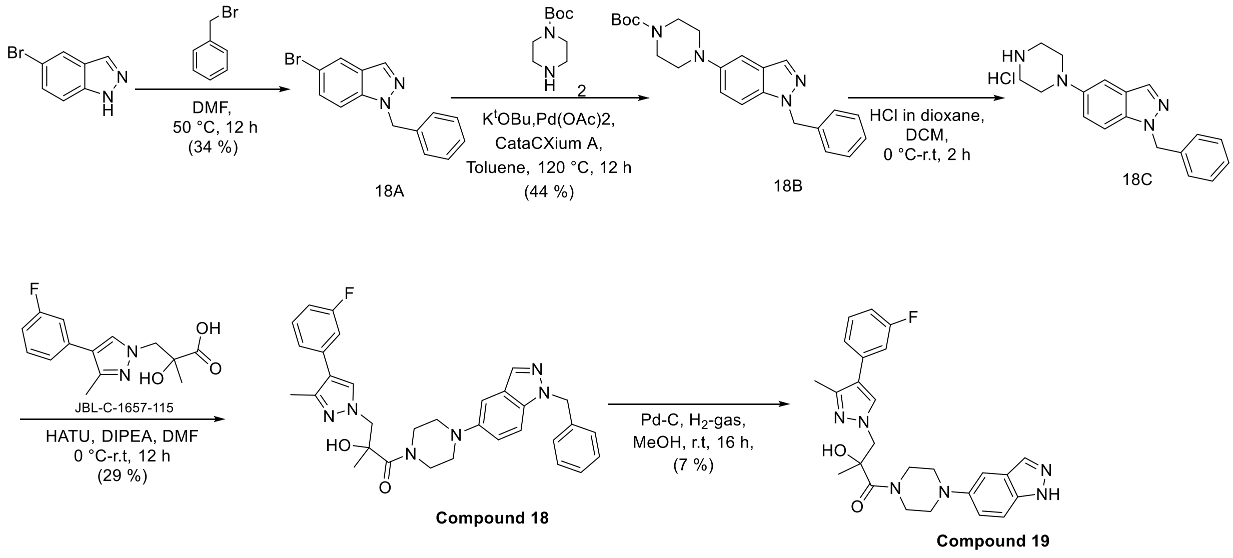

1-Benzyl-5-bromo-1H-indazole (18A)

To a solution of 5-bromo-1H-indazole (2 g, 10.1 mmol) in DMF (15.0 mL) was added benzyl bromide (2.39 mL, 20.2 mmol) and the reaction mixture was stirred at 50 °C for 12 h. After completion of starting material the reaction mixture was quenched with water (60 mL) and the product was extracted with ethyl acetate. The organic layer was washed with ice-water and brine, dried over sodium sulphate, filtered and concentrated under reduced pressure affording the desired crude compound. The obtained crude was purified by flash chromatography using 20% ethyl acetate in hexane as eluent to give 1-benzyl-5-bromo-1H-indazole as off-white solid (**18A**, 1.0 g, 34 %). ^{1}H NMR (400 MHz, DMSO-d_6): δ 8.45 (s, 1H), 7.98 - 7.94 (m, 1H), 7.56 (d, *J* = 9.2 Hz, 1H), 7.27 - 7.25 (m, 6H), 5.62 (s, 2H). MS (ESI$^+$) *m/z* 288.2 (M+H)$^+$.

Tert-butyl 4-(1-benzyl-1H-indazol-5-yl)piperazine-1-carboxylate (18B)

To a solution of 1-benzyl-5-bromo-1H-indazole (**18A**, 0.3 g, 1 mmol) in toluene (10.0 mL) were added N-Boc piperazine (0.29 g, 1.5 mmol), potassium tertiary butoxide (0.168 g, 1.5 mmol), CatacXium A (0.033 g, 0.05 mmol) and palladium acetate (0.053 g, 0.15 mmol) under argon atmosphere. The reaction mixture was heated at 120 °C for 12 h. After completion of starting material the reaction mixture was filtered through a celite pad and washed with ethyl acetate. The filtrate layer was washed with water and brine, dried over sodium sulphate, filtered and concentrated under reduced pressure to get the desired crude compound. The obtained crude product was purified by flash chromatography using 20 % ethyl acetate in hexane as eluent to afford tert-butyl 4-(1-benzyl-1H-indazol-5-yl)piperazine-1-carboxylate as yellow sticky solid (**18B**, 0.18 g, 44 %). ^{1}H NMR (400 MHz, DMSO-d_6): δ 8.22 (s, 1H); 7.46

(d, J = 9.2 Hz, 1H), 7.33 - 7.24 (m, 5H), 7.10 (d, J = 9.2 Hz, 1H), 6.92 (br s, 1H), 5.54 (s, 2H), 3.50 - 3.45 (m, 4H), 3.05 - 2.95 (m, 4H), 1.40 (s, 9H). MS (ESI^+) *m/z* 393.2 $(M+H)^+$.

1-Benzyl-5-(piperazin-1-yl)-1H-indazole hydrochloride (18C)

To a solution of tert-butyl 4-(1-benzyl-1H-indazol-5-yl)piperazine-1-carboxylate (**18B**, 0.13 g, 0.33 mmol) in DCM (8.0 ml) was added 4N HCl in dioxane (2.0 mL) at 0 °C and the reaction mixture was stirred for 2 h at room temperature. After completion of starting material the reaction mixture was concentrated under reduced pressure giving the desired crude compound as HCl salt. The obtained solid was triturated with a mixture of pentane and diethyl ether (1:1 ratio) and dried to afford 1-benzyl-5-(piperazin-1-yl)-1H-indazole hydrochloride as brown thick solid (**18C**, 0.35 g). MS (ESI^+) *m/z* 293.2 $(M+H)^+$.

1-(4-(1-Benzyl-1H-indazol-5-yl)piperazin-1-yl)-3-(4-(3-fluorophenyl)-3-methyl-1H-pyrazol-1-yl)-2-hydroxy-2-methylpropan-1-one (18)

To a solution 1-benzyl-5-(piperazin-1-yl)-1H-indazole hydrochloride (**18C**, 0.115 g, 0.35 mmol) in DMF (10.0 mL) were added N,N-diisopropylethylamine (0.3 mL, 0.52 mmol), 3-(4-(3-fluorophenyl)-3-methyl-1H-pyrazol-1-yl)-2-hydroxy-2-methylpropanoic acid (0.146 g, 0.52 mmol) and HATU (0.197 g, 1.75 mmol) at 0 °C and the reaction mixture was stirred at room temperature for 12 h. After completion of starting material the reaction mixture was quenched with ice water (10.0 mL) and extracted with DCM. The organic layer was washed with saturated $NaHCO_3$ solution, brine, dried over sodium sulphate, filtered and concentrated under reduced pressure to obtain the crude product which was purified by flash chromatography using 5% methanol in DCM as eluent to afford the desired product as off-white solid (**18**, 0.056 g, 29 %). ^1H NMR (400 MHz, DMSO-d_6): δ 8.20 (s, 1H), 7.89 (s, 1H), 7.46 - 7.35 (m, 2H), 7.31 - 7.18 (m, 7H), 7.12 (d, J = 9.2 Hz, 1H), 7.01 (t, J = 7.6 Hz, 1H), 6.89 (br s, 1H), 5.91 (s, 1H), 5.54 (s, 2H), 4.35 - 4.29 (m, 2H), 4.25 - 3.50 (m, 4H), 3.05 - 2.95 (m, 4H), 2.28 (s, 3H), 1.30 (s, 3H). MS (ESI^+) *m/z* 553.0 $(M+H)^+$. HPLC Purity @ 270 nm, 99.3 %.

1-(4-(1H-Indazol-5-yl)piperazin-1-yl)-3-(4-(3-fluorophenyl)-3-methyl-1H-pyrazol-1-yl)-2-hydroxy-2-methylpropan-1-one (19)

To a solution of 1-(4-(1-benzyl-1H-indazol-5-yl)piperazin-1-yl)-3-(4-(3-fluorophenyl)-3-methyl-1H-pyrazol-1-yl)-2-hydroxy-2-methylpropan-1-one (**18**, 0.068 g, 0.12 mmol) methanol (10.0 mL) was added 10% Pd/C (0.02 g) and the suspension was stirred for 12 h under H_2 atmosphere. After completion of the starting material the reaction mixture was filtered through celite pad and the filtrate was concentrated yielding the crude product. The obtained crude was purified by flash chromatography using 5% MeOH in DCM as eluent to afford the desired product as off-white solid (**19**, 0.004 g, 7 %). ^1H NMR (400 MHz, DMSO-d_6): δ 12.77 (s, 1H), 7.90 (s, 1H), 7.86 (s, 1H), 7.40 - 7.36 (m, 2H), 7.25 - 7.16 (m, 3H), 7.08 (s, 1H), 7.02 (t, J = 9.2 Hz, 1H), 5.91 (s, 1H), 4.29 (s, 2H), 4.15 - 3.55 (m, 4H), 3.10 - 2.95 (m, 4H), 2.28 (s, 3H), 1.30 (s, 3H). MS (ESI^+) *m/z* 463.0 $(M+H)^+$. HPLC Purity @ 258 nm, 98.7 %.

Synthesis of 1-(4-(1H-indazol-4-yl)piperazin-1-yl)-3-(4-(3-fluorophenyl)-3-methyl-1H-pyrazol-1-yl)-2-hydroxy-2-methylpropan-1-one (20)

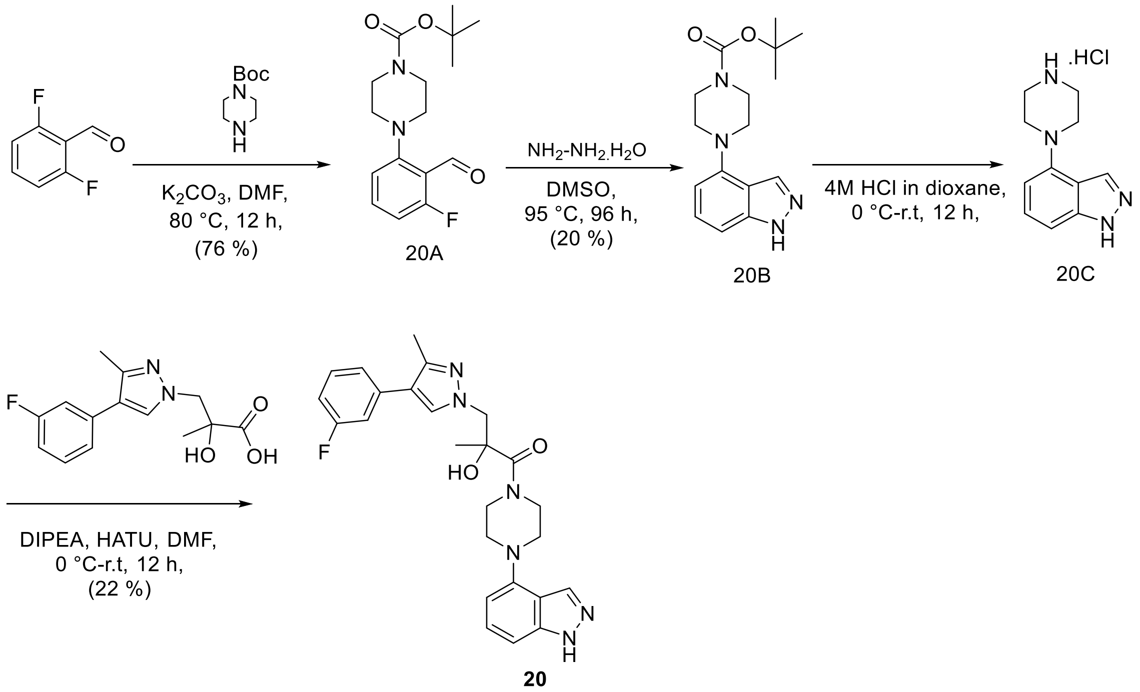

Tert-butyl 4-(3-fluoro-2-formylphenyl)piperazine-1-carboxylate (20A)

To a solution of 2,6-difluorobenzaldehyde (1 g, 7 mmol) in DMF (15.0 mL) were added N-Boc piperazine (1.44 g, 7.7 mmol) and potassium carbonate (1.1 g, 8.4 mmol) and the reaction mixture was stirred at 80 °C for 12 h. After completion of the starting material the reaction mixture was diluted with ice water and extracted with ethyl acetate. The organic layer was washed with water and brine, dried over sodium sulphate, filtered and concentrated under reduced pressure yielding the desired crude compound. The obtained crude product was purified by flash chromatography using 30 % ethyl acetate in hexane as eluent to afford tert-butyl 4-(3-fluoro-2-formylphenyl)piperazine-1-carboxylate as yellow solid (**20A**, 1.6 g, 76 %). ^{1}H NMR (400 MHz, $CDCl_3$): δ 10.31 (s, 1H), 7.48 - 7.43 (m, 1H), 6.83 - 6.75 (m, 2H), 3.65 - 3.62 (m, 4H), 3.10-3.00 (m, 4H), 1.48 (s, 9H). MS (ESI$^+$) *m/z* 309.2 (M+H)$^+$.

Tert-butyl 4-(1H-indazol-4-yl)piperazine-1-carboxylate (20B)

To a solution of tert-butyl 4-(3-fluoro-2-formylphenyl) piperazine-1-carboxylate (**20A**, 0.5 g, 1.6 mmol) in DMSO (5.0 mL) was added 80% hydrazine hydrate solution (5.0 mL) and the reaction mixture was stirred at 95 °C for 96 h. After completion of the starting material the reaction mixture was diluted with ice water and extracted with ethyl acetate. The organic layer was washed with water and brine, dried over sodium sulphate, filtered and concentrated under reduced pressure giving the desired crude compound. The obtained crude product was purified by flash chromatography using 20 % ethyl acetate in hexane as eluent to afford tert-butyl 4-(1H-indazol-4-yl)piperazine-1-carboxylate as a yellow liquid (**20B**, 0.1 g, 20 %). MS (ESI$^+$) *m/z* 303.2 (M+H)$^+$.

4-(Piperazin-1-yl)-1H-indazole hydrochloride (20C)

To a solution of tert-butyl 4-(1H-indazol-4-yl)piperazine-1-carboxylate (**20B**, 0.1 g, 0.33 mmol) in DCM (5 ml) was added 4M HCl in dioxane (0.5 mL) at 0 °C and the reaction mixture was stirred for 12 h at room temperature. After completion of starting material the reaction mixture was concentrated under reduced pressure giving the desired crude compound as HCl salt. The obtained solid was

triturated with a mixture of pentane and diethyl ether (1:1 ratio) and dried to afford 4-(piperazin-1-yl)-1H-indazole hydrochloride as an off white solid (**20C**, 0.1 g). MS (ESI$^+$) *m/z* 203.1 (M+H)$^+$.

1-(4-(1H-Indazol-4-yl)piperazin-1-yl)-3-(4-(3-fluorophenyl)-3-methyl-1H-pyrazol-1-yl)-2-hydroxy-2-methylpropan-1-one (20)

To a solution 4-(piperazin-1-yl)-1H-indazole hydrochloride (**20C**, 0.07 g, 0.29 mmol) in DMF (10.0 mL) were added DIPEA (0.25 mL, 1.45 mmol), 3-(4-(3-fluorophenyl)-3-methyl-1H-pyrazol-1-yl)-2-hydroxy-2-methylpropanoic acid (0.12 g, 0.44 mmol) and HATU (0.16 g, 0.44 mmol) at 0 °C and the reaction mixture was stirred at room temperature for 12 h. After completion of starting material the reaction mixture was quenched with ice water (10.0 mL) and extracted with DCM. The organic layer was washed with saturated NaHCO3 solution, brine, dried over sodium sulphate, filtered and concentrated under reduced pressure to give the crude product. The crude product was purified by flash chromatography using 5 % MeOH in DCM as an eluent to afford 1-(4-(1H-indazol-4-yl)piperazin-1-yl)-3-(4-(3-fluorophenyl)-3-methyl-1H-pyrazol-1-yl)-2-hydroxy-2-methylpropan-1-one as an off-white solid (**20**, 0.03 g, 22 %). ^{1}H NMR (400 MHz, DMSO-d_6): δ 12.93 (s, 1H), 8.10 (s, 1H), 7.90 (s, 1H), 7.42 - 7.36 (m, 1H), 7.36 - 7.25 (m, 3H), 6.95 - 7.08 (m, 2H), 6.40 (d, *J* = 7.2 Hz, 1H), 5.92 (s, 1H), 4.26 (s, 2H), 4.20 - 3.60 (m, 4H), 3.25 - 3.15 (m, 4H), 2.29 (s, 3H), 1.31 (s, 3H). MS (ESI$^+$) *m/z* 461.3 (M-H)$^+$. HPLC Purity @ 290 nm, 99.2 %.

The following compounds **(21–26**) were synthesized using the procedure described for compound **1**:

5-(4-(3-(4-(3-Fluorophenyl)-3-methyl-1H-pyrazol-1-yl)-2-hydroxy-2-methylpropanoyl)piperazin-1-yl)quinolin-2(1H)-one (21)

^{1}H NMR (400 MHz, DMSO-d_6): δ 11.66 (s, 1H), 8.04 (d, *J* = 10.0 Hz, 1H), 7.90 (s, 1H), 7.41 - 7.35 (m, 2H), 7.26 - 7.19 (m, 2H), 7.05 - 6.99 (m, 2H), 6.78 (d, *J* = 7.6 Hz, 1H), 6.44 (d, *J* = 9.6 Hz, 1H), 5.92 (s, 1H), 4.30 (s, 2H), 4.07 (br s, 2H), 3.71 (br s, 2H), 2.91 (s, 4H), 2.30 (s, 3H), 1.31 (s, 3H). MS (ESI$^+$) *m/z* 490.2 (M+H)$^+$. HPLC Purity @ 260 nm, 99.1 %.

5-(4-(3-(4-(3-Fluorophenyl)-3-methyl-1H-pyrazol-1-yl)-2-hydroxy-2-methyl-propanoyl)piperazin-1-yl)isoquinolin-1(2H)-one (22)

Yield 19 %. ^{1}HNMR (400 MHz, DMSO-d_6): δ 11.20 (s, 1H), 7.91 (s, 1H), 7.89 – 7.87 (d, *J* = 8.0 Hz, 1H), 7.42 – 7.39 (m, 2H), 7.39 – 7.37 (m, 1H), 7.35 – 7.30 (m, 1H), 7.28 – 7.26 (m, 1H), 7.24 – 7.22 (m, 1H), 7.02 (t, *J* = 4.8 Hz, 1H), 5.92 (s, 1H), 4.30 (br s, 2H), 3.70 (br s, 4H), 3.11 (s, 1 H), 2.92 (s, 4H), 2.30 (s, 3H), 1.31 (s, 3H). MS (ESI$^+$) *m/z* 490.2 (M+H)$^+$. HPLC purity: 98.9 %.

5-(4-(3-(4-(3-Fluorophenyl)-3-methyl-1H-pyrazol-1-yl)-2-hydroxy-2-methylpropanoyl)piperazin-1-yl)-1-methylquinolin-2(1H)-one (23)

^{1}HNMR (400 MHz, DMSO-d_6): δ 8.11 (d, *J* = 10.4 Hz, 1H), 7.91 (s, 1H), 7.54 – 7.50 (m, 1H), 7.42 – 7.37 (m, 1H), 7.30 -7.19 (m, 3H), 7.04 – 7.00 (m, 1H), 6.92 (d, *J* = 7.6 Hz, 1H), 6.57 (d, *J* = 9.6 Hz, 1H), 5.92 (s, 1H), 4.34 (s, 2H), 4.27 (br s, 2H), 3.75 (br s, 2H), 3.58 (s, 1H), 2.92 (br s, 4H), 2.30 (s, 3H), 1.31 (s, 3H). MS (ESI$^+$) *m/z* 504.2 (M+H)$^+$. HPLC purity: 99.6 %.

8-(4-(3-(4-(3-Fluorophenyl)-3-methyl-1H-pyrazol-1-yl)-2-hydroxy-2-methylpropanoyl)piperazin-1-yl)-2H-benzo[b][1,4]oxazin-3(4H)-one (24)

^{1}HNMR (400 MHz, DMSO-d_6): δ 10.55 (s, 1H), 7.89 (s, 1H), 7.42 – 7.37 (m, 1H), 7.25 – 7.19 (m, 2H), 7.02 (t, *J* = 8.4 Hz, 1H), 6.83 (t, *J* = 7.6 Hz, 1H), 6.54 (t, *J* = 6.8 Hz, 2H), 5.88 (s, 1H), 4.52 (s, 2H), 4.24 (s, 2H), 4.00 (br s, 2H), 3.6 (br s, 2H), 2.94(br s, 4H), 2.29 (s, 3H), 1.29 (s, 3H). MS (ESI$^+$) *m/z* 494.2 (M+H)$^+$. HPLC purity: 98.6 %.

5-(1-(3-(4-(3-Fluorophenyl)-3-methyl-1H-pyrazol-1-yl)-2-hydroxy-2-methylpropanoyl)piperidin-4-yl)quinolin-2(1H)-one (25)

^{1}HNMR (400 MHz, DMSO-d_6): δ 11.69 (s, 1H), 8.23 (d, *J* = 10 Hz, 1H), 7.91 (s, 1H), 7.42 - 7.37 (m, 2H), 7.26 - 7.24 (m, 1H), 7.22 - 7.02 (m, 2H), 7.00 (br s, 2H), 6.50 (d, *J* = 10.0 Hz, 1H), 5.85 (br s, 1H), 4.90 (br s, 1H), 4.65 (br s, 1H), 4.30 (br s, 2H), 3.45 (br s, 1H), 3.14 (br s, 1H), 2.79 (br s, 1H), 2.31 (s, 3H), 1.85 - 1.74 (m, 3H), 1.62 - 1.45 (m, 2H), 1.30 (s, 3H). MS (ESI$^+$) *m/z* 489.4 $(M+H)^+$. HPLC purity: 100 %.

5-(1-(3-(4-(3-Fluorophenyl)-3-methyl-1H-pyrazol-1-yl)-2-hydroxy-2-methylpropanoyl)pyrrolidin-3-yl)-4a,8a-dihydroquinolin-2(1H)-one (26)

^{1}HNMR (400 MHz, DMSO-d_6): δ 11.43 (s, 1H), 8.07 (br s, 1H), 7.84 (s, 1H), 7.41 - 7.37 (m, 2H), 7.28 - 7.15 (m, 3H), 7.02 - 6.98 (m, 2H), 6.42 (d, *J* = 8.4 Hz, 1H), 5.40 (s, 1H), 4.36 - 4.33 (m, 1H), 4.23 - 4.20 (m, 1H), 3.99 (br s, 1H), 3.81 (br s, 1H), 3.60 (br s, 2H), 2.31 (s, 3H), 2.21 (br s, 1H), 1.92 -1.85 (m, 1H), 1.34 (s, 3H). MS (ESI$^+$) *m/z* 475.2 $(M+H)^+$. HPLC purity: 99.8 %.

Synthesis of 5-(1-(3-(4-(3-fluorophenyl)-3-methyl-1H-pyrazol-1-yl)-2-hydroxy-2-methylpropanoyl)azetidin-3-yl)quinolin-2(1H)-one (27)

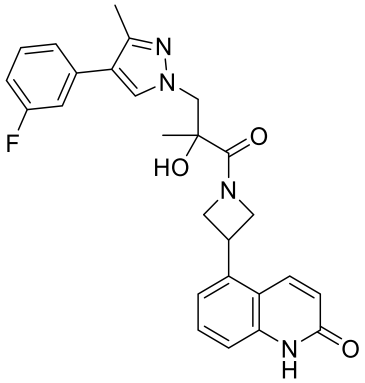

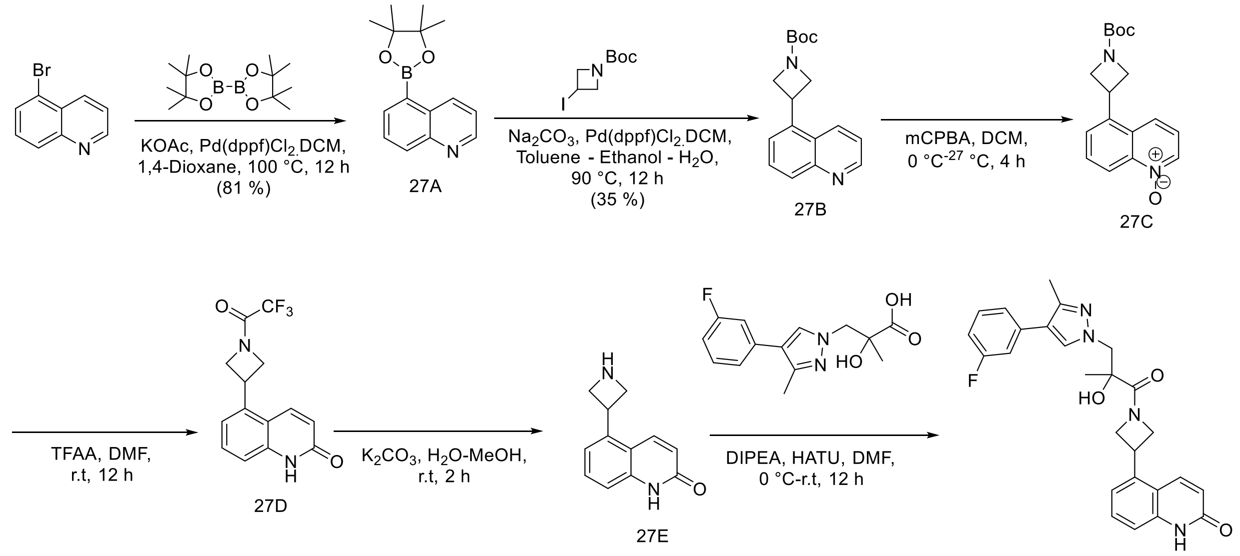

5-(4,4,5,5-Tetramethyl-1,3,2-dioxaborolan-2-yl)quinolone (27A)

To a solution of 5-bromoquinoline (2 g, 9.6 mmol) in 1,4-dioxane (15.0 mL) were added bis(pinacolato)diboron (3.6 g, 14.4 mmol), potassium acetate (2.8 g, 28.8 mmol) and Pd(dppf)Cl_2.DCM (0.78 g, 0.96 mmol) under argon atmosphere and the reaction mixture was stirred at 100 °C for 12 h. After completion of starting material the reaction mixture was diluted with ethyl acetate and filtered

through a celite pad and washed with ethyl acetate. The ethyl acetate layer was washed with water and brine, dried over sodium sulphate, filtered and concentrated under reduced pressure giving the desired crude compound. The obtained crude product was purified by flash chromatography using 20 % ethyl acetate in hexane as eluent to afford 5-(4,4,5,5-tetramethyl-1,3,2-dioxaborolan-2-yl)quinoline as off-white solid (**27A**, 2 g, 81 %). ^{1}H NMR (400 MHz, DMSO-d_6): δ 8.98 (d, *J* = 8.4 Hz, 1H), 8.90 - 8.89 (m, 1H), 8.13 (d, *J* = 8.4 Hz, 1H), 8.01 (d, *J* = 10 Hz, 1H), 7.75 (t, *J* = 7.2 Hz, 1H), 7.59 - 7.56 (m, 1H), 1.37 (s, 12H). MS (ESI$^+$) *m/z* 256.2 (M+H)$^+$.

Tert-butyl 3-(quinolin-5-yl)azetidine-1-carboxylate (27B)

To a solution of 5-(4,4,5,5-tetramethyl-1,3,2-dioxaborolan-2-yl)quinoline (**27A**, 0.5 g, 1.9 mmol) in toluene (3.0 mL)/ethanol (3.0 mL)/H_2O (3.0 mL) were added tert-butyl 3-iodoazetidine-1-carboxylate (1.1 g, 3.9 mmol), sodium carbonate (0.6 g, 5.7 mmol) and Pd(dppf)Cl_2.DCM (0.78 g, 0.19 mmol) under argon atmosphere. Then the reaction mixture was stirred at 90 °C for 12 h. It was diluted with ethyl acetate and filtered through a celite pad. The ethyl acetate layer was washed with water and brine, dried over sodium sulphate, filtered and concentrated under reduced pressure giving the desired crude compound. The obtained crude product was purified by flash chromatography using 40 % ethyl acetate in hexane as eluent to afford tert-butyl 3-(quinolin-5-yl)azetidine-1-carboxylate as yellow sticky solid (**27B**, 0.2 g, 35 %). ^{1}H NMR (400 MHz, DMSO-d_6): δ 8.94 - 8.88 (m, 1H), 8.27 (d, *J* = 8.4 Hz, 1H), 7.92 (d, *J* = 8.4 Hz, 1H), 7.74 (d, *J* = 7.2 Hz, 1H), 7.63 (d, *J* = 7.2 Hz, 1H), 7.55 - 7.52 (m, 1H), 4.54 - 4.42 (m, 2H), 4.10 - 4.00 (m, 2H), 3.90 - 3.88 (m, 1H), 1.34 (s, 9H). MS (ESI$^+$) *m/z* 285.1 (M+H)$^+$.

Tert-butyl 3-(1-(λ^1-oxidanyl)-1 λ^4-quinolin-5-yl)azetidine-1-carboxylate (27C)

To a solution of tert-butyl 3-(quinolin-5-yl)azetidine-1-carboxylate (**27B**, 0.2 g, 0.7 mmol) in DCM (10.0 mL) was added meta-chloroperbenzoic acid (0.13 g, 0.77 mmol) at 0 °C and it was stirred at room temperature for 12 h. After completion of starting material the reaction mixture was quenched with water and the organic layer was separated. The organic layer was washed with water and brine, dried over sodium sulphate, filtered and concentrated under reduced pressure yielding the desired crude tert-butyl 3-(1-(λ1-oxidanyl)-1 λ4-quinolin-5-yl)azetidine-1-carboxylate as a brown sticky solid (**27C**, 0.18 g). ^{1}H NMR (400 MHz, DMSO-d_6): δ 8.58 (d, *J* = 5.6 Hz, 1H), 8.47 (d, *J* = 8 Hz, 1H), 7.86 - 7.74 (m, 3H), 7.46 (t, *J* = 6 Hz, 1H), 4.49 - 4.39 (m, 2H), 4.05 - 3.85 (m, 3H), 1.38 (s, 9H). MS (ESI$^+$) *m/z* 301.1 (M+H)$^+$.

5-(1-(2,2,2-Trifluoroacetyl)azetidin-3-yl)quinolin-2(1H)-one (27D)

To a solution tert-butyl 3-(1-(λ^1-oxidanyl)-1λ^4-quinolin-5-yl)azetidine-1-carboxylate (**27C**, 0.18 g, 0.59 mmol) in DMF (5.0 mL) was added trifluoroacetic anhydride (0.42 mL, 2.9 mmol) at 0 °C and the reaction mixture was stirred for 12 h at room temperature. After completion of starting material the reaction mixture was quenched with water and the organic layer was separated. The organic layer was washed with water and brine, dried over sodium sulphate, filtered and concentrated under reduced pressure to yield the desired crude product as brown solid (**27D**, 0.1 g). ^{1}H NMR (400 MHz, DMSO-d_6): δ 11.78 (s, 1H), 7.85 (d, *J* = 10.4 Hz, 1H), 7.50 (t, *J* = 8.0 Hz, 1H), 7.30 - 7.23 (m, 2H), 6.50 (d, *J* = 10 Hz, 1H), 4.45 - 4.15 (m, 4H), 4.25 - 4.15 (m, 1H). MS (ESI$^+$) *m/z* 297.1 (M+H)$^+$.

5-(Azetidin-3-yl)quinolin-2(1H)-one (27E)

To a solution of 5-(1-(2,2,2-trifluoroacetyl)azetidin-3-yl)quinolin-2(1H)-one (**27D**, 0.1 g, 0.33 mmol) in MeOH (5.0 mL) and H_2O (0.5 mL) was added potassium carbonate (0.093 g, 0.67 mmol) and the reaction mixture was stirred for 4 h at room temperature. After completion of starting material the reaction mixture was quenched with water and the organic layer was separated. The organic layer was washed with water and brine, dried over sodium sulphate, filtered and concentrated under reduced pressure to yield the desired crude product. The obtained crude product was purified by flash

chromatography using 10 % methanol in DCM and 0.01 mL TEA as eluent to afford 5-(azetidin-3-yl)quinolin-2(1H)-one as a pale yellow solid **(27E**, 0.07 g). ^{1}H NMR (400 MHz, DMSO-d_6): δ 11.78 (s, 1H), 7.85 (d, *J* = 10.4 Hz, 1H), 7.50 (t, *J* = 8.0 Hz, 1H), 7.30 - 7.23 (m, 2H), 6.50 (d, *J* = 10.0 Hz, 1H), 4.65 - 4.45 (m, 4H), 4.25 - 4.15 (m, 1H). MS (ESI$^+$) *m/z* 201.1 (M+H)$^+$.

5-(1-(3-(4-(3-Fluorophenyl)-3-methyl-1H-pyrazol-1-yl)-2-hydroxy-2-methyl-propanoyl)azetidin-3-yl)quinolin-2(1H)-one (27)

To a solution 3-(4-(3-fluorophenyl)-3-methyl-1H-pyrazol-1-yl)-2-hydroxy-2-methylpropanoic acid (0.065 g, 0.23 mmol) in DMF (8.0 mL) were added DIPEA (0.12 mL, 0.69 mmol), 5-(azetidin-3-yl)quinolin-2(1H)-one (**27D**, 0.07 g, 0.35 mmol) and HATU (0.13 g, 0.35 mmol) at 0 °C and the reaction mixture was stirred at room temperature for 12 h. After completion of starting material the reaction mixture was quenched with water (10.0 mL) and extracted with ethyl acetate. The organic layer was washed with saturated $NaHCO_3$ solution, brine, dried over sodium sulphate, filtered and concentrated under reduced pressure. The obtained crude product was purified by flash chromatography to afford 5-(1-(3-(4-(3-fluorophenyl)-3-methyl-1H-pyrazol-1-yl)-2-hydroxy-2-methylpropanoyl)azetidin-3-yl)quinolin-2(1H)-one as an off-white solid (**27**, 0.007 g, 6.5 %). ^{VT}NMR (400 MHz, DMSO-d_6): δ 11.45 (s, 1H), 7.80 (br s, 1H), 7.70 (d, *J* = 9.6 Hz, 1H), 7.36 (d, *J* = 6.4 Hz, 2H), 7.20 (d, *J* = 8.0 Hz, 1H), 7.10 - 7.08 (br s, 2H), 6.99 (t, *J* = 8.4 Hz, 2H), 6.41 (d, *J* = 10.4 Hz, 1H), 5.28 (s, 1H), 4.45 - 4.30 (br s, 2H), 4.30 - 4.09 (m, 4H), 3.95 - 3.85 (m, 1H), 2.30 - 2.02 (s, 3H), 1.32 (s, 3H), MS (ESI$^+$) *m/z* 461.1 (M+H)$^+$. HPLC Purity @ 274 nm, 99.3 %.

The following compounds **(28**,**29**) were synthesized using the procedure described for compound **1**:

5-(4-(3-(4-(3-Fluorophenyl)-3-methyl-1H-pyrazol-1-yl)-2-hydroxy-2-methylpropanoyl)-1,4-diazepan-1-yl)quinolin-2(1H)-one (28)

^{1}HNMR (400 MHz, DMSO-d_6): δ 11.61 (s, 1H), 8.10 (br s, 1H), 7.91 (s, 1H), 7.39 - 7.32 (m, 2H), 7.24 - 7.18 (m, 2H), 7.02 - 7.01 (m, 1H), 6.94 (d, *J* = 8.4 Hz, 1H), 6.81(br s, 1H), 6.42 (d, *J* = 9.2 Hz, 1H), 5.82 (s, 1H), 4.35 (br s, 1H), 4.28 (br s, 1H), 4.10 (br s, 1H), 3.90 (brs, 1H), 3.70 (br s, 1H), 3.61 (br s, 1H), 3.07 (br s, 4H), 2.29 (s, 3H), 1.98 (br s, 2H), 1.33 (s, 3H). MS (ESI$^+$) *m/z* 504.2 (M+H)$^+$. HPLC purity: 98.4%.

3-(4-(3-Fluorophenyl)-3-methyl-1H-pyrazol-1-yl)-2-hydroxy-1-(3-(isoquinolin-5-yl)azetidin-1-yl)-2-methylpropan-1-one (29)

^{1}HNMR (400 MHz, DMSO-d_6 VT at 90 °C): δ 9.33 (s, 1H), 9.27 (s, 1H), 8.45 (d, *J* = 6.4 Hz, 1H), 8.01 (d, *J* = 8.0 Hz,1H), 7.80 (br s, 1H), 7.71 – 7.63 (m, 3H), 7.34 (br s, 1H), 7.00 - 6.96 (m, 3H), 4.75 (br s, 1H), 4.62 (br s, 1H), 4.42 - 4.28 (m, 4H), 4.13 - 4.10 (m, 2H), 3.99 (br s, 1H), 2.02 (br s, 3H), 1.33 (s, 3H). MS (ESI$^+$) *m/z* 445.2 (M+H)$^+$. HPLC purity: 99.9 %.

5-(1-(3-(4-(3-Fluorophenyl)-3-methyl-1H-pyrazol-1-yl)-2-hydroxy-2-methylpropanoyl)-4-hydroxypiperidin-4-yl)quinolin-2(1H)-one (30)

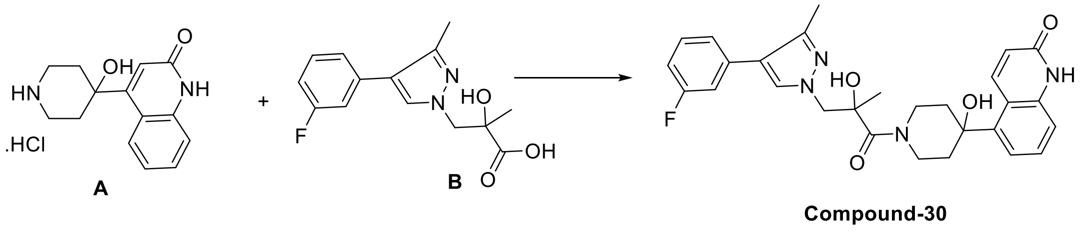

Compound **A** was prepared as described [Bentley, J. et al. Fused tricyclic derivatives for the treatment of psychotic disorders. WO2006024517 (A1)]. Compounds **B** and **30** were prepared as described for compound **27**. ^{1}HNMR (400 MHz, DMSO-d_6): δ 11.66 (s, 1H), 8.90 (d, *J* = 10.0 Hz, 1H), 7.89 (s, 1H), 7.39 - 7.38 (m, 2H), 7.25 - 7.17 (m, 4H), 7.03 - 6.99 (m, 1H), 6.44 (d, *J* = 10.0 Hz, 1H), 5.82 (s, 1H),

5.51 (s, 1H), 4.70 - 4.61 (m, 1H), 4.28 (s, 3H), 3.48 (br s, 1H), 3.11 (br s, 1H), 2.25 (s, 3H), 1.98 - 1.96 (m, 4H), 1.29 (s, 3H). MS (ESI$^+$) *m/z* 505.2 (M+H)$^+$. HPLC purity: 99.3 %.

3-(4-(3-Fluorophenyl)-3-methyl-1H-pyrazol-1-yl)-2-hydroxy-1-(4-hydroxy-4-(isoquinolin-5-yl)piperidin-1-yl)-2-methylpropan-1-one (31)

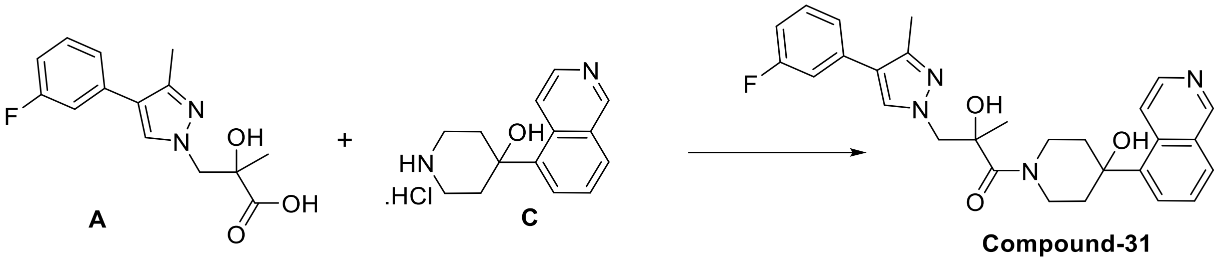

Compound **C** was prepared as described in WO2006/24517, 2006, A1 page;98, Compounds **A** and **31** were prepared as described for compound **27**. ^{1}HNMR (400 MHz, DMSO-d_6): δ 9.22 (s, 1H), 8.66 (d, *J* = 6.5 Hz, 1H), 8.66 (d, *J* = 6.0 Hz, 1H), 8.01 (d, *J* = 8.0 Hz, 1H), 7.90 (s, 1H), 7.74 (s, 1H), 7.60 - 7.58 (m, 1H), 7.39 - 7.37 (m, 1H), 7.20 (s, 2H), 7.03 - 6.99 (m, 1H), 5.83 (s, 1H), 5.51 (s, 1H), 4.62 (br s, 1H), 4.30 (s, 3H), 3.55 (br s, 1H), 3.20 (br s, 1H), 2.30 (s, 3H), 2.11 - 2.05 (m, 4H), 1.31 (s, 3H). MS (ESI$^+$) *m/z* 489.2 (M+H)$^+$. HPLC purity: 97.2 %.

5-(1-(3-(4-(3-Fluorophenyl)-3-methyl-1H-pyrazol-1-yl)-2-hydroxy-2-methylpropanoyl)-4-hydroxypiperidin-4-yl)isoquinoline 2-oxide (32)

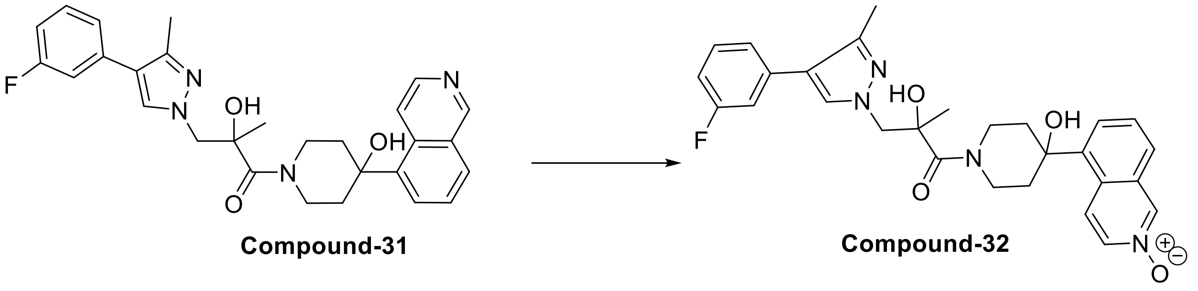

Compound **32** was prepared as described for step 3 of compound **27**; (step-3 Synthesis of tert-butyl 3-(1-(λ1-oxidanyl)-1 λ4-quinolin-5-yl)azetidine-1-carboxylate). ^{1}HNMR (400 MHz, DMSO-d_6): δ 8.91 (s, 1H), 8.80 (d, *J* = 7.2 Hz, 1H), 8.10 (d, *J* = 6.4 Hz, 1H), 7.89 (s, 1H), 7.79 - 7.76 (m, 1H), 7.55 (br s, 2H), 7.39 - 7.37 (m, 1H), 7.21 (s, 2H), 7.03 - 6.99 (m, 1H), 5.82 (s, 1H), 5.62 (s, 1H), 4.62 (br s, 1H), 4.29 (s, 3H), 3.53 (br s, 1H), 3.15 (br s, 1H), 2.26 (s, 3H), 2.07 - 2.04 (m, 5H), 1.30 (s, 3H). MS (ESI$^+$) *m/z* 505.2 (M+H)$^+$. HPLC purity: 99.9 %.

Synthesis of 2-hydroxy-1-[4-(5-isoquinolyl)piperazin-1-yl]-2-methyl-3-[(E)-3-thia-1-azatricyclo[6.3.1.04,12]dodeca-4,6,8(12)-trien-2-ylideneamino]propan-1-one (33)

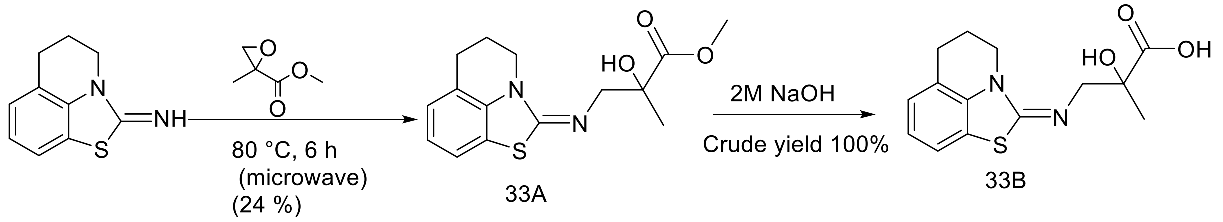

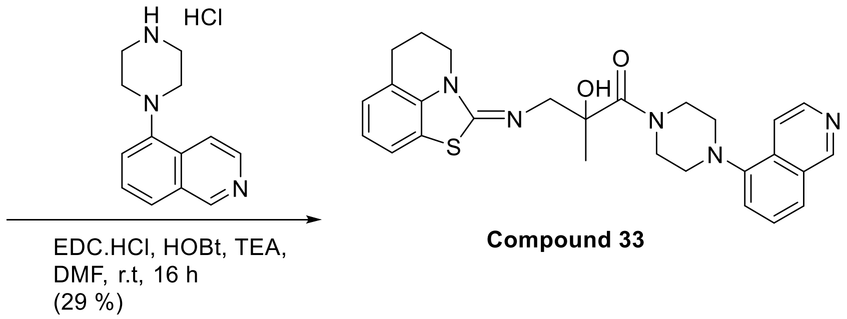

Methyl 2-hydroxy-2-methyl-3-[(E)-3-thia-1-azatricyclo[6.3.1.04,12]-dodeca-4,6,8(12) -trien-2-ylideneamino]propanoate (33A)

A solution of 3-thia-1-azatricyclo[6.3.1.04,12]dodeca-4,6,8(12)-trien-2-imine (200 mg, 1.05 mmol), methyl 2-methyloxirane-2-carboxylate (247 mg, 2.10 mmol) and potassium carbonate (293 mg, 2.10 mmol) was stirred at 80 °C in the microwave oven for 3 h. After addition of further methyl 2-methyloxirane-2-carboxylate (123 mg, 1.05 mmol) the mixture was stirred in the microwave for another 3 h at 80 °C. The reaction mixture was filtered via a glass frit, diluted with ethyl acetate and extracted twice with aqueous LiCl solution (4 % w/w). The organic layer was dried over sodium sulfate and concentrated. The crude product thus obtained was purified by preparative RP-HPLC (Column: Waters SunFire Prep C18 OBD (100mm*50mm*5µm), gradient of 0.1% TFA in water and acetonitril, Flow rate: 120 mL/min) to yield the product (**33A**, 77 mg, 24 %). ^{1}H NMR (400 MHz, DMSO-d_6): δ 7.85 (d, *J* = 7.9 Hz, 1H), 7.39 – 7.32 (m, 2H), 6.03 (s br, 1H), 4.17 – 4.06 (m, 2H), 3.75 (d, *J* = 15.0 Hz, 1H), 3.72 (s, 3H), 3.57 (d, *J* = 15.0 Hz, 1H), 2.93 – 2.90 (m, 2H), 2.16 – 2.09 (m, 2H), 1.40 (s, 3H). MS (ESI$^+$) *m/z* 307.2 (M+H)$^+$.

2-Hydroxy-2-methyl-3-[(E)-3-thia-1-azatricyclo[6.3.1.04,12]dodeca-4,6,8(12)-trien-2-ylideneamino]propanoic acid (33B)

To a solution of methyl 2-hydroxy-2-methyl-3-[(E)-3-thia-1-azatricyclo[6.3.1.04,12]dodeca-4,6,8(12)-trien-2-ylideneamino]propanoate (**33A**, 66 mg, 0.215 mmol) was added 2 M sodium hydroxide solution (389 µl, 0.778 mmol). After stirring for 45 minutes the reaction mixture was evaporated to give the title compound (**33B**, 63 mg, 100 %) as its sodium salt which was used in the next step without further purification. An analytically pure sample was obtained by purification via RP-HPLC (Column: Waters SunFire Prep C18 OBD (100mm*50mm*5µm), gradient of 0.1% TFA in water and acetonitril, Flow rate: 120 mL/min). ^{1}H NMR (400 MHz, DMSO-d_6): δ 7.82 (d, *J* = 8.8 Hz, 1H), 7.36 – 7.29 (m, 2H), 5.79 (s br, 1H), 4.18 – 4.09 (m, 2H), 3.72 (d, *J* = 15 Hz, 1H), 3.54 (d, *J* = 15 Hz, 1H), 2.92 - 2.90 (m, 2H), 2.16-2.10 (m, 2H), 1.39 (s, 3H). MS (ESI$^+$) *m/z* 293.1 [M+H]$^+$.

2-Hydroxy-1-[4-(5-isoquinolyl)piperazin-1-yl]-2-methyl-3-[(E)-3-thia-1-azatri-cyclo[6.3.1.04,12]dodeca-4,6,8(12)-trien-2-ylideneamino]propan-1-one 2,2,2-trifluoroacetate (33)

To a solution of 2-hydroxy-2-methyl-3-[(E)-3-thia-1-azatricyclo[6.3.1.04,12]dodeca-4,6,8(12)-trien-2-ylideneamino]propanoic acid (**33B**, 663 mg, 0.215 mmol) were added subsequently HOBt (44 mg,

0.280 mmol), EDC (55 mg, 0.280 mmol) and N,N-diisopropylethylamine (84 µl, 0.646 mmol) in 3 mL DMF. After stirring for 30 minutes at room temperature 5-piperazin-1-ylisoquinoline hydrochloride (54 mg, 0.215 mmol) was added and the mixture was stirred for 16 h. The reaction mixture was diluted with ethyl acetate and extracted twice with aqueous LiCl solution (4 % w/w). The organic layer was dried over sodium sulfate and concentrated. The crude product thus obtained was purified by preparative RP-HPLC to yield the product (**33**, 38 mg, 29 %) as TFA salt. ^{1}H NMR (600 MHz, DMSO-d_6): δ 9.79 (br s, 1H), 8.70 (br s, 1H), 8.25 (br s, 1 H), 7.96 (d, *J* = 8.5 Hz, 1H), 7.83 (br d, *J* = 7.6 Hz, 1H), 7.74 (t, *J* = 7.8 Hz, 1H), 7.51 (d, *J* = 7.4 Hz, 1H), 7.40 – 7.34 (m, 2H), 6.25 (br s, 1H), 4.31 – 4.14 (m, 4H), 3.91 – 3.72 (m, 2H), 3.81 (d, *J* = 14.0 Hz, 1H), 3.67 (d, *J* = 14.0 Hz, 1H), 3.10 (m, 4H), 2.92 (br t, *J* = 5.9 Hz, 2H), 2.20 – 2.10 (m, 2H), 1.52 (s, 3H); ^{13}C NMR (150.9 MHz, DMSO-d_6) δ 172.0, 171.1, 148.1, 135.4, 135.3, 131.4, 128.8, 126.4, 125.4, 124.6, 123.4, 121.0, 120.8, 120.7, 76.3, 57.6, 52.5, 45.3, 23.7, 23.5, 20.4 (4 carbon atoms not found); HRMS (ESI$^+$): calcd for $C_{27}H_{29}N_5O_2S$ $(M+H)^+$: 488.2115; Found: 488.2110.

Experimental Procedures S2: Fluorescence-based NNMT enzymatic assay

NNMT activity was measured by fluorescence based assay. The product MNA formed in the reaction reacts with acetophenone in the presence of KOH and formic acid and forms a fluorescent product 2, 7- naphthyridine. The fluorescence intensity measured with excitation at 375nm and emission at 430nm. Inhibitors were screened using human, mouse and monkey NNMT enzymes. Different concentrations of inhibitors were pre-incubated along with 5ng/well of human NNMT enzyme and 3.42ng/well of mouse NNMT enzyme for 30 minutes at room temperature. Reaction was initiated by addition of SAM and nicotinamide mixture (7µM, 20µM, 8µM SAM and 6µM, 20µM, 9µM nicotinamide for human, mouse and monkey NNMT assays respectively) and incubated for 60 minutes at 37°C. The final assay reaction mixture contained a buffer of 100mM Tris Hcl pH 7.5, 0.04% BSA, 2mM Dithiothreitol and 1% DMSO. The reaction was terminated by addition of Ethanol: Acetophenone mix (75% ethanol: 25% Acetophenone) and 5M potassium Hydroxide prepared with 50% Ethanol. The reaction was incubated for 15 minutes followed by addition of 100µl of 60% formic acid and further incubation for 60 minutes at room temperature. A fluorescent product 2, 7- naphthyridine was measured in a Tecan reader with excitation at 375nm and emission at 430nm. The IC_{50} values were determined by fitting the inhibition curves (percent inhibition versus inhibitor concentration) using a four parametric sigmoidal dose response in graph pad prism.

Experimental Procedures S3: Metabolic stability in human and mouse liver microsomes

These profiling studies were carried out as described in Mullangi R. et. al. *Preclinical assessment of ulixertinib, a novel ERK1/2 inhibitor*, *ADMET & DMPK*, **2017**, 212-223; **http://dx.doi.org/10.5599/admet.5.4.437**

Figure S1: High-resolution MS and NMR spectra of **(1)**

^{1}H NMR of compound 1:

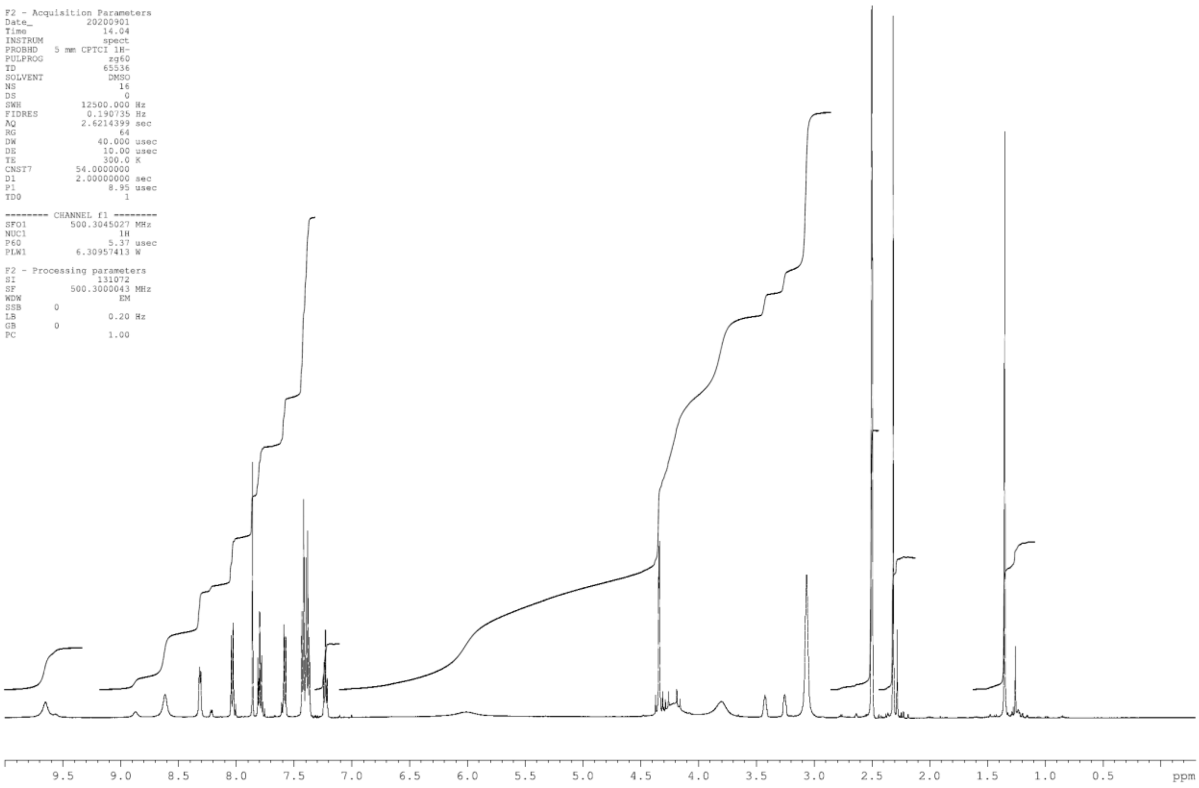

^{13}C NMR of compound 1:

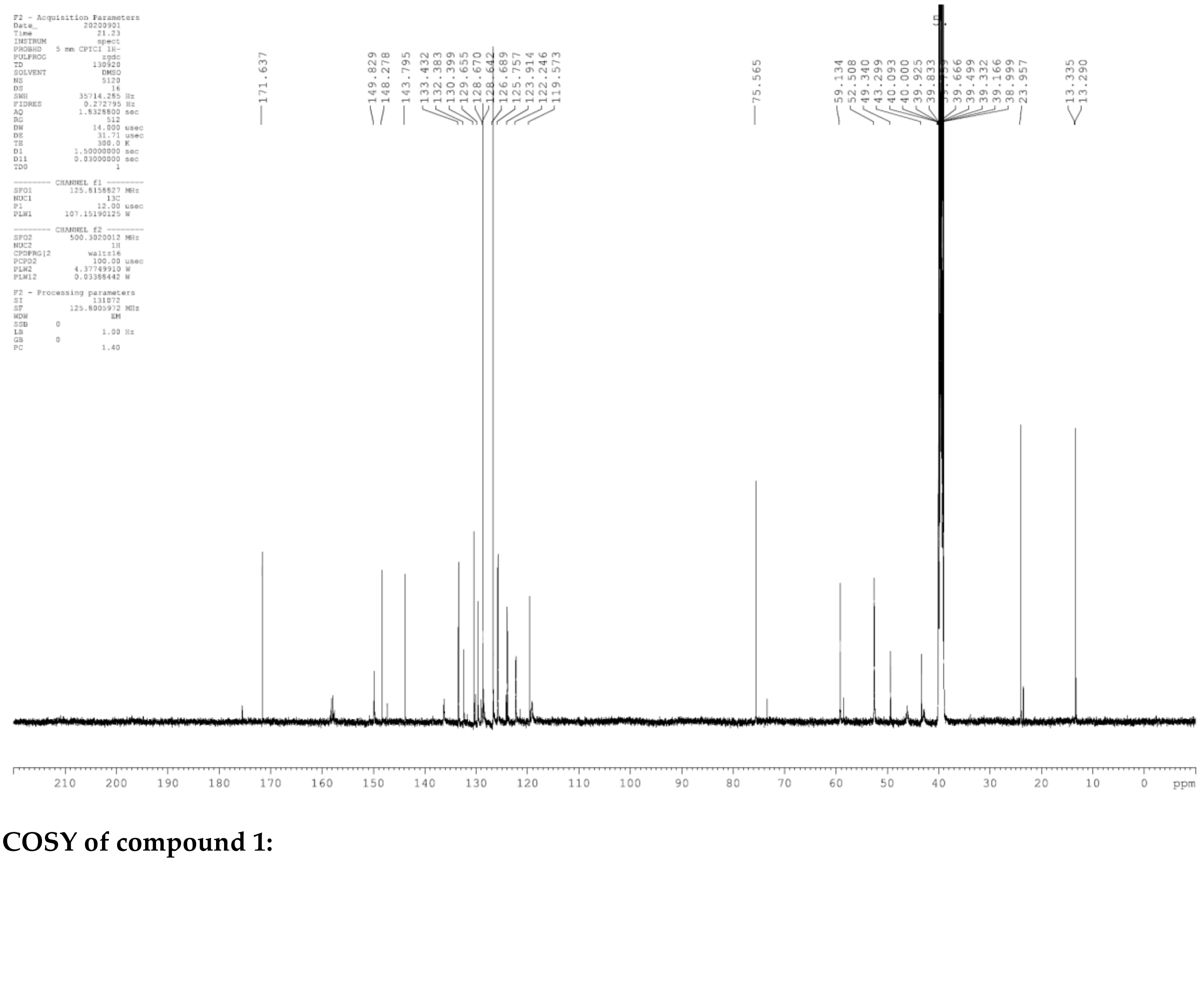

COSY of compound 1:

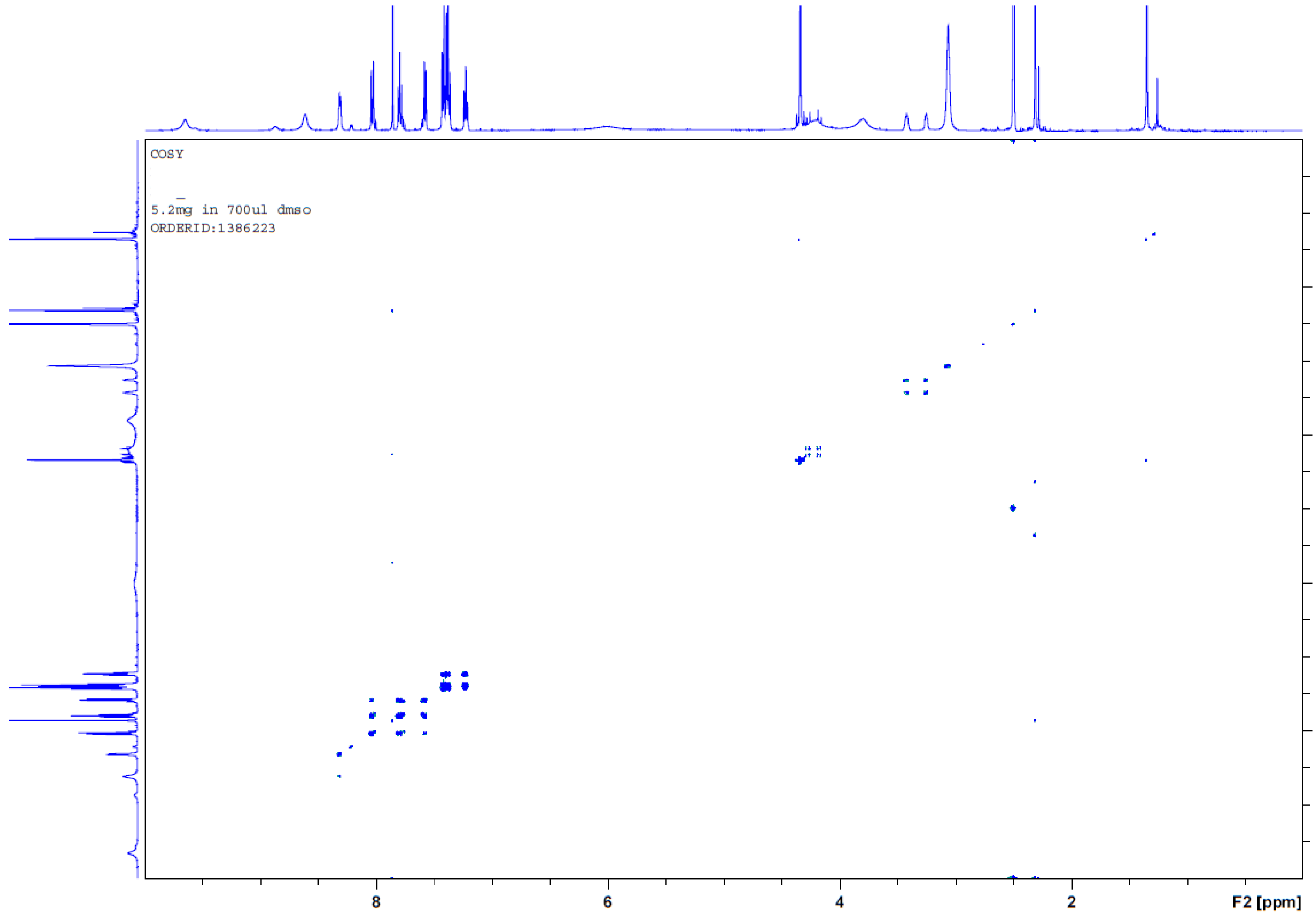
COSY
5.2mg in 700ul dmso
ORDERID:1386223
8
6
4
2
F2 [ppm]

HSQC of compound 1:

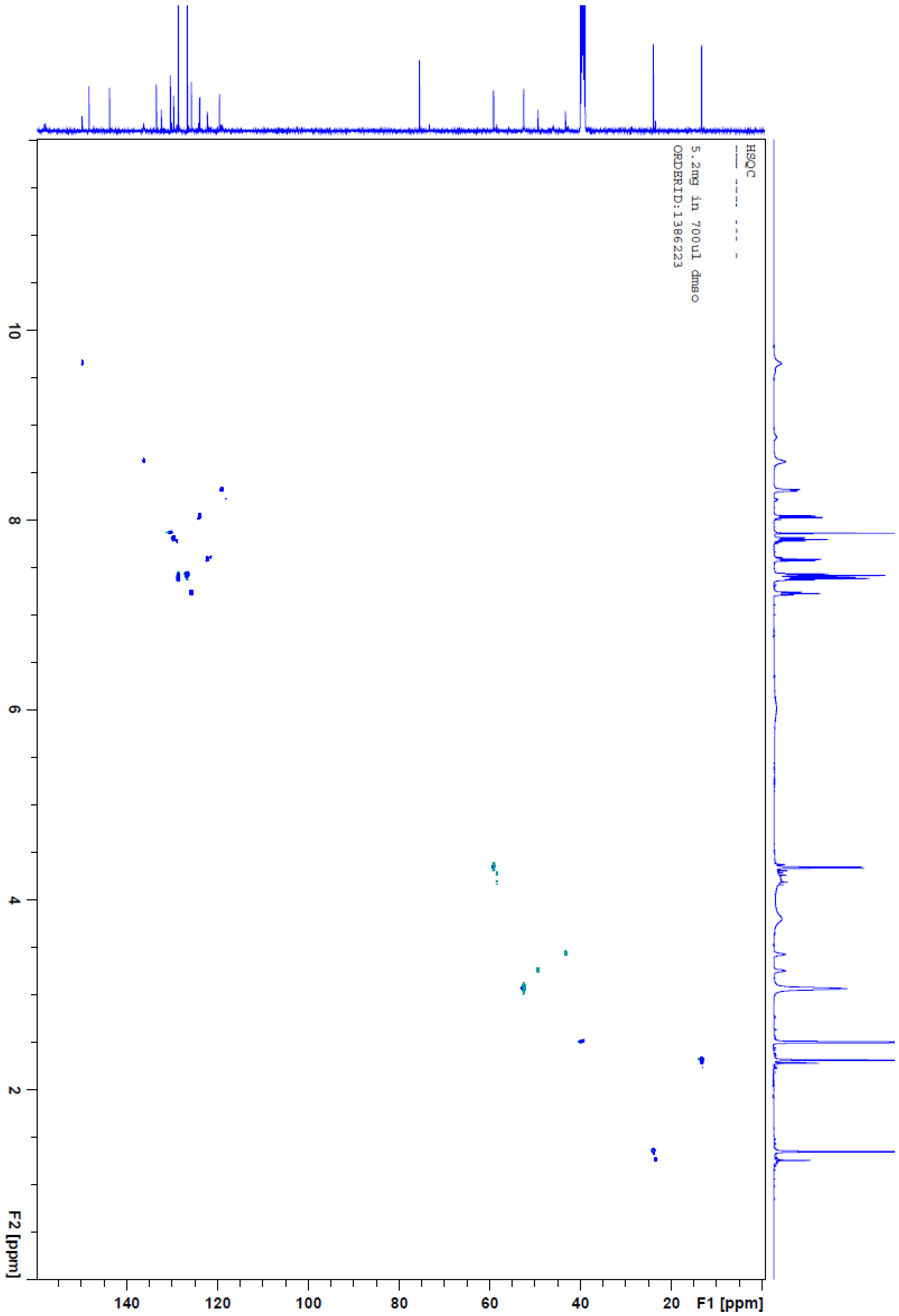

HRMS (ESI+) compound 1:

Compound Label	*m/z*	RT	Algorithm	Mass
Cpd 1: C27 H29 N5 O2	456.2388	1.889	Find By Formula	455.2315

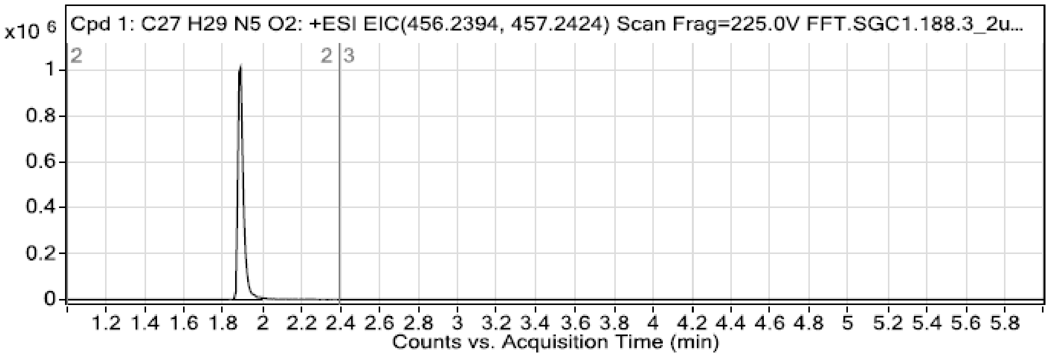

MS Spectrum

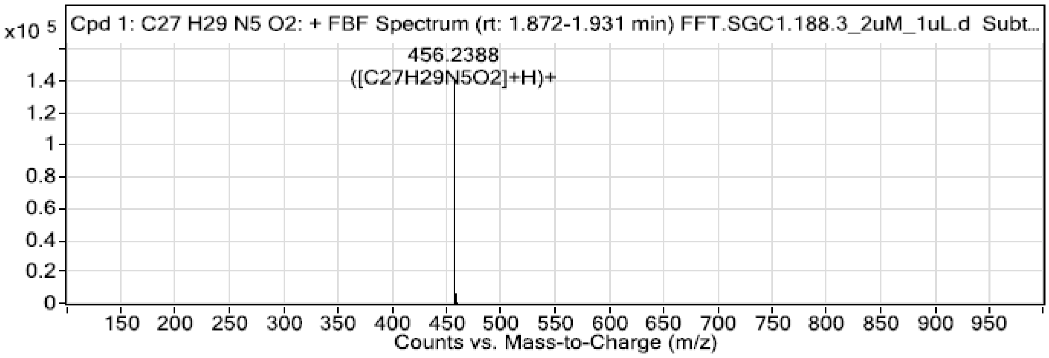

MS Zoomed Spectrum

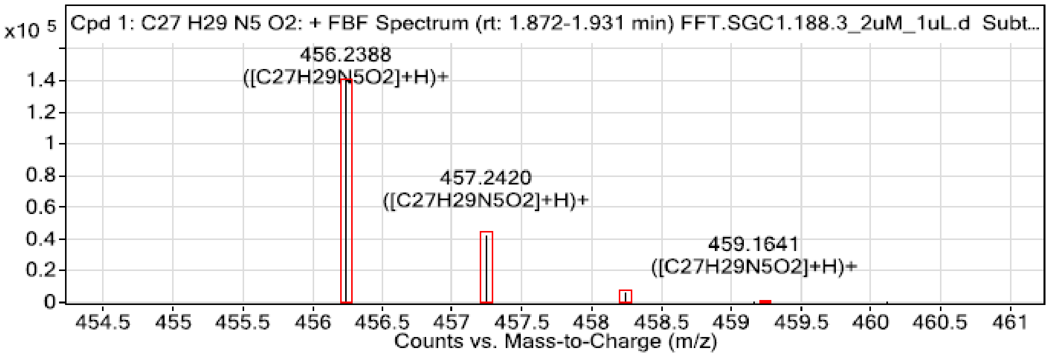

MS Spectrum Peak List

m/z	*Calc m/z*	Diff(ppm)	z	Abund	Formula	Ion
456.2388	456.2394	1.33	1	140867.43	C27H29N5O2	(M+H)+
457.242	457.2424	0.87	1	41943.51	C27H29N5O2	(M+H)+
458.245	458.2453	0.53	1	6309.06	C27H29N5O2	(M+H)+
459.1641	459.248	182.89	1	130.58	C27H29N5O2	(M+H)+
460.1148	460.2507	295.38	1	74.99	C27H29N5O2	(M+H)+

Figure S2: High-resolution MS and NMR spectra of **(2)**

^{1}H NMR of compound 2:

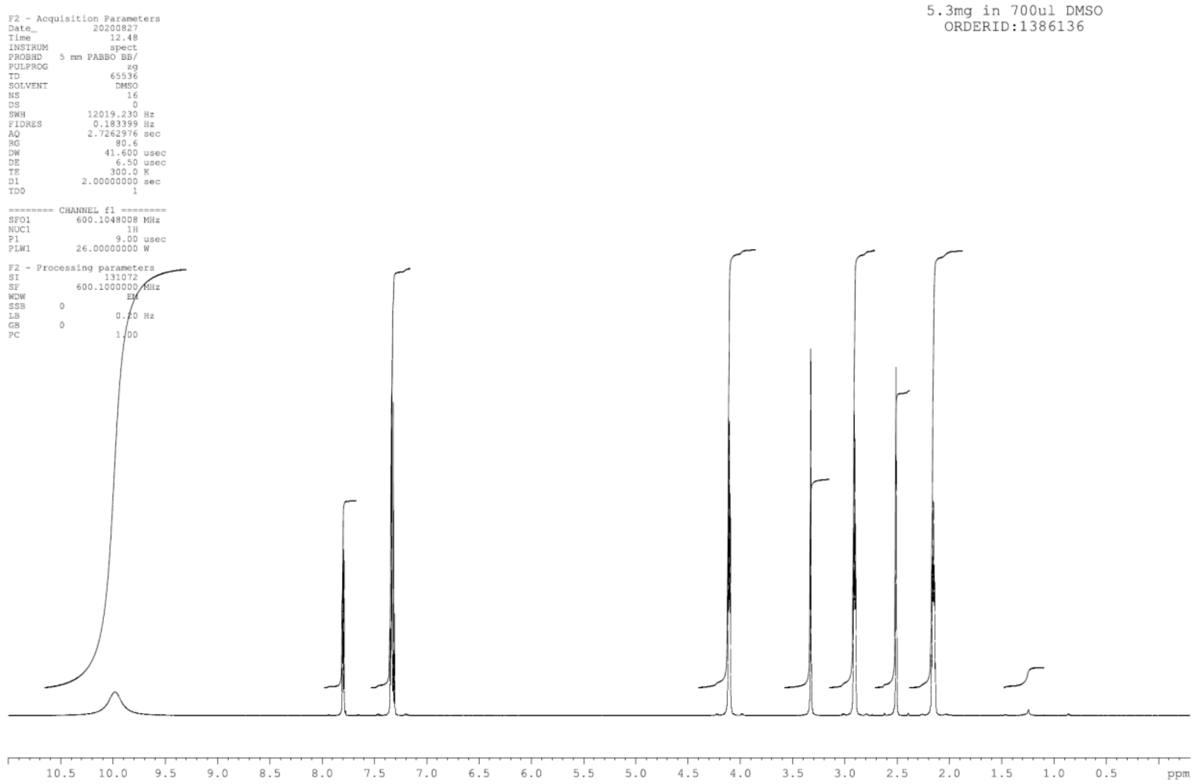

^{13}C NMR of compound 2:

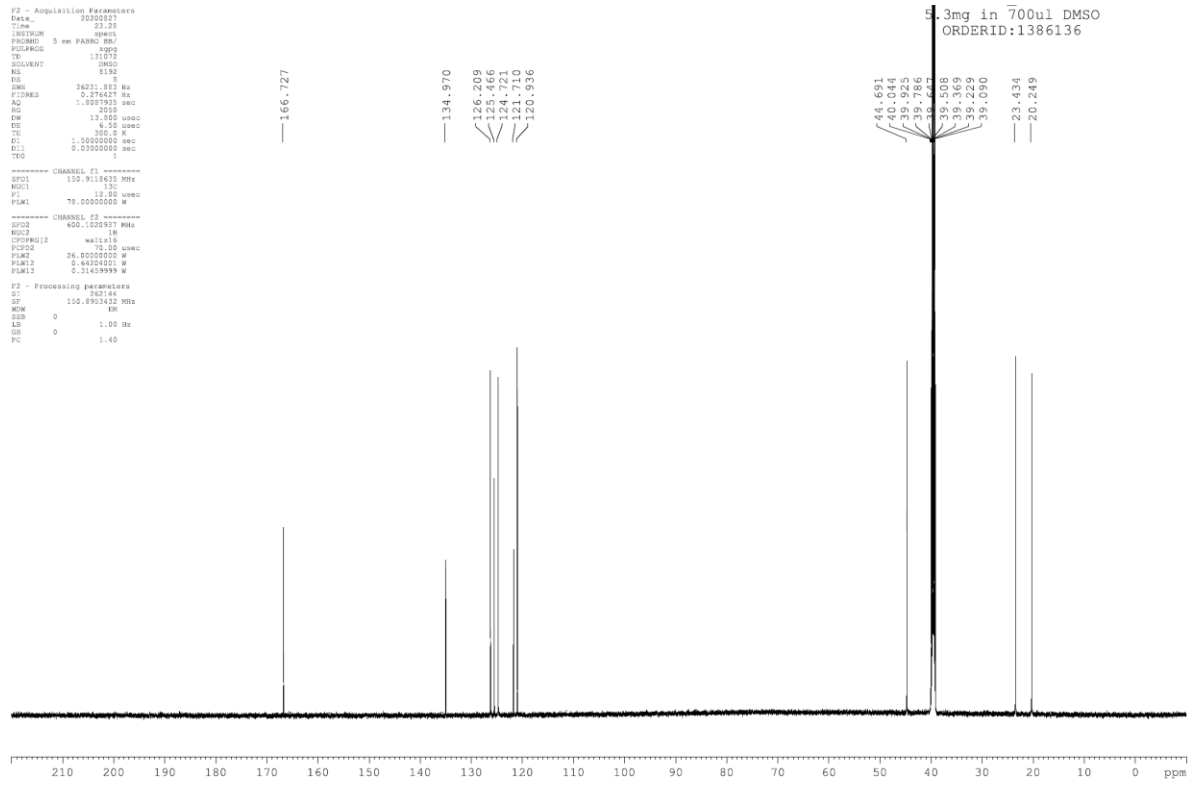

COSY of compound 2:

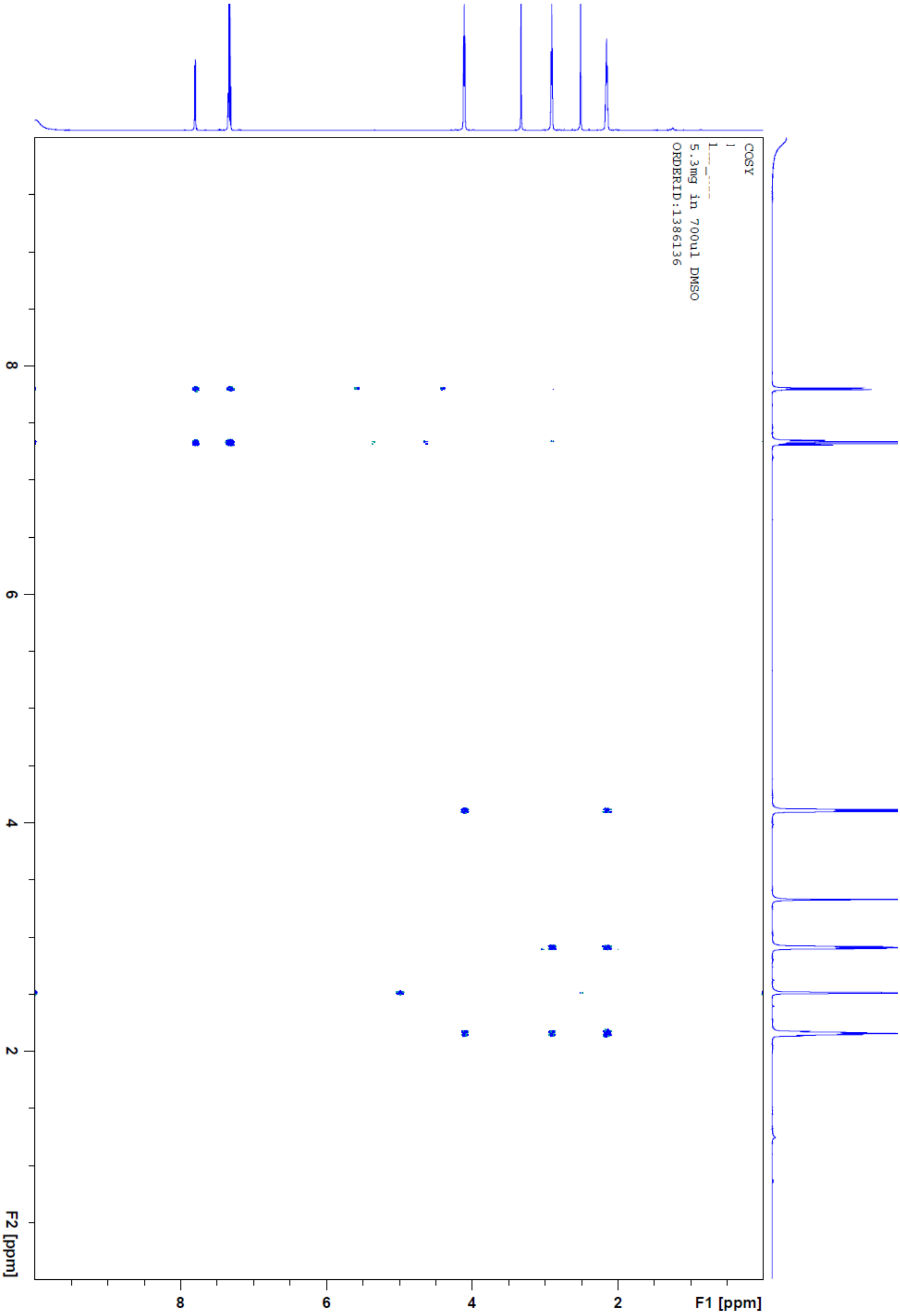

HSQC of compound 2:

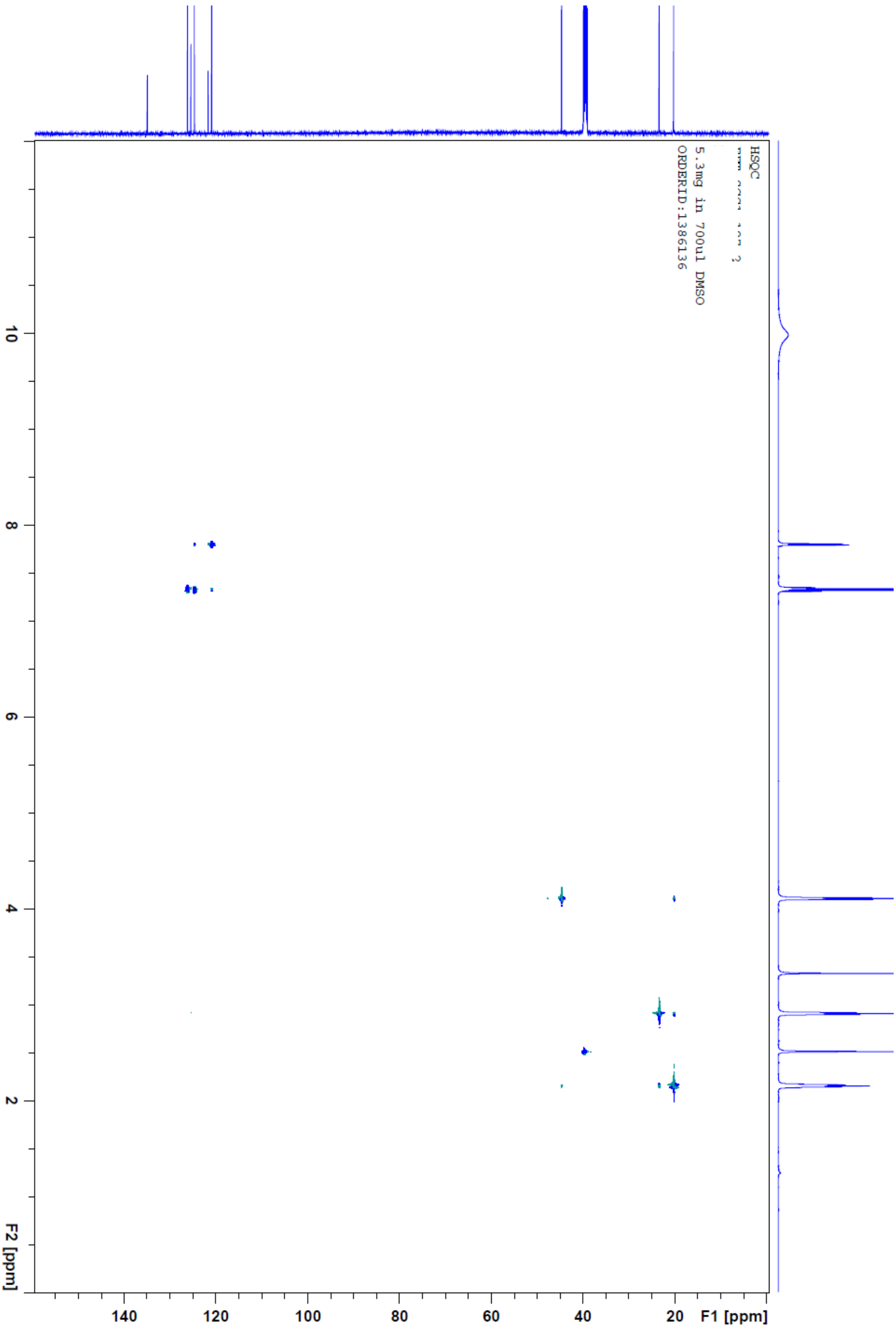

HRMS (ESI+) compound 2:

Compound Label	*m/z*	RT	Algorithm	Mass
Cpd 1: C10 H10 N2 S	191.0641	1.654	Find By Formula	190.0568

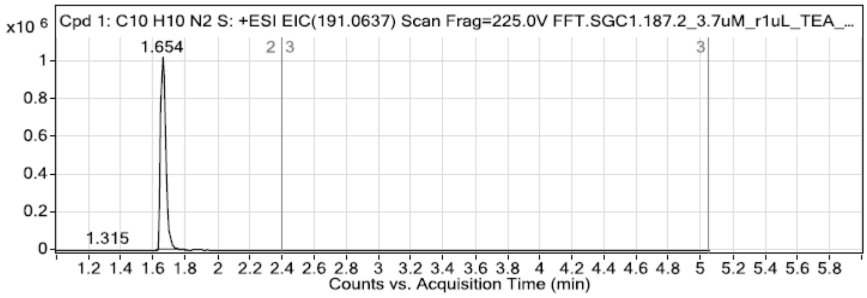

MS Spectrum

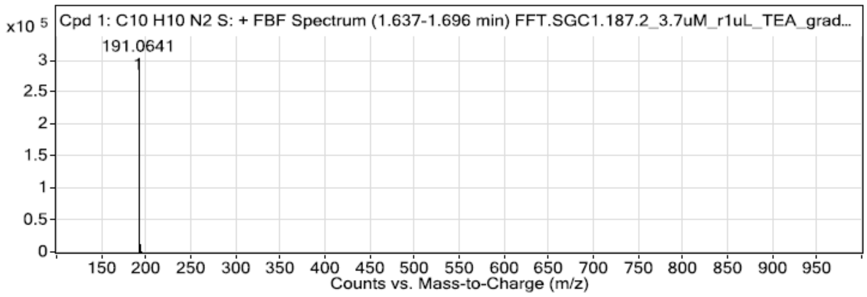

MS Zoomed Spectrum

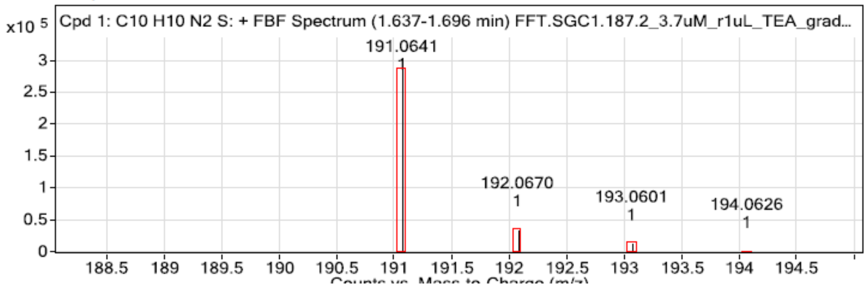

MS Spectrum Peak List

m/z	*Calc m/z*	Diff(ppm)	z	Abund	Formula	Ion
191.0641	191.0637	-1.79	1	289221.68	C10H10N2S	(M+H)+
192.067	192.0665	-2.78	1	35167.43	C10H10N2S	(M+H)+
193.0601	193.0609	3.93	1	13417.66	C10H10N2S	(M+H)+
194.0626	194.063	1.63	1	1593.92	C10H10N2S	(M+H)+
195.0635	195.0638	1.49	1	157.18	C10H10N2S	(M+H)+

Figure S3: High-resolution MS and NMR spectra of **(3)**

^{1}H NMR of compound 3:

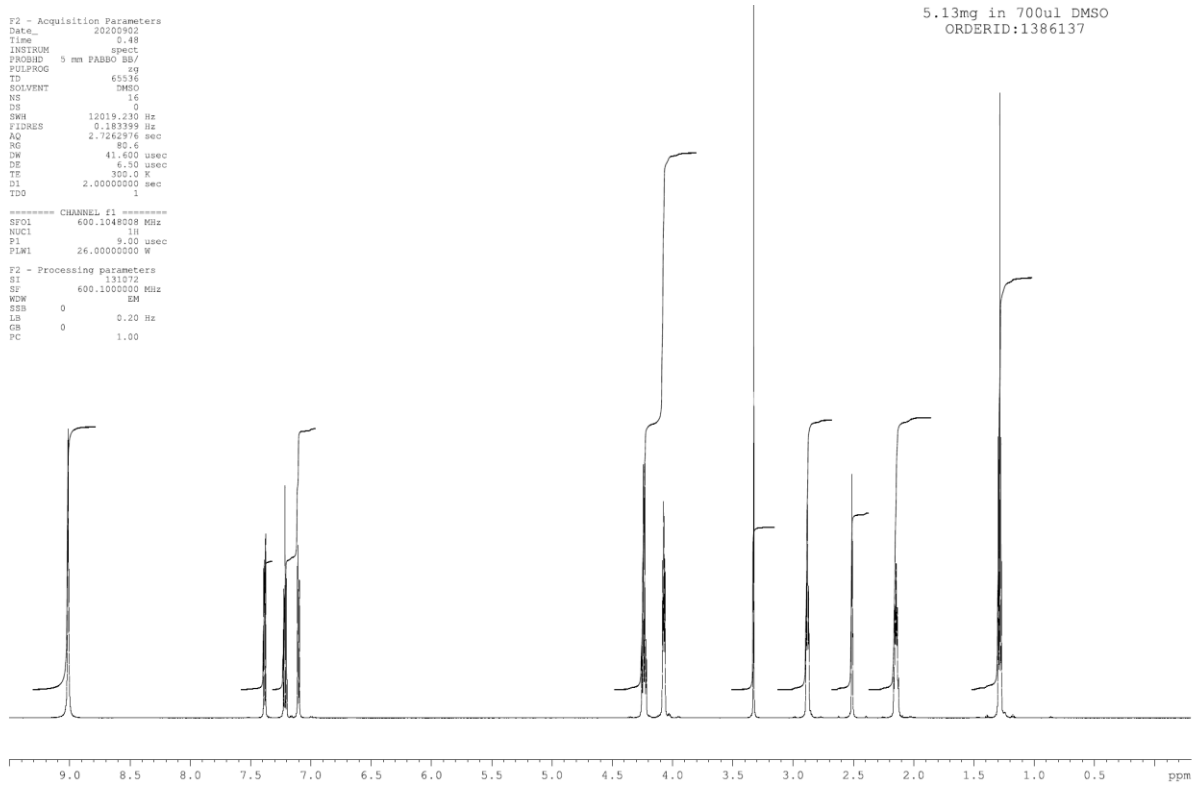

^{13}C NMR of compound 3:

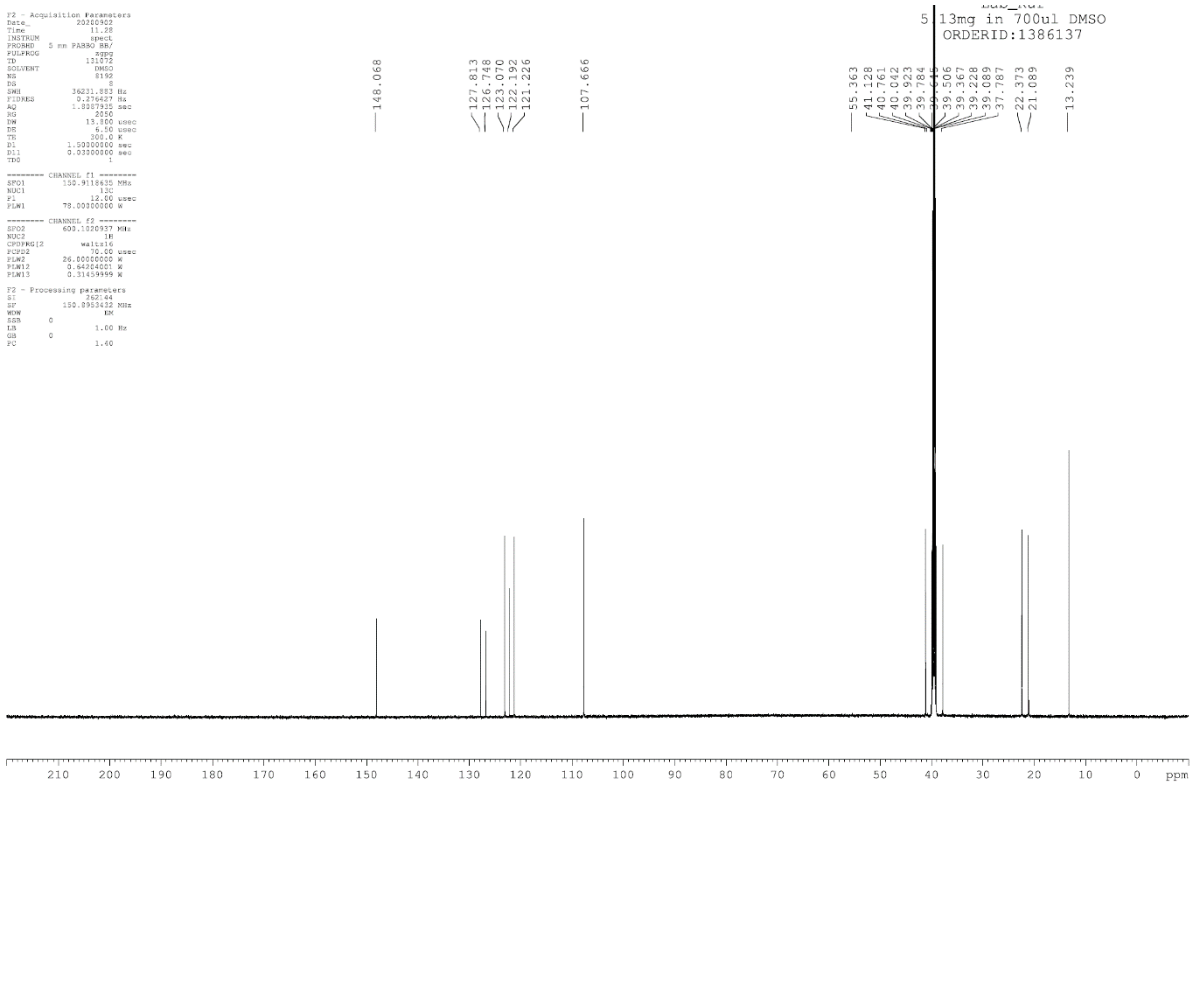

COSY of compound 3:

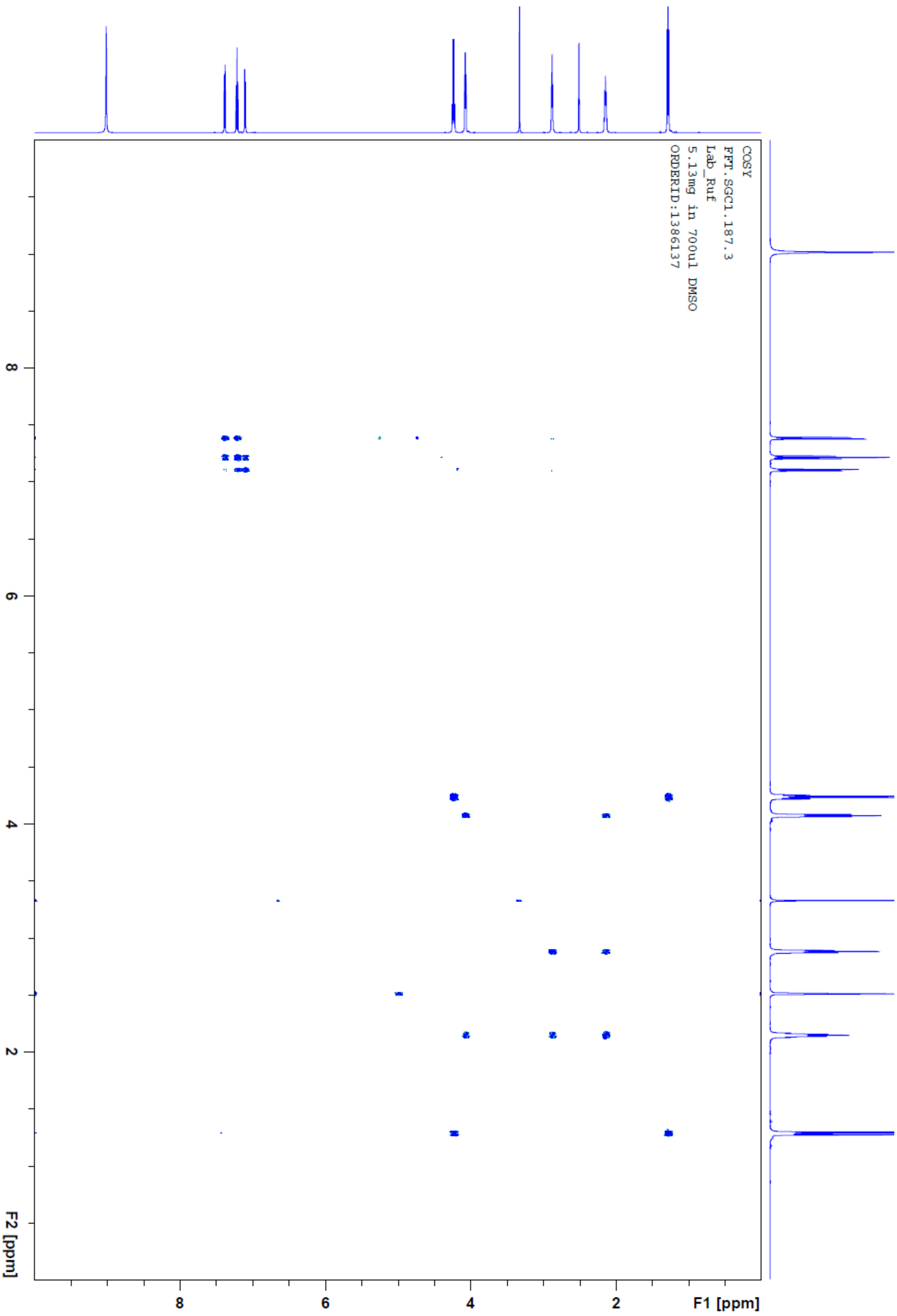

HSQC of compound 3:

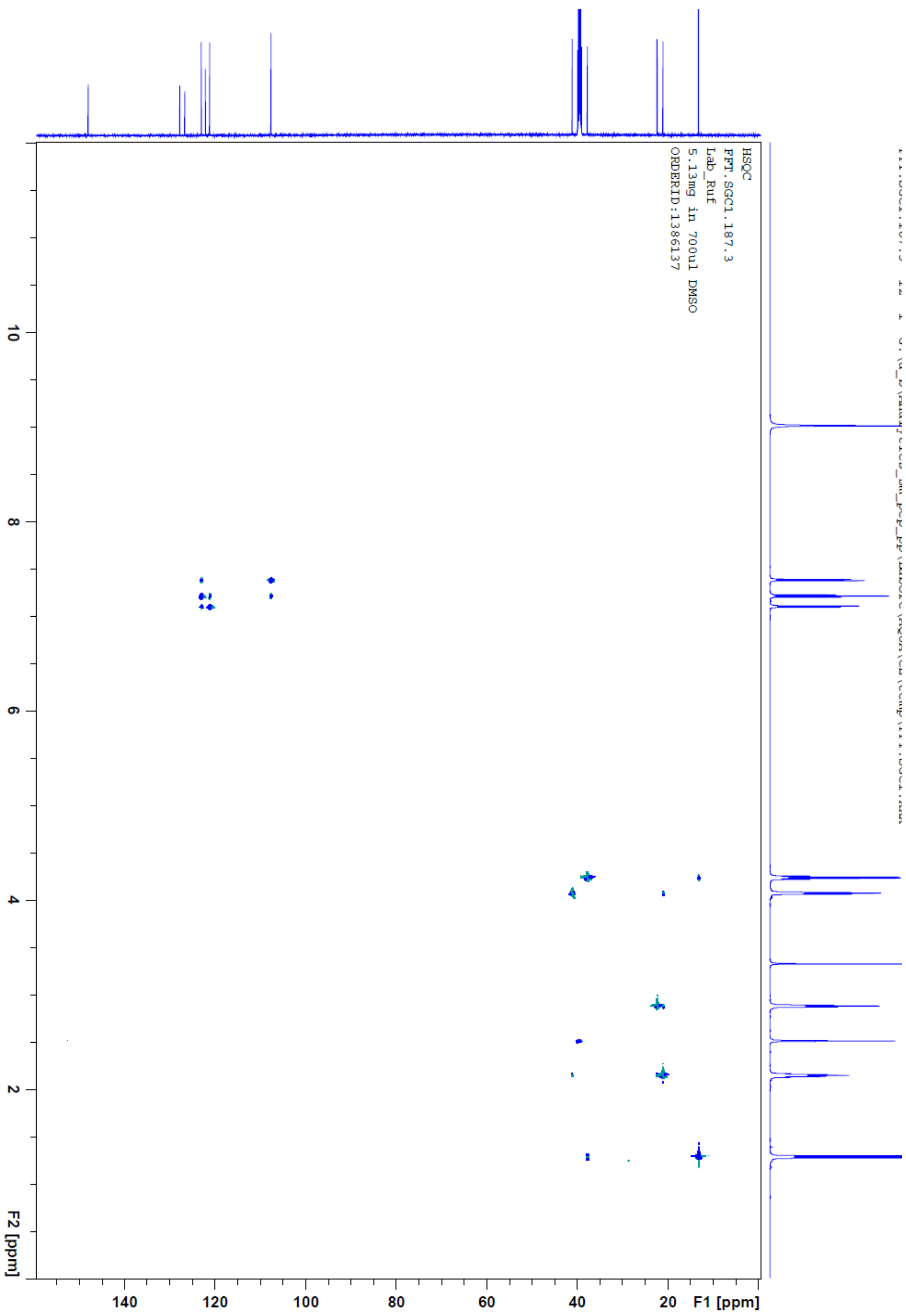

HRMS (ESI+) compound 3:

Compound Label	*m/z*	RT	Algorithm	Mass
Cpd 1: C12 H15 N3	202.134	1.68	Find By Formula	201.1267

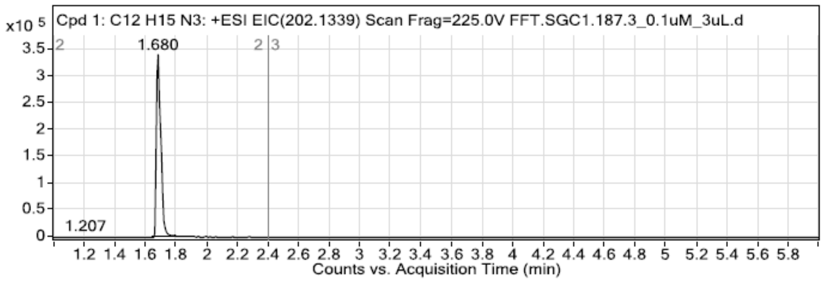

MS Spectrum

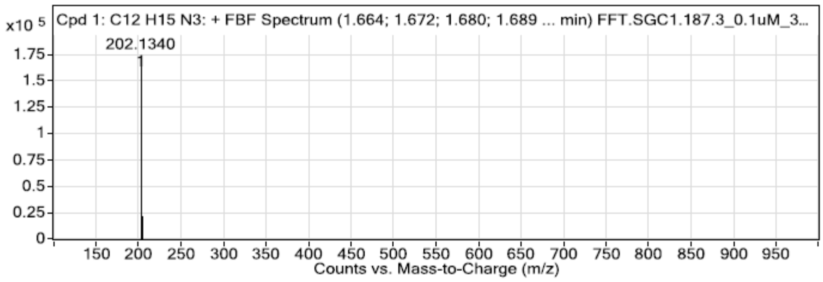

MS Zoomed Spectrum

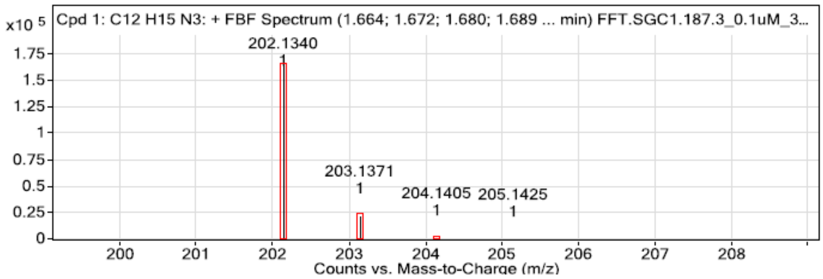

MS Spectrum Peak List

m/z	*Calc m/z*	Diff(ppm)	z	Abund	Ion
102.1287				905537.38	
202.134	202.1339	-0.43	1	166214.25	(M+H)+
203.1371	203.1368	-1.42	1	22168.12	(M+H)+
204.1405	204.1396	-4.16	1	1347.5	(M+H)+
205.1425	205.1425	0.02	1	111.06	(M+H)+

Figure S4: High-resolution MS and NMR spectra of **(4)**

^{1}H NMR of compound 4:

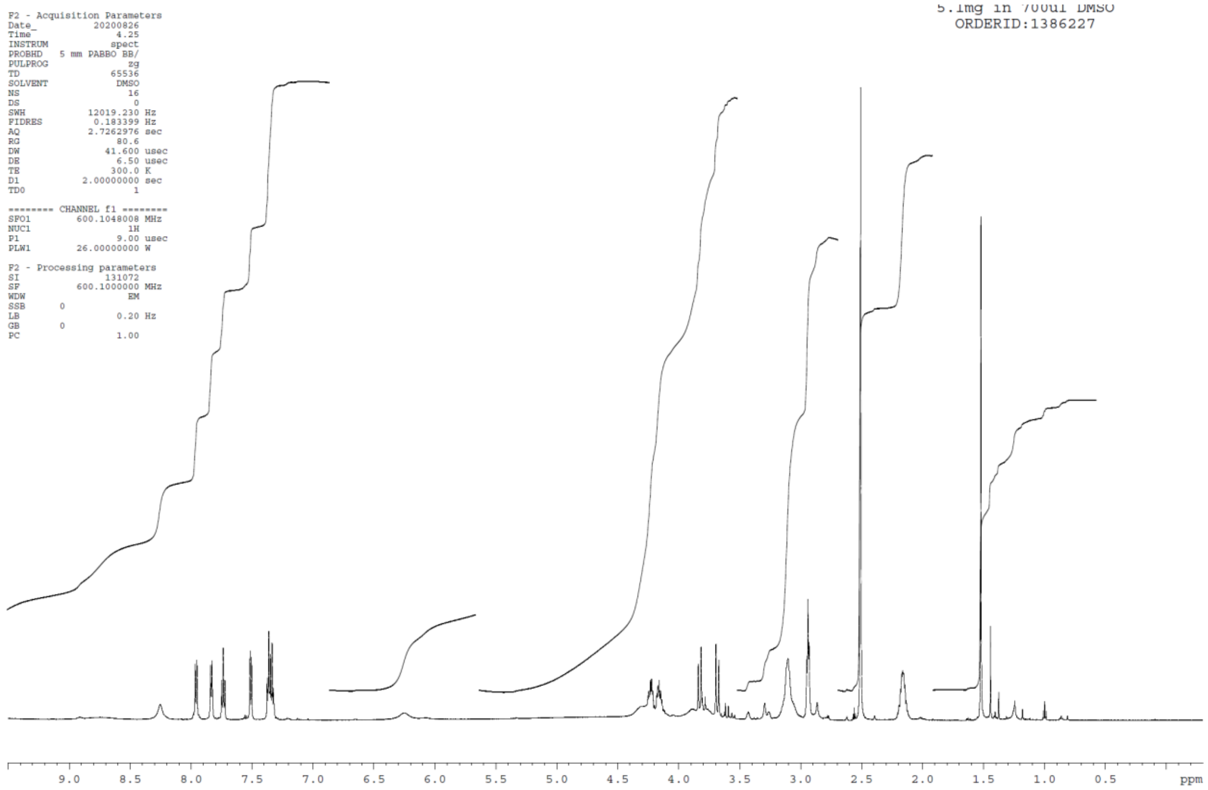

^{13}C NMR of compound 4:

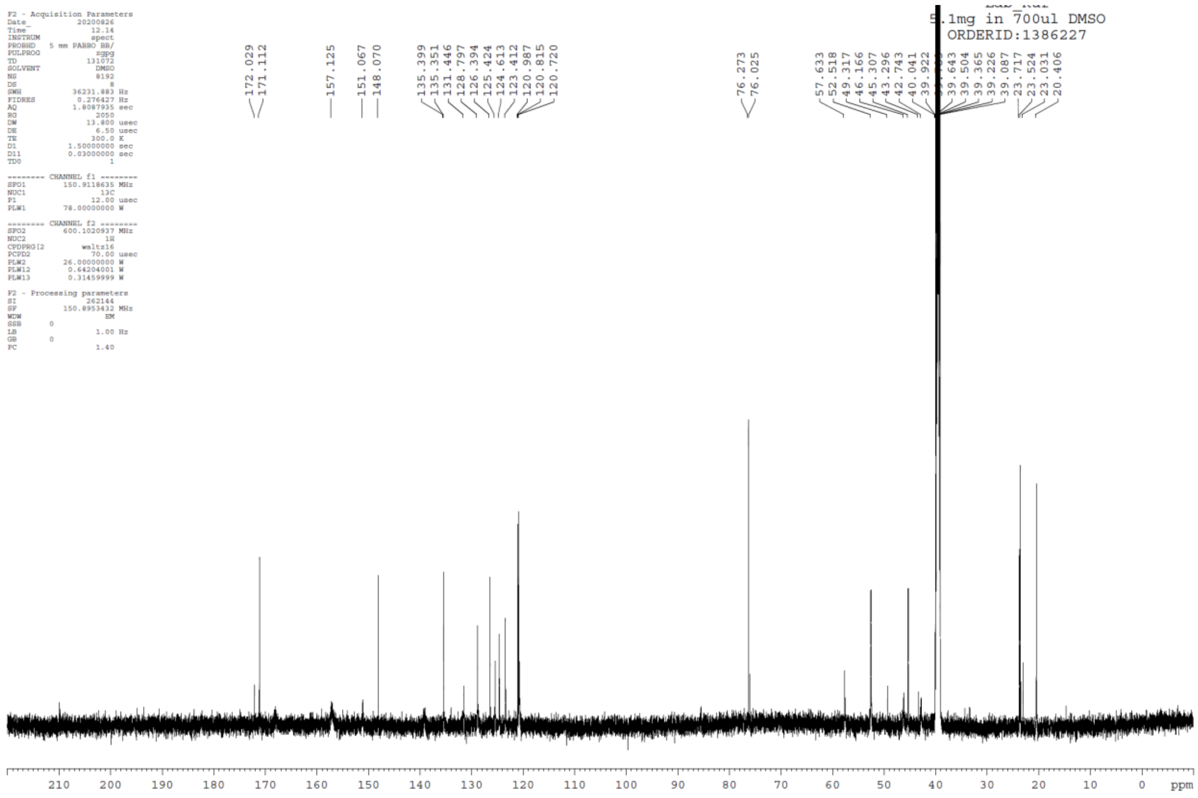

COSY of compound 4:

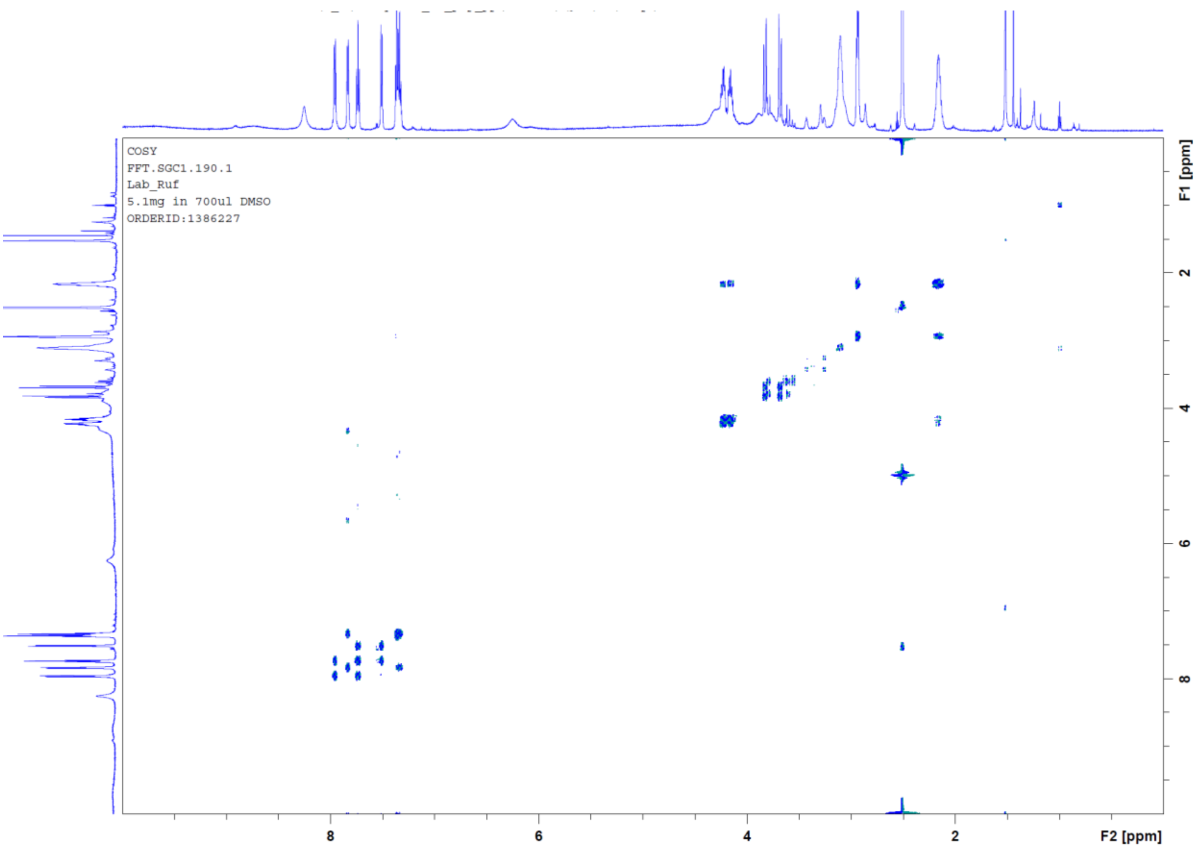

HSQC of compound 4:

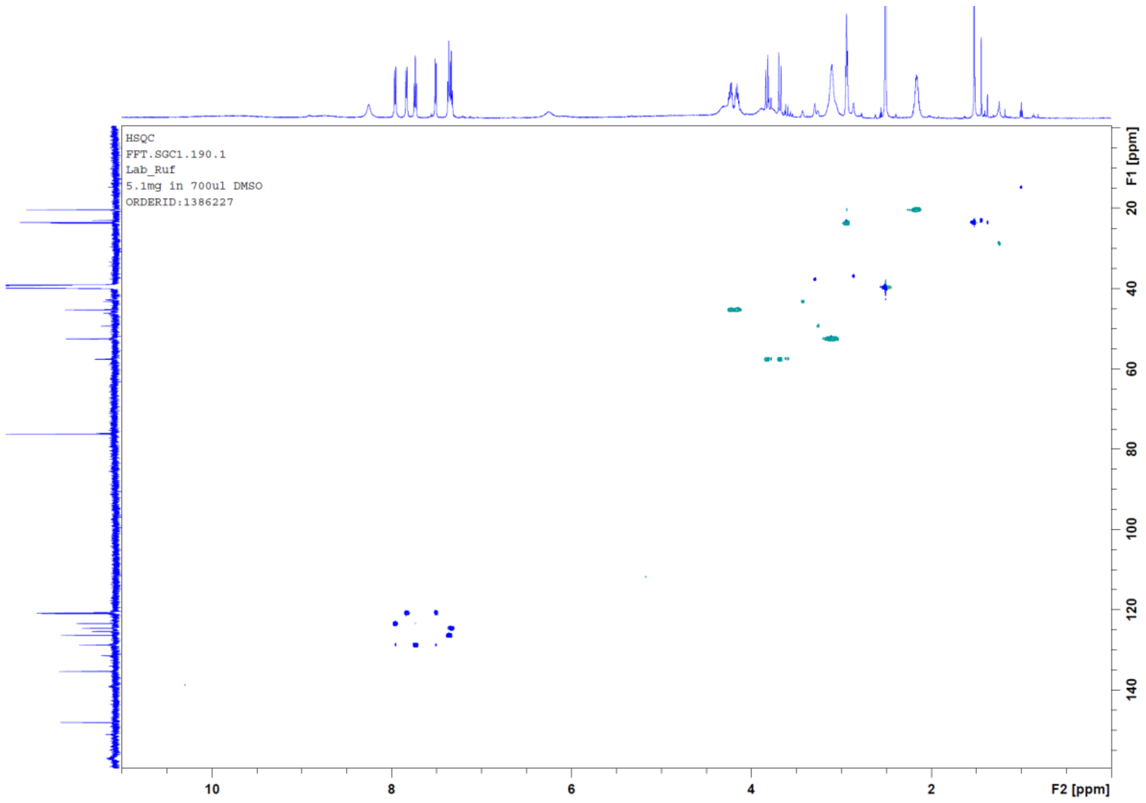

HRMS (ESI+) compound 4:

Compound Label	*m/z*	RT	Algorithm	Mass
Cpd 1: C12 H15 N3	202.1339	1.646	Find By Formula	201.1267

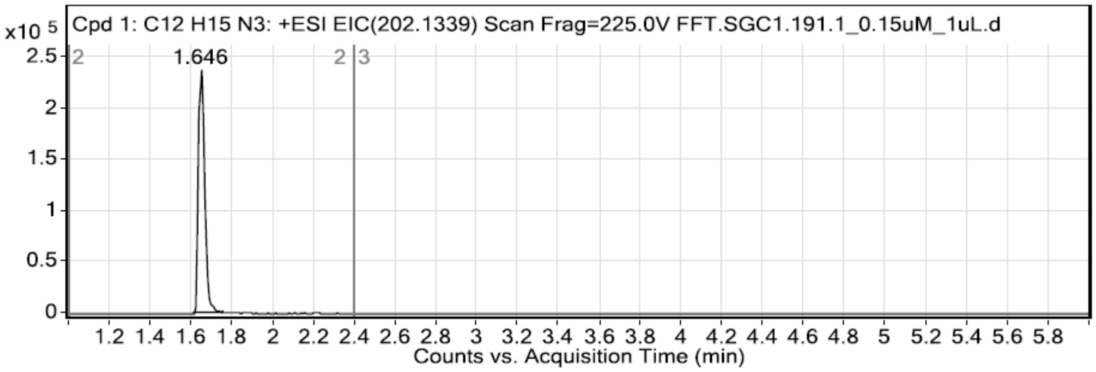

MS Spectrum

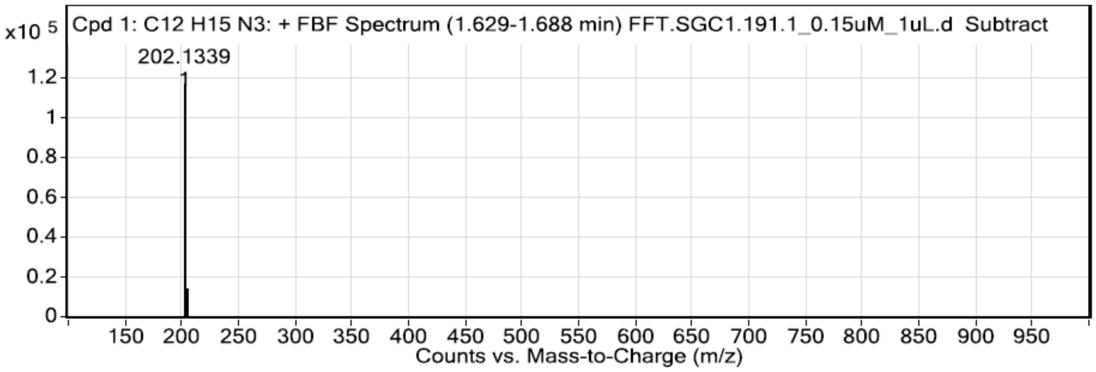

MS Zoomed Spectrum

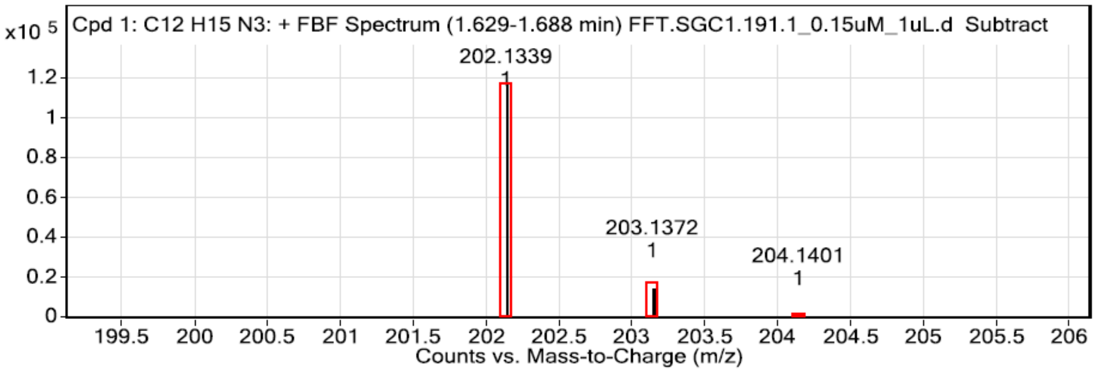

MS Spectrum Peak List

m/z	*Calc m/z*	Diff(ppm)	z	Abund	Formula	Ion
102.1278				220171.9		
202.1339	202.1339	-0.15	1	117762.32	C12H15N3	(M+H)+
203.1372	203.1368	-2.08	1	14988.87	C12H15N3	(M+H)+
204.1401	204.1396	-2.22	1	940.31	C12H15N3	(M+H)+

Figure S5: High-resolution MS and NMR spectra of **(33)**

^{1}H NMR of compound 33:

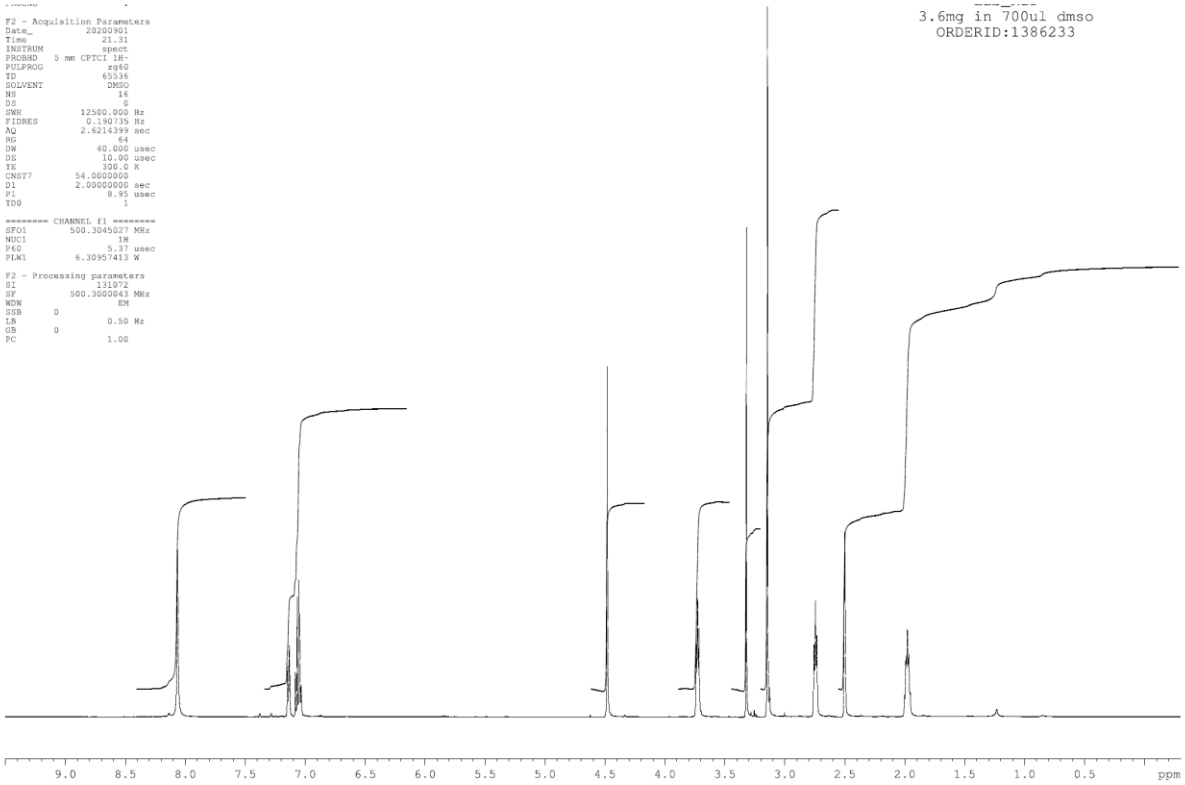

^{13}C NMR of compound 33:

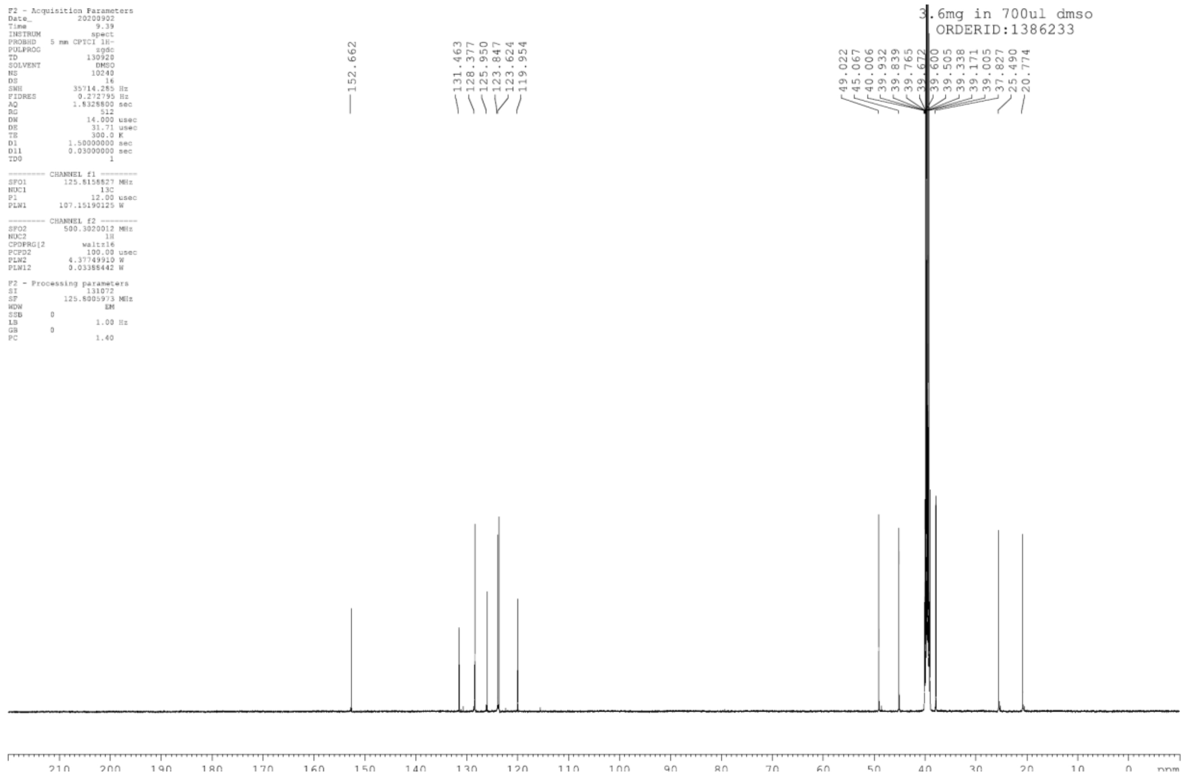

COSY of compound 33:

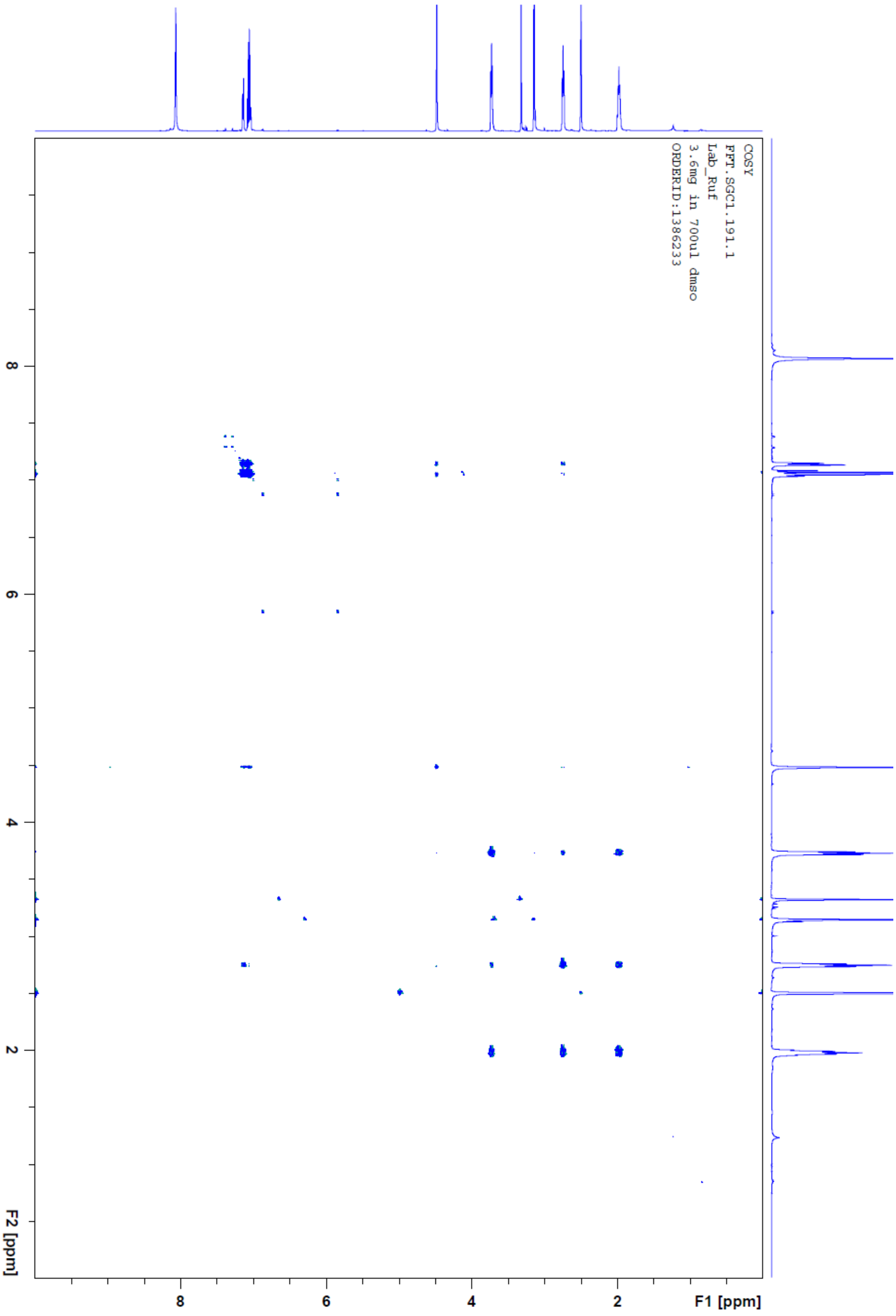

HSQC of compound 33:

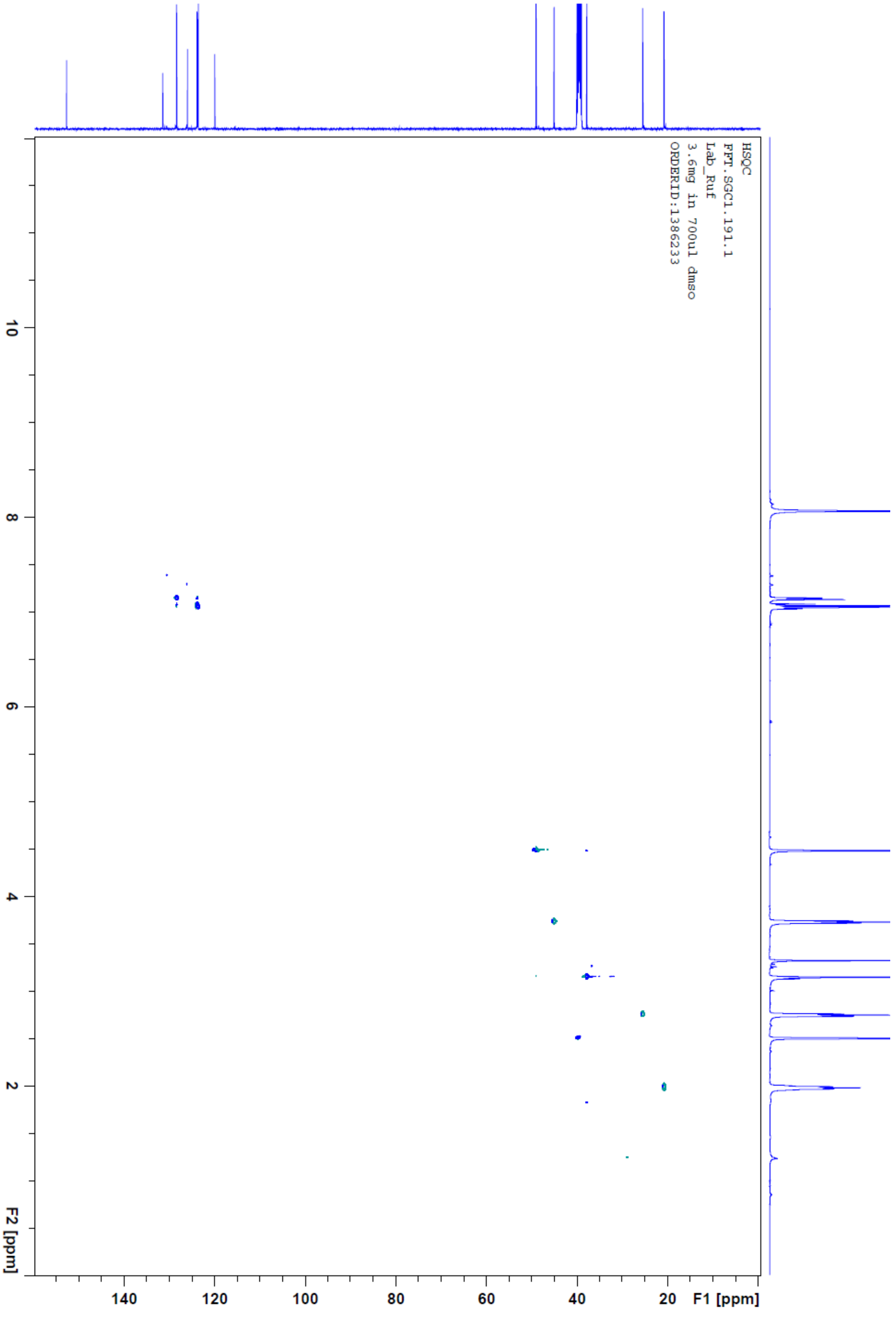

HRMS (ESI+) compound 33:

Compound Label	*m/z*	RT	Algorithm	Mass
Cpd 1: C27 H29 N5 O2 S	488.211	1.675	Find By Formula	487.2038

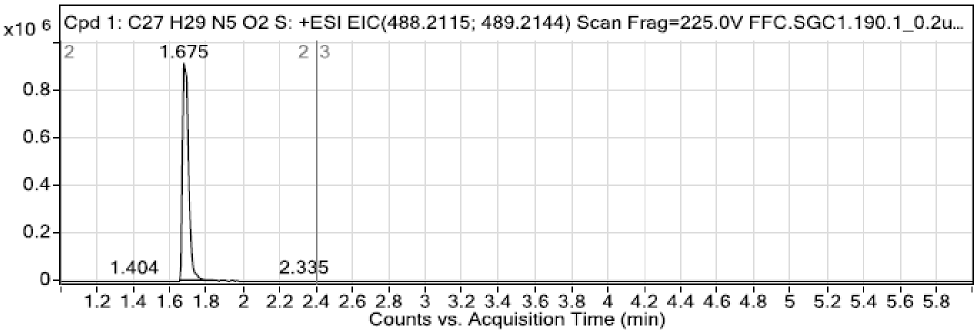

MS Spectrum

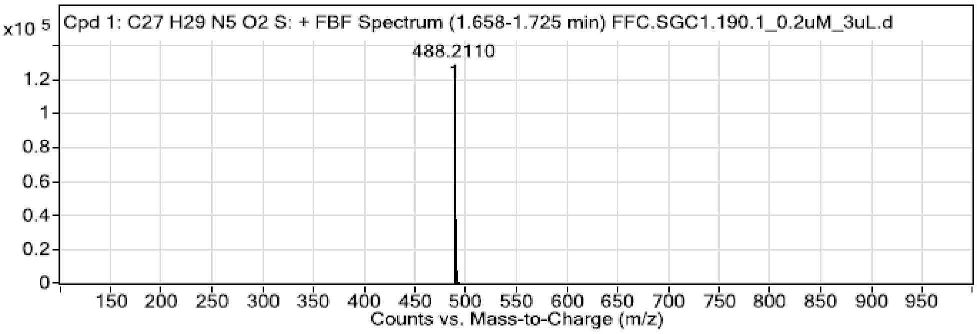

MS Zoomed Spectrum

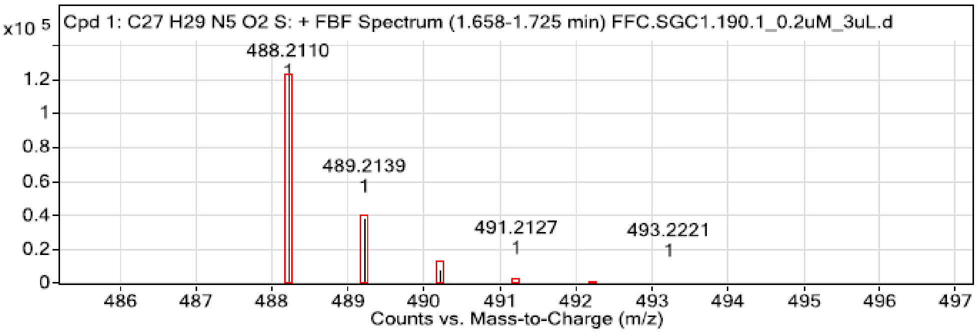

MS Spectrum Peak List

m/z	*Calc m/z*	Diff(ppm)	z	Abund	Ion
488.211	488.2115	1	1	123114.51	(M+H)+
488.211				123114.51	
489.2139	489.2144	1.06	1	37997.19	(M+H)+
490.2132	490.2127	-0.94	1	8461.2	(M+H)+
491.2127	491.2133	1.16	1	1674.39	(M+H)+
492.2155	492.2147	-1.52	1	357.45	(M+H)+
493.2221	493.2165	-11.37	1	74.78	(M+H)+
